# Long-term cardiovascular outcomes of COVID-19

**DOI:** 10.1038/s41591-022-01689-3

**Published:** 2022-02-07

**Authors:** Yan Xie, Evan Xu, Benjamin Bowe, Ziyad Al-Aly

**Affiliations:** 1 Clinical Epidemiology Center, Research and Development Service, VA St. Louis Health Care System, St. Louis, MO USA; 2 Veterans Research and Education Foundation of St. Louis, St. Louis, MO USA; 3 Department of Epidemiology and Biostatistics, College for Public Health and Social Justice, Saint Louis University, St. Louis, MO USA; 4 Saint Louis University School of Medicine, St. Louis, MO USA; 5 Department of Medicine, Washington University School of Medicine, St. Louis, MO USA; 6 Nephrology Section, Medicine Service, VA St. Louis Health Care System, St. Louis, MO USA; 7 Institute for Public Health, Washington University in St. Louis, St. Louis, MO USA

**Keywords:** Health services, Epidemiology, SARS-CoV-2, Cardiovascular diseases, Viral infection

## Abstract

The cardiovascular complications of acute coronavirus disease 2019 (COVID-19) are well described, but the post-acute cardiovascular manifestations of COVID-19 have not yet been comprehensively characterized. Here we used national healthcare databases from the US Department of Veterans Affairs to build a cohort of 153,760 individuals with COVID-19, as well as two sets of control cohorts with 5,637,647 (contemporary controls) and 5,859,411 (historical controls) individuals, to estimate risks and 1-year burdens of a set of pre-specified incident cardiovascular outcomes. We show that, beyond the first 30 d after infection, individuals with COVID-19 are at increased risk of incident cardiovascular disease spanning several categories, including cerebrovascular disorders, dysrhythmias, ischemic and non-ischemic heart disease, pericarditis, myocarditis, heart failure and thromboembolic disease. These risks and burdens were evident even among individuals who were not hospitalized during the acute phase of the infection and increased in a graded fashion according to the care setting during the acute phase (non-hospitalized, hospitalized and admitted to intensive care). Our results provide evidence that the risk and 1-year burden of cardiovascular disease in survivors of acute COVID-19 are substantial. Care pathways of those surviving the acute episode of COVID-19 should include attention to cardiovascular health and disease.

## Abstract (web summary)

Individuals with COVID-19 are at increased long-term risk for a wide range of cardiovascular disorders, even for individuals who were not hospitalized during the acute phase of the infection.

## Main

Post-acute sequelae of severe acute respiratory syndrome coronavirus 2 (SARS-CoV-2)—the virus that causes coronavirus disease 2019 (COVID-19)—can involve the pulmonary and several extrapulmonary organs, including the cardiovascular system^1^. A few studies have investigated cardiovascular outcomes in the post-acute phase of the COVID-19; however, most were limited to hospitalized individuals (who represent the minority of people with COVID-19), and all had a short duration of follow-up and a narrow selection of cardiovascular outcomes^2–5^. A comprehensive assessment of post-acute COVID-19 sequelae of the cardiovascular system at 12 months is not yet available, and studies of post-acute COVID-19 sequelae across the spectrum of care settings of the acute infection (non-hospitalized, hospitalized and admitted to intensive care) are also lacking. Addressing this knowledge gap will inform post-acute COVID-19 care strategies.

In this study, we used the US Department of Veterans Affairs national healthcare databases to build a cohort of 153,760 US veterans who survived the first 30 d of COVID-19 and two control groups: a contemporary cohort consisting of 5,637,647 users of the US Veterans Health Administration (VHA) system with no evidence of SARS-CoV-2 infection and a historical cohort (pre-dating the COVID-19 pandemic) consisting of 5,859,411 non-COVID-19-infected VHA users during 2017. These cohorts were followed longitudinally to estimate the risks and 12-month burdens of pre-specified incident cardiovascular outcomes in the overall cohort and according to care setting of the acute infection (non-hospitalized, hospitalized and admitted to intensive care).

## Results

**Fig. 1:**
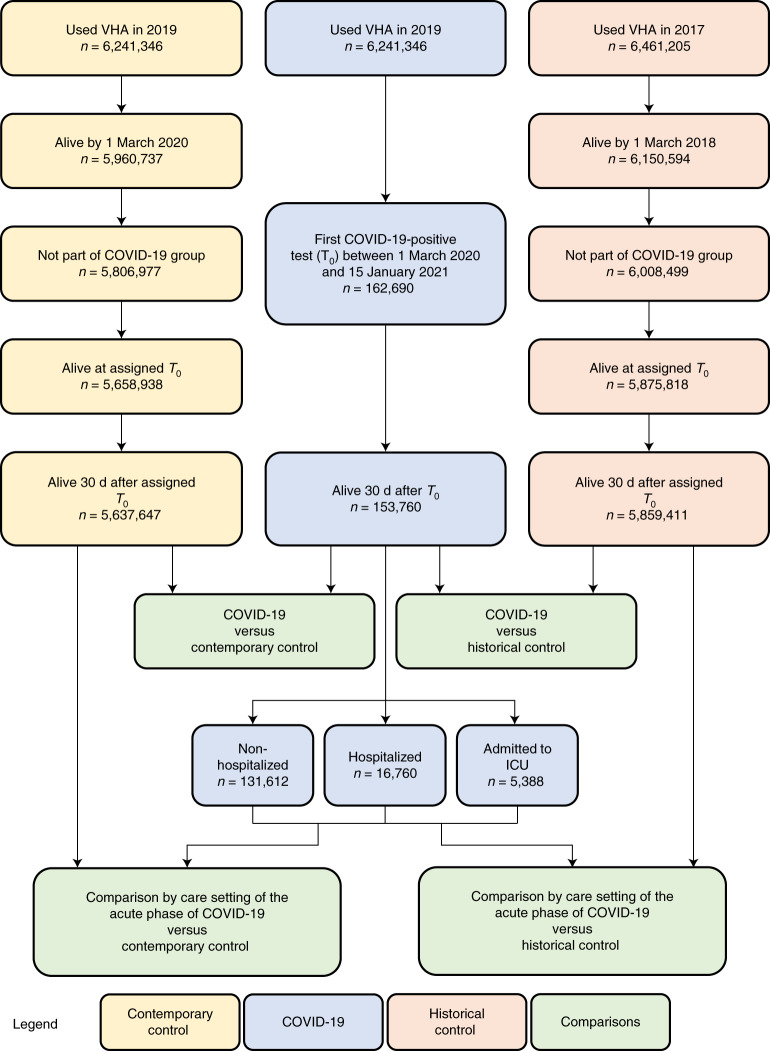
Flowchart of cohort construction. Cohort construction for COVID-19 group (blue), contemporary control group (yellow) and historical control group (orange). Comparisons between groups are presented in green.

There were 153,760, 5,637,647 and 5,859,411 participants in the COVID-19, contemporary control and historical control groups, respectively (Fig. 1). Median follow-up time in the COVID-19, contemporary control and historical control groups was 347 (interquartile range, 317–440), 348 (318–441) and 347 (317–440) d, respectively. The COVID-19, contemporary control and historical control groups had 159,366, 5,854,288 and 6,082,182 person-years of follow-up, respectively, altogether corresponding to 12,095,836 person-years of follow-up. The demographic and health characteristics of the COVID-19, contemporary control and historical control groups before and after weighting are presented in Supplementary Tables 1 and 2, respectively.

### Incident cardiovascular diseases in COVID-19 versus contemporary control

**Extended Data Fig. 1:**
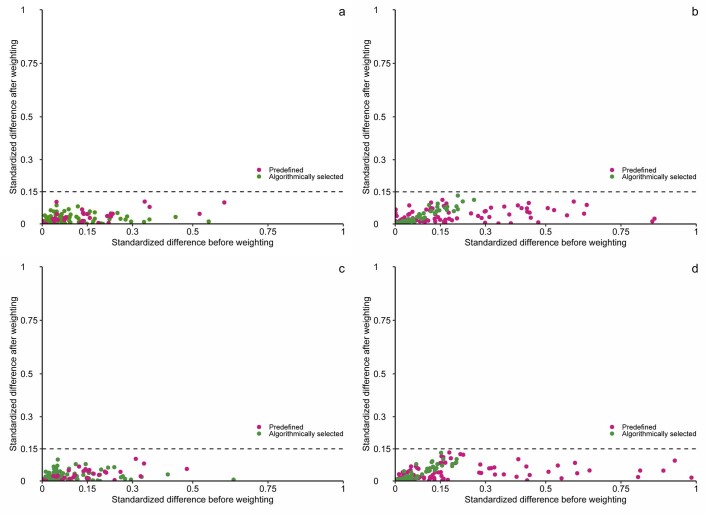
Standardized mean difference of predefined and algorithmically selected high dimensional variables. **a**. between COVID-19 and contemporary control cohorts; **b**. between COVID-19 categorized by care setting of the acute infection (non-hospitalized, hospitalized, and admitted to intensive care) and contemporary control cohorts; **c**. between COVID-19 and historical control cohorts; **d**. between COVID-19 categorized by care setting of the acute infection (non-hospitalized, hospitalized, and admitted to intensive care) and historical control cohorts. Standardized difference less than 0.15 is considered good balance.

Assessment of covariate balance after application of inverse probability weighting suggested that covariates were well balanced (Extended Data Fig. 1a).

**Fig. 2:**
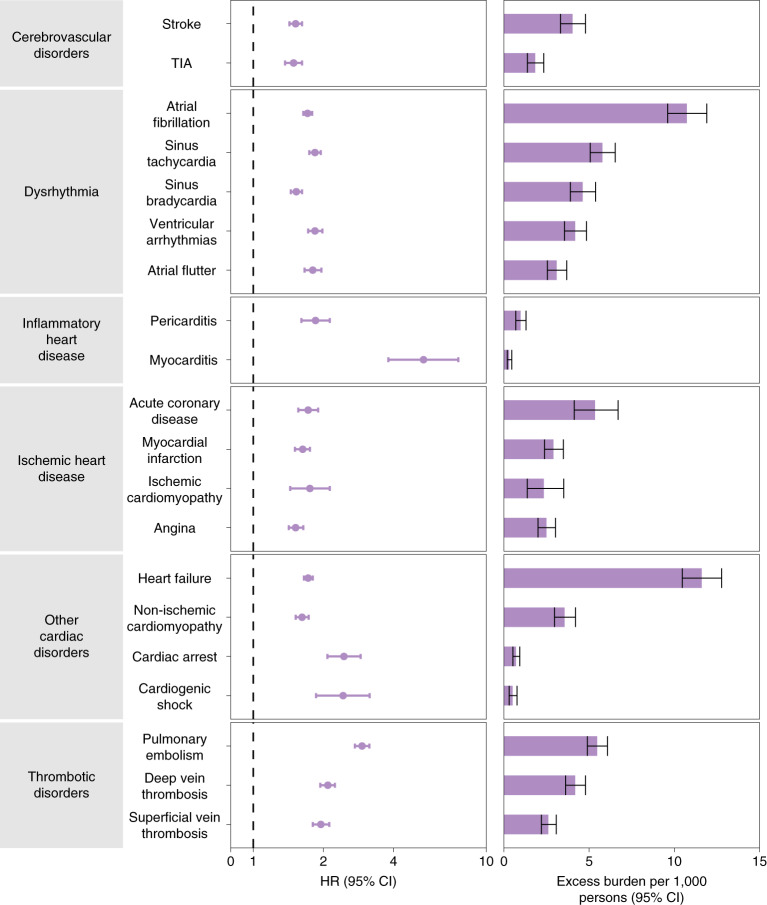
Risks and 12-month burdens of incident post-acute COVID-19 cardiovascular outcomes compared with the contemporary control cohort. Outcomes were ascertained 30 d after the COVID-19-positive test until the end of follow-up. COVID-19 cohort (*n* = 153,760) and contemporary control cohort (*n* = 5,637,647). Adjusted HRs and 95% CIs are presented. The length of the bar represents the excess burden per 1,000 persons at 12 months, and associated 95% CIs are also shown.

**Fig. 3:**
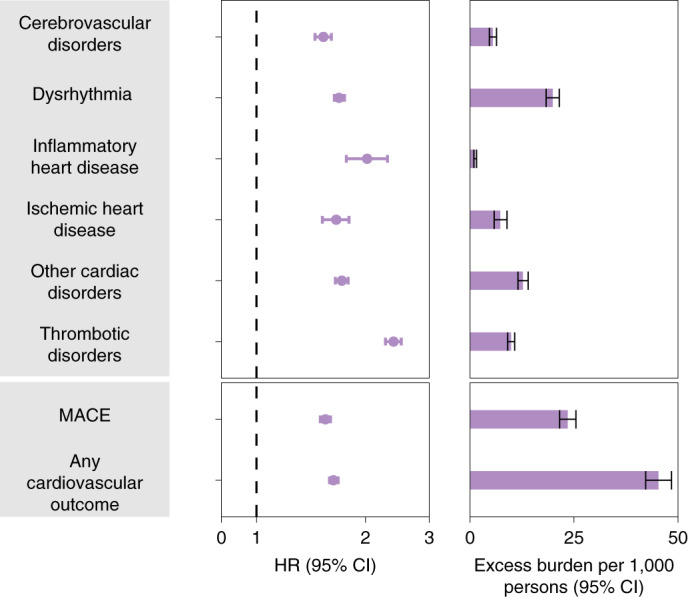
Risks and 12-month burdens of incident post-acute COVID-19 composite cardiovascular outcomes compared with the contemporary control cohort. Composite outcomes consisted of cerebrovascular disorders (stroke and TIA), dysrhythmias (atrial fibrillation, sinus tachycardia, sinus bradycardia, ventricular arrhythmias and atrial flutter), inflammatory heart disease (pericarditis and myocarditis), ischemic heart disease (acute coronary disease, myocardial infarction, ischemic cardiomyopathy and angina), other cardiac disorders (heart failure, non-ischemic cardiomyopathy, cardiac arrest and cardiogenic shock), thrombotic disorders (pulmonary embolism, deep vein thrombosis and superficial vein thrombosis), MACE (all-cause mortality, stroke and myocardial infarction) and any cardiovascular outcome (incident occurrence of any cardiovascular outcome studied). Outcomes were ascertained 30 d after the COVID-19-positive test until the end of follow-up. COVID-19 cohort (*n* = 153,760) and contemporary control cohort (*n* = 5,637,647). Adjusted HRs and 95% CIs are presented. The length of the bar represents the excess burden per 1,000 persons at 12 months, and associated 95% CIs are also shown.

We estimated the risks of a set of pre-specified cardiovascular outcomes in COVID-19 versus contemporary control; we also estimated the adjusted excess burden of cardiovascular outcomes due to COVID-19 per 1,000 persons at 12 months on the basis of the difference between the estimated incidence rate in individuals with COVID-19 and the contemporary control group. Risks and burdens of individual cardiovascular outcomes are provided in Fig. 2 and Supplementary Table 3 and are discussed below. Risks and burdens of the composite endpoints are provided in Fig. 3 and Supplementary Table 3.

#### Cerebrovascular disorders

People who survived the first 30 d of COVID-19 exhibited increased risk of stroke (hazard ratio (HR) = 1.52 (1.43, 1.62); burden 4.03 (3.32, 4.79) per 1,000 persons at 12 months; for all HRs and burdens, parenthetical ranges refer to 95% confidence intervals (CIs)) and transient ischemic attacks (TIA) (HR = 1.49 (1.37, 1.62); burden 1.84 (1.38, 2.34)). The risks and burdens of a composite of these cerebrovascular outcomes were 1.53 (1.45, 1.61) and 5.48 (4.65, 6.35).

#### Dysrhythmias

There were increased risks of atrial fibrillation (HR = 1.71 (1.64, 1.79); burden 10.74 (9.61, 11.91)), sinus tachycardia (HR = 1.84 (1.74, 1.95); burden 5.78 (5.07, 6.53)), sinus bradycardia (HR = 1.53 (1.45, 1.62); burden 4.62 (3.90, 5.38)), ventricular arrhythmias (HR = 1.84 (1.72, 1.98); burden 4.18 (3.56, 4.85)); and atrial flutter (HR = 1.80 (1.66, 1.96); burden 3.10 (2.55, 3.69)). The risks and burdens of a composite of these dysrhythmia outcomes were 1.69 (1.64, 1.75), and 19.86 (18.31, 21.46).

#### Inflammatory disease of the heart or pericardium

Inflammatory disease of the heart or pericardium included pericarditis (HR = 1.85 (1.61, 2.13)); burden 0.98 (0.70, 1.30) and myocarditis (HR = 5.38 (3.80, 7.59); burden 0.31 (0.20, 0.46)). The risks and burdens of a composite of these inflammatory diseases of the heart or pericardium were 2.02 (1.77, 2.30) and 1.23 (0.93, 1.57).

#### Ischemic heart disease

Ischemic heart disease included acute coronary disease (HR = 1.72 (1.56, 1.90); burden 5.35 (4.13, 6.70)), myocardial infarction (HR = 1.63 (1.51, 1.75); burden 2.91 (2.38, 3.49)), ischemic cardiomyopathy (HR = 1.75 (1.44, 2.13); burden 2.34 (1.37, 3.51)) and angina (HR = 1.52 (1.42, 1.64); burden 2.50 (2.00, 3.03)). The risks and burdens of a composite of these ischemic heart disease outcomes were 1.66 (1.52, 1.80) and 7.28 (5.80, 8.88).

#### Other cardiovascular disorders

Other cardiovascular disorders included heart failure (HR = 1.72 (1.65, 1.80); burden 11.61 (10.47, 12.78)), non-ischemic cardiomyopathy (HR = 1.62 (1.52, 1.73); burden 3.56 (2.97, 4.20)), cardiac arrest (HR = 2.45 (2.08, 2.89); burden 0.71 (0.53, 0.93)) and cardiogenic shock (HR = 2.43 (1.86, 3.16); burden 0.51 (0.31, 0.77)). The risks and burdens of a composite of these other cardiovascular disorders were 1.72 (1.65, 1.79) and 12.72 (11.54, 13.96).

#### Thromboembolic disorders

Thromboembolic disorders included pulmonary embolism (HR = 2.93 (2.73, 3.15); burden 5.47 (4.90, 6.08)); deep vein thrombosis (HR = 2.09 (1.94, 2.24); burden 4.18 (3.62, 4.79)) and superficial vein thrombosis (HR = 1.95 (1.80, 2.12); burden 2.61 (2.20, 3.07)). The risks and burdens of a composite of these thromboembolic disorders were 2.39 (2.27, 2.51) and 9.88 (9.05, 10.74).

#### Additional composite endpoints

We then examined the risks and burdens of two composite endpoints, including major adverse cardiovascular event (MACE)—a composite of myocardial infarction, stroke and all-cause mortality—and any cardiovascular outcome (defined as the occurrence of any incident pre-specified cardiovascular outcome included in this study). Compared to the contemporary control group, there were increased risks and burdens of MACE (HR = 1.55 (1.50, 1.60); burden 23.48 (21.54, 25.48)) and any cardiovascular outcome (HR = 1.63 (1.59, 1.68); burden 45.29 (42.22, 48.45)).

#### Subgroup analyses

**Fig. 4:**
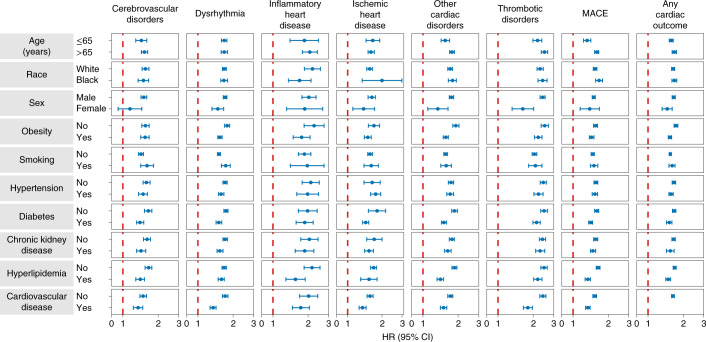
Subgroup analyses of the risks of incident post-acute COVID-19 composite cardiovascular outcomes compared with the contemporary control cohort. Composite outcomes consisted of cerebrovascular disorders (stroke and TIA), dysrhythmias (atrial fibrillation, sinus tachycardia, sinus bradycardia, ventricular arrhythmias and atrial flutter), inflammatory heart disease (pericarditis and myocarditis), ischemic heart disease (acute coronary disease, myocardial infarction, ischemic cardiomyopathy and angina), other cardiac disorders (heart failure, non-ischemic cardiomyopathy, cardiac arrest and cardiogenic shock), thrombotic disorders (pulmonary embolism, deep vein thrombosis and superficial vein thrombosis), MACE (all-cause mortality, stroke and myocardial infarction) and any cardiovascular outcome (incident occurrence of any cardiovascular outcome studied). Outcomes were ascertained 30 d after the COVID-19-positive test until the end of follow-up. COVID-19 cohort (*n* = 153,760) and contemporary control cohort (*n* = 5,637,647). Adjusted HRs and 95% CIs are presented.

**Extended Data Fig. 2:**
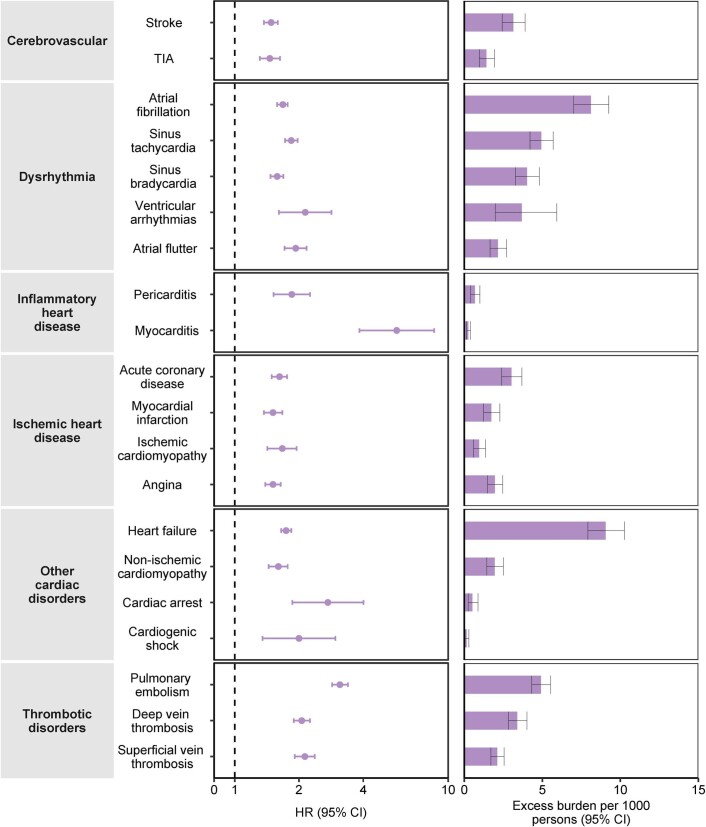
Risks and 12-month burdens of incident post-acute COVID-19 cardiovascular outcomes in participants without any history of cardiovascular outcomes prior to COVID-19 exposure compared to the contemporary control cohort. Outcomes were ascertained 30 days after the COVID-19 positive test until the end of follow-up. COVID-19 cohort without any history of cardiovascular outcomes (N = 126,575) and contemporary control cohort without any history of cardiovascular outcomes (N = 5,010,542). Adjusted hazard ratios and 95% confidence intervals are presented. Length of the bar represents the excess burden per 1000 persons at 12 months and associated 95% confidence intervals are also shown. TIA, transient ischemic attack.

**Extended Data Fig. 3:**
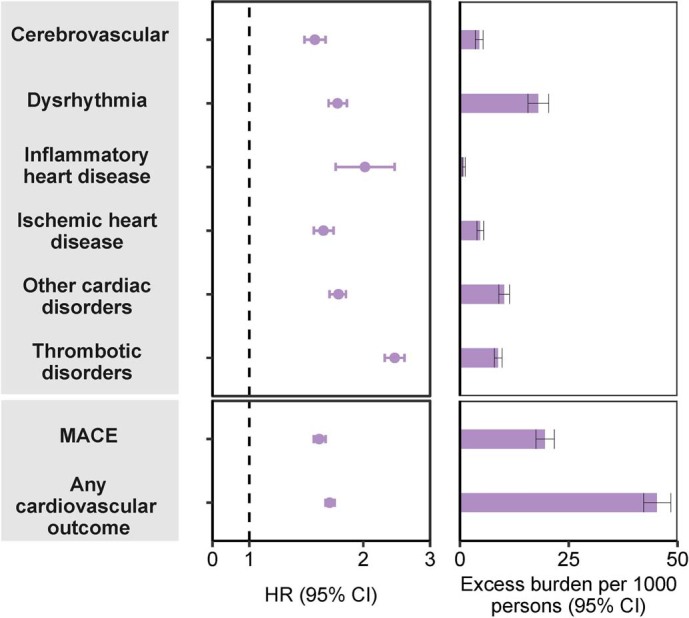
Risks and 12-month burdens of incident post-acute COVID-19 composite cardiovascular outcomes in participants without any history of cardiovascular outcomes prior to COVID-19 exposure compared to the contemporary control cohort. Composite outcomes consisted of cerebrovascular (stroke and TIA), dysrhythmias (atrial fibrillation, sinus tachycardia, sinus bradycardia, ventricular arrhythmias, and atrial flutter), inflammatory heart disease (pericarditis, myocarditis), ischemic heart disease (acute coronary disease, myocardial infarction, ischemic cardiomyopathy, and angina), other cardiac disorders (heart failure, non-ischemic cardiomyopathy, cardiac arrest, and cardiogenic shock), thrombotic disorders (pulmonary embolism, deep vein thrombosis, and superficial vein thrombosis), MACE (all-cause mortality, stroke, and myocardial infarction), and any cardiovascular outcome (incident occurrence of any cardiovascular outcome studied). Outcomes were ascertained 30 days after the COVID-19 positive test until the end of follow-up. COVID-19 cohort without any history of cardiovascular outcomes (N = 126,575) and contemporary control cohort without any history of cardiovascular outcomes (N = 5,010,542). Adjusted hazard ratios and 95% confidence intervals are presented. Length of the bar represents the excess burden per 1000 persons at 12 months and associated 95% confidence intervals are also shown. MACE, major adverse cardiac events; TIA, transient ischemic attack.

We examined the risks of incident composite cardiovascular outcomes in subgroups based on age, race, sex, obesity, smoking, hypertension, diabetes, chronic kidney disease, hyperlipidemia and cardiovascular disease. The risks of incident composite cardiovascular outcomes were evident in all subgroups (Fig. 4 and Supplementary Table 4),

We examined the risks and burdens of the pre-specified outcomes in a cohort of people without any cardiovascular disease at baseline; the results were consistent with those shown in the primary analyses (Extended Data Figs. 2 and 3 and Supplementary Table 5).

### Incident cardiovascular diseases in COVID-19 versus contemporary control by care setting of the acute infection

**Fig. 5:**
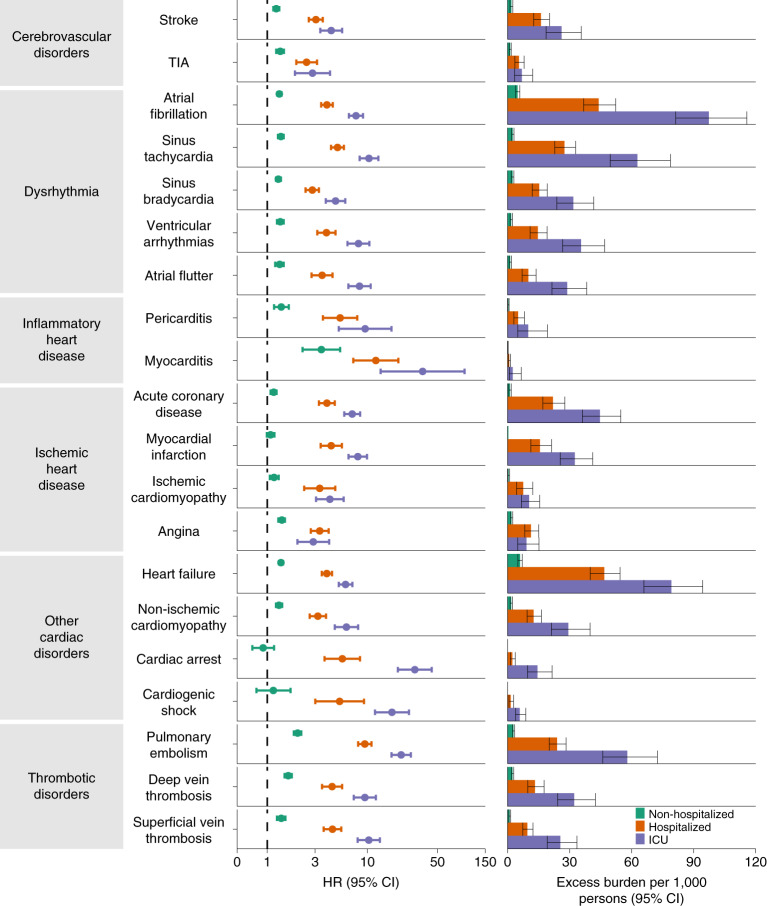
Risks and 12-month burdens of incident post-acute COVID-19 cardiovascular outcomes compared with the contemporary control cohort by care setting of the acute infection. Risks and burdens were assessed at 12 months in mutually exclusive groups comprising non-hospitalized individuals with COVID-19 (green), individuals hospitalized for COVID-19 (orange) and individuals admitted to intensive care for COVID-19 during the acute phase (first 30 d) of COVID-19 (blue). Outcomes were ascertained 30 d after the COVID-19-positive test until the end of follow-up. The contemporary control cohort served as the referent category. Within the COVID-19 cohort, non-hospitalized (*n* = 131,612), hospitalized (*n* = 16,760), admitted to intensive care (*n* = 5,388) and contemporary control cohort (*n* = 5,637,647). Adjusted HRs and 95% CIs are presented. The length of the bar represents the excess burden per 1,000 persons at 12 months, and related 95% CIs were also presented.

**Fig. 6:**
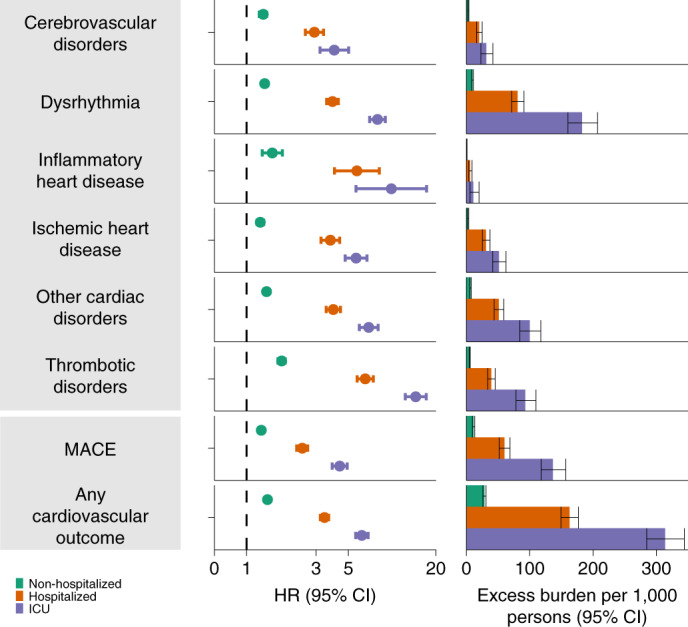
Risks and 12-month burdens of incident post-acute COVID-19 composite cardiovascular outcomes compared with the contemporary control cohort by care setting of the acute infection. Risks and burdens were assessed at 12 months in mutually exclusive groups comprising non-hospitalized individuals with COVID-19 (green), individuals hospitalized for COVID-19 (orange) and individuals admitted to intensive care for COVID-19 during the acute phase (first 30 d) of COVID-19 (blue). Composite outcomes consisted of cerebrovascular disorders (stroke and TIA), dysrhythmias (atrial fibrillation, sinus tachycardia, sinus bradycardia, ventricular arrhythmias and atrial flutter), inflammatory heart disease (pericarditis and myocarditis), ischemic heart disease (acute coronary disease, myocardial infarction, ischemic cardiomyopathy and angina), other cardiac disorders (heart failure, non-ischemic cardiomyopathy, cardiac arrest and cardiogenic shock), thrombotic disorders (pulmonary embolism, deep vein thrombosis and superficial vein thrombosis), MACE (all-cause mortality, stroke and myocardial infarction) and any cardiovascular outcome (incident occurrence of any cardiovascular outcome studied). Outcomes were ascertained 30 d after the COVID-19-positive test until the end of follow-up. The contemporary control cohort served as the referent category. Within the COVID-19 cohort, non-hospitalized (*n* = 131,612), hospitalized (*n* = 16,760), admitted to intensive care (*n* = 5,388) and contemporary control cohort (*n* = 5,637,647). Adjusted HRs and 95% CIs are presented. The length of the bar represents the excess burden per 1,000 persons at 12 months, and related 95% CIs were also presented.

We further examined the risks and burdens of cardiovascular diseases in mutually exclusive groups by the care setting of the acute infection (that is, whether people were non-hospitalized (*n* = 131,612), hospitalized (*n* = 16,760) or admitted to intensive care (*n* = 5,388) during the acute phase of COVID-19); demographic and health characteristics of these groups before weighting can be found in Supplementary Table 6 and after weighting in Supplementary Table 7. Assessment of covariate balance after application of weights suggested that covariates were well balanced (Extended Data Fig. 1b). Compared to the contemporary control group, the risks and 12-month burdens of the pre-specified cardiovascular outcomes increased according to the severity of the acute infection (Fig. 5 and Supplementary Table 8); results for the composite outcomes are shown in Fig. 6 and Supplementary Table 8.

### Incident cardiovascular diseases in COVID-19 versus historical control

**Extended Data Fig. 4:**
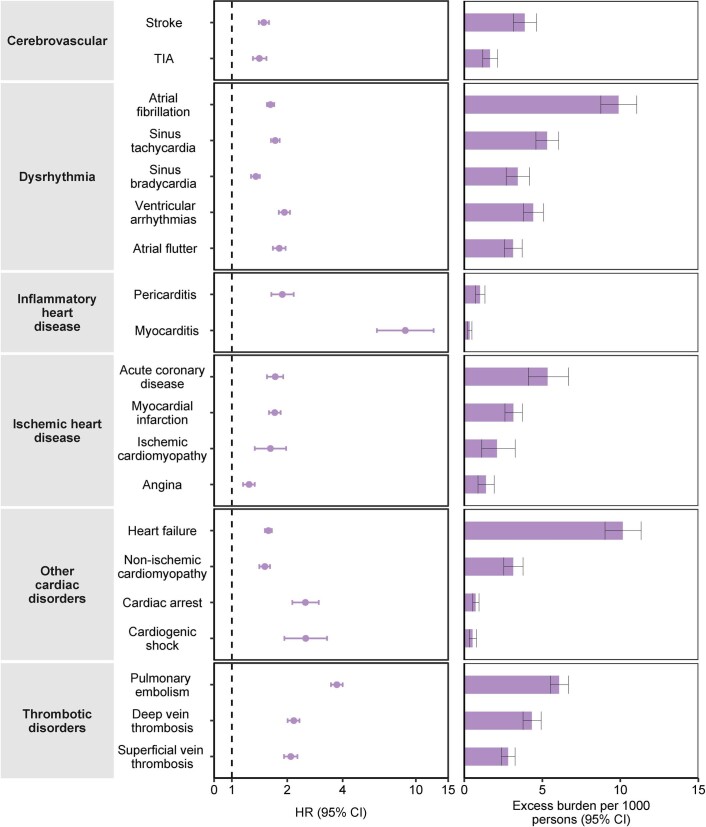
Risks and 12-month burdens of incident post-acute COVID-19 cardiovascular outcomes compared to the historical control cohort. Outcomes were ascertained 30 days after the COVID-19 positive test until the end of follow-up. COVID-19 cohort (N = 153,760) and historical control cohort (N = 5,859,411). Adjusted hazard ratios and 95% confidence intervals are presented. Length of the bar represents the excess burden per 1000 persons at 12 months and associated 95% confidence intervals are also shown. TIA, transient ischemic attack.

**Extended Data Fig. 5:**
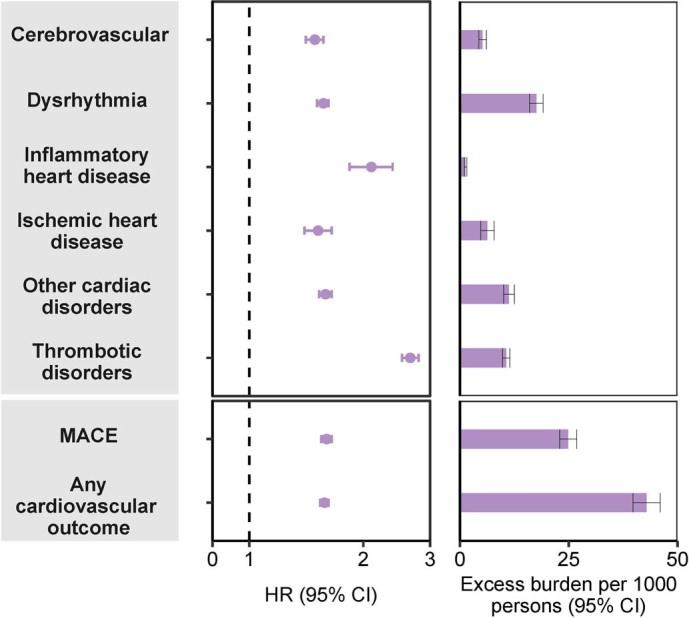
Risks and 12-month burdens of incident post-acute COVID-19 composite cardiovascular outcomes compared to the historical control cohort. Composite outcomes consisted of cerebrovascular (stroke and TIA), dysrhythmias (atrial fibrillation, sinus tachycardia, sinus bradycardia, ventricular arrhythmias, and atrial flutter), inflammatory heart disease (pericarditis, myocarditis), ischemic heart disease (acute coronary disease, myocardial infarction, ischemic cardiomyopathy, and angina), other cardiac disorders (heart failure, non-ischemic cardiomyopathy, cardiac arrest, and cardiogenic shock), thrombotic disorders (pulmonary embolism, deep vein thrombosis, and superficial vein thrombosis), MACE (all-cause mortality, stroke, and myocardial infarction), and any cardiovascular outcome (incident occurrence of any cardiovascular outcome studied). Outcomes were ascertained 30 days after the COVID-19 positive test until the end of follow-up. COVID-19 cohort (N = 153,760) and historical control cohort (N = 5,859,411). Adjusted hazard ratios and 95% confidence intervals are presented. Length of the bar represents the excess burden per 1000 persons at 12 months and associated 95% confidence intervals are also shown. MACE, major adverse cardiac events; TIA, transient ischemic attack.

**Extended Data Fig. 6:**
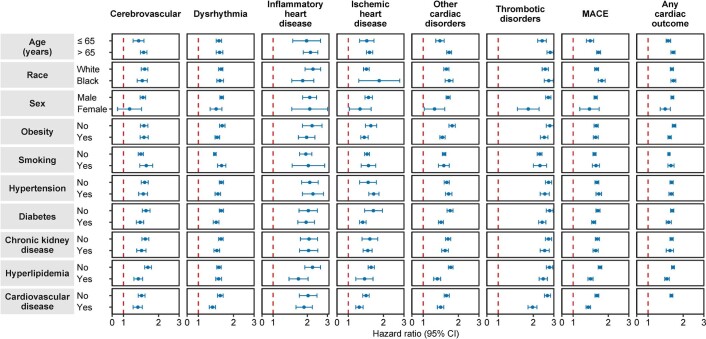
Subgroup analyses of the risks of incident post-acute COVID-19 composite cardiovascular outcomes compared to the historical control cohort. Composite outcomes consisted of cerebrovascular (stroke and TIA), dysrhythmias (atrial fibrillation, sinus tachycardia, sinus bradycardia, ventricular arrhythmias, and atrial flutter), inflammatory heart disease (pericarditis, myocarditis), ischemic heart disease (acute coronary disease, myocardial infarction, ischemic cardiomyopathy, and angina), other cardiac disorders (heart failure, non-ischemic cardiomyopathy, cardiac arrest, and cardiogenic shock), thrombotic disorders (pulmonary embolism, deep vein thrombosis, and superficial vein thrombosis), MACE (all-cause mortality, stroke, and myocardial infarction), and any cardiovascular outcome (incident occurrence of any cardiovascular outcome studied). Outcomes were ascertained 30 days after the COVID-19 positive test until the end of follow-up. COVID-19 cohort (N = 153,760) and historical control cohort (N = 5,859,411). Adjusted hazard ratios and 95% confidence intervals are presented. MACE, major adverse cardiac events; TIA, transient ischemic attack.

**Extended Data Fig. 7:**
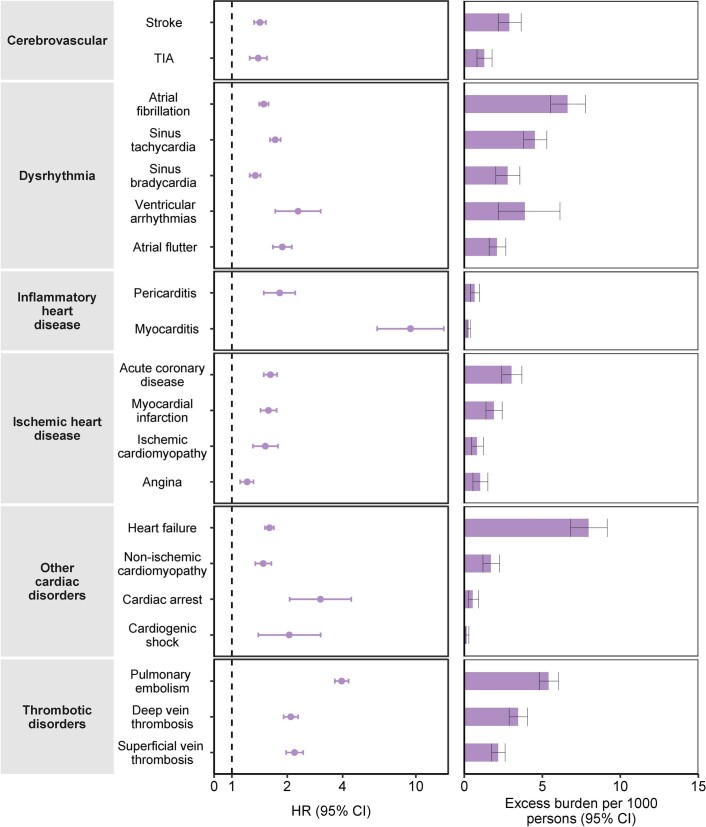
Risks and 12-month burdens of incident post-acute COVID-19 cardiovascular outcomes in participants without any history of cardiovascular outcomes prior to COVID-19 exposure compared to the historical control cohort. Outcomes were ascertained 30 days after the COVID-19 positive test until the end of follow-up. COVID-19 cohort without any history of cardiovascular outcomes (N = 126,575) and historical control cohort without any history of cardiovascular outcomes (N = 5,188,992). Adjusted hazard ratios and 95% confidence intervals are presented. Length of the bar represents the excess burden per 1000 persons at 12 months and associated 95% confidence intervals are also shown. TIA, transient ischemic attack.

**Extended Data Fig. 8:**
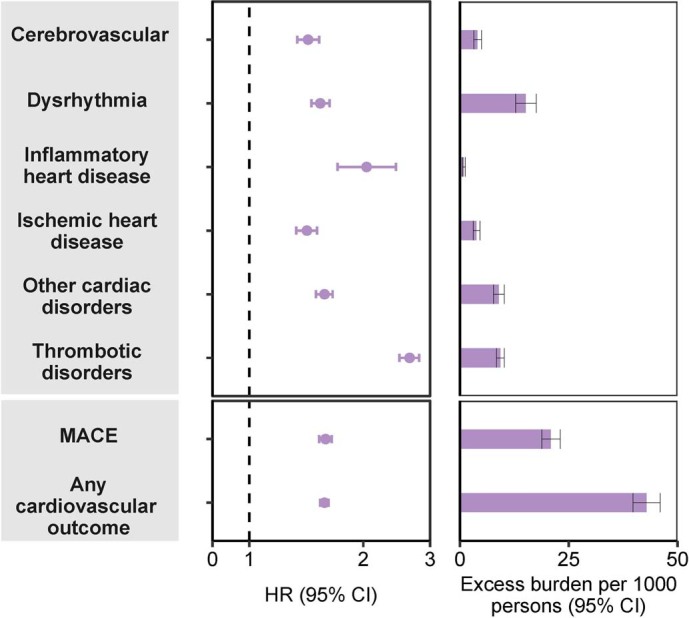
Risks and 12-month burdens of incident post-acute COVID-19 composite cardiovascular outcomes in participants without any history of cardiovascular outcomes prior to COVID-19 exposure compared to the historical control cohort. Composite outcomes consisted of cerebrovascular (stroke and TIA), dysrhythmias (atrial fibrillation, sinus tachycardia, sinus bradycardia, ventricular arrhythmias, and atrial flutter), inflammatory heart disease (pericarditis, myocarditis), ischemic heart disease (acute coronary disease, myocardial infarction, ischemic cardiomyopathy, and angina), other cardiac disorders (heart failure, non-ischemic cardiomyopathy, cardiac arrest, and cardiogenic shock), thrombotic disorders (pulmonary embolism, deep vein thrombosis, and superficial vein thrombosis), MACE (all-cause mortality, stroke, and myocardial infarction), and any cardiovascular outcome (incident occurrence of any cardiovascular outcome studied). Outcomes were ascertained 30 days after the COVID-19 positive test until the end of follow-up. COVID-19 cohort without any history of cardiovascular outcomes (N = 126,575) and historical control cohort without any history of cardiovascular outcomes (N = 5,188,992). Adjusted hazard ratios and 95% confidence intervals are presented. Length of the bar represents the excess burden per 1000 persons at 12 months and associated 95% confidence intervals are also shown. MACE, major adverse cardiac events; TIA, transient ischemic attack.

**Extended Data Fig. 9:**
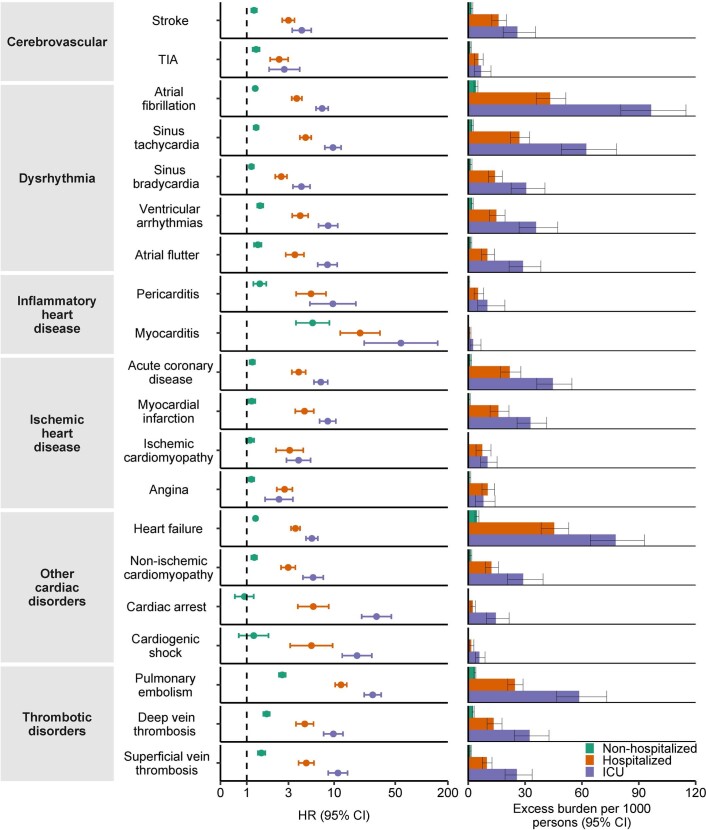
Risks and 12-month burdens of incident post-acute COVID-19 cardiovascular outcomes compared to the historical control cohort by care setting of the acute infection. Risks and burdens were assessed at 12 months in mutually exclusive groups comprising non-hospitalized individuals with COVID-19 (green), individuals hospitalized for COVID-19 (orange), and individuals admitted to intensive care for COVID-19 during the acute phase (first 30 days) of COVID-19 (blue). Outcomes were ascertained 30 days after the COVID-19 positive test until the end of follow-up. The historical control cohort served as the referent category. Within the COVID-19 cohort, non-hospitalized (N = 131,612), hospitalized (N = 16,760); admitted to intensive care (N = 5,388); and historical control cohort (N = 5,859,411). Adjusted hazard ratios and 95% confidence intervals are presented. Length of the bar represents the excess burden per 1000 persons at 12 months and related 95% confidence intervals were also presented. TIA, transient ischemic attack.

**Extended Data Fig. 10:**
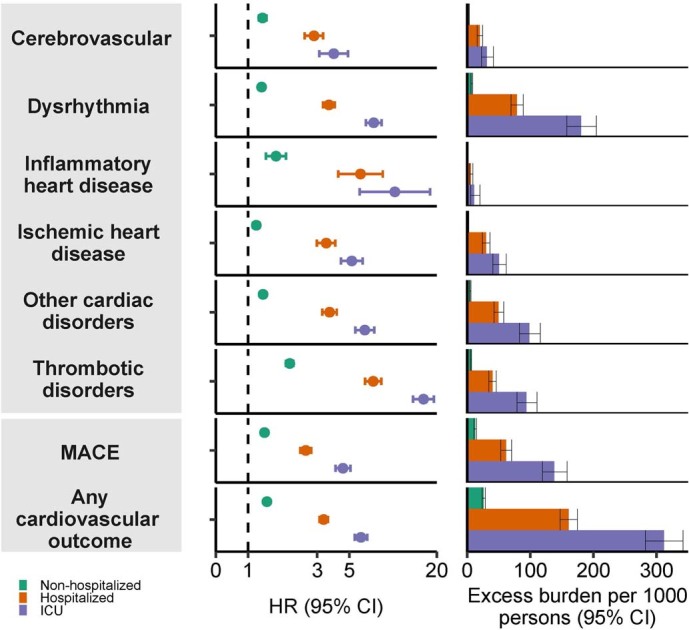
Risks and 12-month burdens of incident post-acute COVID-19 composite cardiovascular outcomes compared to the historical control cohort by care setting of the acute infection. Risks and burdens were assessed at 12 months in mutually exclusive groups comprising non-hospitalized individuals with COVID-19 (green), individuals hospitalized for COVID-19 (orange), and individuals admitted to intensive care for COVID-19 during the acute phase (first 30 days) of COVID-19 (blue). Composite outcomes consisted of cerebrovascular (stroke and TIA), dysrhythmias (atrial fibrillation, sinus tachycardia, sinus bradycardia, ventricular arrhythmias, and atrial flutter), inflammatory heart disease (pericarditis, myocarditis), ischemic heart disease (acute coronary disease, myocardial infarction, ischemic cardiomyopathy, and angina), other cardiac disorders (heart failure, non-ischemic cardiomyopathy, cardiac arrest, and cardiogenic shock), thrombotic disorders (pulmonary embolism, deep vein thrombosis, and superficial vein thrombosis), MACE (all-cause mortality, stroke, and myocardial infarction), and any cardiovascular outcome (incident occurrence of any cardiovascular outcome studied). Outcomes were ascertained 30 days after the COVID-19 positive test until the end of follow-up. The historical control cohort served as the referent category. Within the COVID-19 cohort, non-hospitalized (N = 131,612), hospitalized (N = 16,760); admitted to intensive care (N = 5,388); and historical control cohort (N = 5,859,411). Adjusted hazard ratios and 95% confidence intervals are presented. Length of the bar represents the excess burden per 1000 persons at 12 months and related 95% confidence intervals were also presented. MACE, major adverse cardiac events; TIA, transient ischemic attack.

We then examined the associations between COVID-19 and the pre-specified outcomes in analyses considering a historical control group as the referent category; the characteristics of the exposure groups were balanced after weighting (Extended Data Fig. 1c and Supplementary Table 2). The results were consistent with analyses using the contemporary control as the referent category and showed increased risks and associated burdens of the pre-specified outcomes in comparisons of COVID-19 versus the overall historical control group (Extended Data Figs. 4 and 5 and Supplementary Table 9). Using the historical control as the referent category, we examined the risks in subgroups and separately in people without any prior cardiovascular disease; the results were consistent with those undertaken versus the contemporary control (Extended Data Figs. 6–8 and Supplementary Tables 10 and 11). Associations between COVID-19 and our pre-specified outcomes based on care setting of the acute infection were also assessed using the historical control group as the referent category; demographic and clinical characteristics are presented before weighting in Supplementary Table 12 and after weighting in Supplementary Table 13. Characteristics of the exposure groups were balanced after weighting (Extended Data Fig. 1d). The risks and 12-month burdens of the pre-specified outcomes by care setting of the acute infection were also consistent with those shown in analyses considering COVID-19 versus contemporary control (Extended Data Figs. 9 and 10 and Supplementary Table 14).

### Cardiovascular diseases before and after COVID-19

To better understand the change in the relative rates of incident cardiovascular outcomes before and after the COVID-19 exposure, we developed a difference-in-differences analysis to estimate the adjusted incident rate ratios of the cardiovascular outcomes relative to both the contemporary and historical control groups in the pre-COVID-19 and post-COVID-19 exposure periods. The results showed that the adjusted incident rate ratios of cardiovascular outcomes in the post-COVID-19 exposure period were significantly higher than those in the pre-exposure period (ratios of incident rate ratios for all cardiovascular outcomes were significantly higher than 1) and exhibited a graded increase by severity of the acute phase of the disease (Supplementary Tables 15–18).

### Sensitivity analyses

We tested robustness of results in several sensitivity analyses involving the outcomes of MACE and any cardiovascular outcome (Supplementary Tables 17 and 18). The sensitivity analyses were performed in comparisons involving COVID-19 versus the contemporary control and COVID-19 versus the historical control and, additionally, COVID-19 by care setting versus both controls. (1) To test whether the inclusion of additional algorithmically selected covariates would challenge the robustness of study results, we selected and used 300 high-dimensional variables (instead of the 100 used in the primary analyses) to construct the inverse probability weighting. (2) We then also tested the results in models specified to include only pre-defined covariates (that is, without inclusion of algorithmically selected covariates) to build the inverse probability weighting. Finally, (3) we changed the analytic approach by using the doubly robust method (instead of the inverse weighting method used in primary analyses) to estimate the magnitude of the associations between COVID-19 exposure and the pre-specified outcomes. All sensitivity analyses yielded results consistent with those produced using the primary approach (Supplementary Tables 19 and 20).

### Risk of myocarditis and pericarditis without COVID-19 vaccination

Because some COVID-19 vaccines might be associated with a very rare risk of myocarditis or pericarditis, and to eliminate any putative contribution of potential vaccine exposure to the outcomes of myocarditis and pericarditis in this study, we conducted two analyses. First, we censored cohort participants at the time of receiving the first dose of any COVID-19 vaccine. Second, we adjusted for vaccination as a time-varying covariate. Both analyses were conducted versus both the contemporary and historical control groups. The results suggested that COVID-19 was associated with increased risk of myocarditis and pericarditis in both analyses (Supplementary Tables 21–24).

### Positive and negative outcome controls

To assess whether our data and analytic approach would reproduce known associations, we examined the association between COVID-19 and the risk of fatigue (known to be a signature sequela of post-acute COVID-19) as a positive outcome control. The results suggested that COVID-19 was associated with a higher risk of fatigue (Supplementary Table 25).

We then examined the association between COVID-19 and a battery of seven negative-outcome controls where no prior knowledge suggests that an association is expected. The results yielded no significant association between COVID-19 and any of the negative-outcome controls, which were consistent with a priori expectations (Supplementary Table 25).

### Negative-exposure controls

To further examine the robustness of our approach, we developed and tested a pair of negative-exposure controls. We hypothesized that receipt of influenza vaccination in odd-numbered and even-numbered calendar days between 1 March 2020 and 15 January 2021 would be associated with similar risks of the pre-specified cardiovascular outcomes examined in this analysis. We, therefore, tested the associations between receipt of influenza vaccine in even-numbered (*n* = 571,291) versus odd-numbered (*n* = 605,453) calendar days and the pre-specified cardiovascular outcomes. We used the same data sources, cohort design, analytical approach (including covariate specification and weighting method) and outcomes. The results suggest that receipt of influenza vaccination in odd-numbered calendar days versus even-numbered calendar days was not significantly associated with any of the pre-specified cardiovascular outcomes (Supplementary Table 26).

## Discussion

In this study involving 153,760 people with COVID-19, 5,637,647 contemporary controls and 5,859,411 historical controls—which, altogether, correspond to 12,095,836 person-years of follow-up—we provide evidence that, beyond the first 30 d of infection, people with COVID-19 exhibited increased risks and 12-month burdens of incident cardiovascular diseases, including cerebrovascular disorders, dysrhythmias, inflammatory heart disease, ischemic heart disease, heart failure, thromboembolic disease and other cardiac disorders. The risks were evident regardless of age, race, sex and other cardiovascular risk factors, including obesity, hypertension, diabetes, chronic kidney disease and hyperlipidemia; they were also evident in people without any cardiovascular disease before exposure to COVID-19, providing evidence that these risks might manifest even in people at low risk of cardiovascular disease. Our analyses of the risks and burdens of cardiovascular outcomes across care settings of the acute infection reveal two key findings: (1) that the risks and associated burdens were evident among those who were not hospitalized during the acute phase of the disease—this group represents the majority of people with COVID-19; and (2) that the risks and associated burdens exhibited a graded increase across the severity spectrum of the acute phase of COVID-19 (from non-hospitalized to hospitalized individuals to those admitted to intensive care). The risks and associated burdens were consistent in analyses considering the contemporary control group and, separately, the historical control group as the referent category. The difference-in-differences analyses, which are designed to further investigate the causality of study findings, show that the increased risks of post-acute COVID-19 cardiovascular outcomes are attributable sequelae to COVID-19 itself. The results were robust to challenge in multiple sensitivity analyses. Application of a positive-outcome control yielded results consistent with established knowledge; and testing of a battery of negative-outcome controls and negative-exposure controls yielded results consistent with a priori expectations. Taken together, our results show that 1-year risks and burdens of cardiovascular diseases among those who survive the acute phase of COVID-19 are substantial and span several cardiovascular disorders. Care strategies of people who survived the acute episode of COVID-19 should include attention to cardiovascular health and disease.

The broader implications of these findings are clear. Cardiovascular complications have been described in the acute phase of COVID-19 (refs. ^6–8^). Our study shows that the risk of incident cardiovascular disease extends well beyond the acute phase of COVID-19. First, the findings emphasize the need for continued optimization of strategies for primary prevention of SARS-CoV-2 infections; that is, the best way to prevent Long COVID and its myriad complications, including the risk of serious cardiovascular sequelae, is to prevent SARS-CoV-2 infection in the first place. Second, given the large and growing number of people with COVID-19 (more than 72 million people in the United States, more than 16 million people in the United Kingdom and more than 355 million people globally), the risks and 12-month burdens of cardiovascular diseases reported here might translate into a large number of potentially affected people around the world. Governments and health systems around the world should be prepared to deal with the likely significant contribution of the COVID-19 pandemic to a rise in the burden of cardiovascular diseases. Because of the chronic nature of these conditions, they will likely have long-lasting consequences for patients and health systems and also have broad implications on economic productivity and life expectancy. Addressing the challenges posed by Long COVID will require a much-needed, but so far lacking, urgent and coordinated long-term global response strategy^9,10^.

The mechanism or mechanisms that underlie the association between COVID-19 and development of cardiovascular diseases in the post-acute phase of the disease are not entirely clear^11,12^. Putative mechanisms include lingering damage from direct viral invasion of cardiomyocytes and subsequent cell death, endothelial cell infection and endotheliitis, transcriptional alteration of multiple cell types in heart tissue, complement activation and complement-mediated coagulopathy and microangiopathy, downregulation of ACE2 and dysregulation of the renin–angiotensin–aldosterone system, autonomic dysfunction, elevated levels of pro-inflammatory cytokines and activation of TGF-β signaling through the Smad pathway to induce subsequent fibrosis and scarring of cardiac tissue^11,13–17^. An aberrant persistent hyperactivated immune response, autoimmunity or persistence of the virus in immune-privileged sites has also been cited as putative explanations of extrapulmonary (including cardiovascular) post-acute sequelae of COVID-19 (refs. ^11,13,14,18^). Integration of the SARS-CoV-2 genome into DNA of infected human cells, which might then be expressed as chimeric transcripts fusing viral with cellular sequences, has also been hypothesized as a putative mechanism for continued activation of the immune-inflammatory-procoagulant cascade^19,20^. These mechanistic pathways might explain the range of post-acute COVID-19 cardiovascular sequelae investigated in this report. A deeper understanding of the biologic mechanisms will be needed to inform development of prevention and treatment strategies of the cardiovascular manifestations among people with COVID-19.

Our analyses censoring participants at time of vaccination and controlling for vaccination as a time-varying covariate show that the increased risk of myocarditis and pericarditis reported in this study is significant in people who were not vaccinated and is evident regardless of vaccination status.

This study has several strengths. We used the vast and rich national healthcare databases of the US Department of Veterans Affairs to build a large cohort of people with COVID-19. We designed the study cohort to investigate incident cardiovascular disease in the post-acute phase of the disease. We pre-specified a comprehensive list of cardiovascular outcomes. We examined the associations using two large control groups: a contemporary and a historical control; this approach allowed us to deduce that the associations between COVID-19 and risks of cardiovascular outcomes are not related to the broader temporal changes between the pre-pandemic and the pandemic eras but, rather, are related to exposure to COVID-19 itself. Our modeling approach included specification of 19 pre-defined variables selected based on established knowledge and 100 algorithmically selected variables from high-dimensional data domains, including diagnostic codes, prescription records and laboratory test results. We evaluated the associations across care settings of the acute infection. Our difference-in-differences approach further enhances the causal interpretation of study results. We challenged the robustness of results in multiple sensitivity analyses and successfully tested positive-outcome and negative-outcome controls and negative-exposure controls. We provided estimates of risk on both the ratio scale (HRs) and the absolute scale (burden per 1,000 persons at 12 months); the latter also reflects the contribution of baseline risk and provides an estimate of potential harm that is more easily explainable to the public than risk reported on the ratio scale (for example, HR).

This study has several limitations. The demographic composition of our cohort (majority White and male) might limit the generalizability of study findings. We used the electronic healthcare databases of the US Department of Veterans Affairs to conduct this study, and, although we used validated outcome definitions and took care to adjust the analyses for a large set of pre-defined and algorithmically selected variables, we cannot completely rule out misclassification bias and residual confounding. It is possible that some people might have had COVID-19 but were not tested for it; these people would have been enrolled in the control group and, if present in large numbers, might have biased the results toward the null. Our datasets do not include information on causes of death. Finally, as the pandemic, with all its dynamic features, continues to progress, as the virus continues to mutate and as new variants emerge, as treatment strategies of acute and post-acute COVID-19 evolve and as vaccine uptake improves, it is possible that the epidemiology of cardiovascular manifestations in COVID-19 might also change over time^21^.

In summary, using a national cohort of people with COVID-19, we show that risk and 12-month burden of incident cardiovascular disease are substantial and span several cardiovascular disease categories (ischemic and non-ischemic heart disease, dysrhythmias and others). The risks and burdens of cardiovascular disease were evident even among those whose acute COVID-19 did not necessitate hospitalization. Care pathways of people who survived the acute episode of COVID-19 should include attention to cardiovascular health and disease.

## Methods

### Setting

We used the electronic healthcare databases of the US Department of Veterans Affairs to conduct this study. The VHA, within the US Department of Veterans Affairs, provides healthcare to discharged veterans of the US armed forces. It operates the largest nationally integrated healthcare system in the United States, with 1,255 healthcare facilities (including 170 VA Medical Centers and 1,074 outpatient sites) located across the United States. All veterans who are enrolled with the VHA have access to the comprehensive medical benefits package of the VA (which includes preventative and health maintenance, outpatient care, inpatient hospital care, prescriptions, mental healthcare, home healthcare, primary care, specialty care, geriatric and extended care, medical equipment and prosthetics). The VA electronic healthcare databases are updated daily.

### Cohort

A flowchart of cohort construction is provided in Fig. 1. Of 6,241,346 participants who encountered the VHA in 2019, 162,690 participants who had a positive COVID-19 test between 1 March 2020 and 15 January 2021 were selected into the COVID-19 group. To examine post-acute outcomes, we then selected participants from the COVID-19 group who were alive 30 d after the date of the positive COVID-19 test (*n* = 153,760). The date of the COVID-19-positive test served as *T*_0_ for the COVID-19 group.

A contemporary control group of people with no evidence of SARS-CoV-2 infection was constructed from those who had encountered the VHA in 2019 (*n* = 6,241,346). Of those who were still alive by 1 March 2020 (*n* = 5,960,737), 5,806,977 participants were not in the COVID-19 group and were selected into the contemporary control group. To ensure that this contemporary control group had a similar follow-up time as the COVID-19 group, we randomly assigned *T*_0_ in the contemporary control group based on the distribution of *T*_0_ in the COVID-19 group so that the proportion of people enrolled on a certain date would be the same in both the contemporary and COVID-19 groups. Of 5,658,938 participants alive at the assigned *T*_0_, 5,637,647 participants in the contemporary control group were alive 30 d after *T*_0_. In the COVID-19 and contemporary control groups, 31 October 2021 was the end of follow-up.

To examine the associations between COVID-19 and cardiovascular outcomes compared to those who did not experience the pandemic, a historical control group was constructed from 6,461,205 participants who used the VHA in 2017. Of the 6,150,594 participants who were alive on 1 March 2018, 6,008,499 participants did not enroll into the COVID-19 group and were further selected into the historical control group. To ensure that this historical control group had a similar follow-up time as the COVID-19 group, we randomly assigned *T*_0_ in the historical control group with a similar distribution as *T*_0_ minus 2 years (730 d) in the COVID-19 group. Of 5,875,818 historical control participants alive at assigned *T*_0_, 5,859,411 were alive 30 d after *T*_0_. In the historical control group, end of follow-up was set as 31 October 2019.

### Data sources

Electronic health records from the VA Corporate Data Warehouse (CDW) were used in this study. Demographic information was collected from the CDW Patient domain. The CDW Outpatient Encounters domain provided clinical information pertaining to outpatient encounters, whereas the CDW Inpatient Encounters domain provided clinical information during hospitalization. Medication information was obtained from the CDW Outpatient Pharmacy and CDW Bar Code Medication Administration domains. The CDW Laboratory Results domain provided laboratory test information, and the COVID-19 Shared Data Resource provided information on COVID-19. Additionally, the Area Deprivation index (ADI), which is a composite measure of income, education, employment and housing, was used as a summary measure of contextual disadvantage at participants’ residential locations^22^.

### Pre-specified outcomes

The pre-specified outcomes were selected based on our previous work on the systematic characterization of Long COVID^1,23^. Incident cardiovascular outcomes in the post-acute phase of COVID-19 were assessed in the follow-up period between 30 d after *T*_0_ until the end of follow-up in those without history of the outcome in the year before *T*_0_. Each cardiovascular outcome was defined based on validated diagnostic codes. We also aggregated individual outcomes in a related category of composite outcome (for example, stroke and TIA were aggregated to cerebrovascular disease). We also specified two additional composite outcomes: (1) MACE was a composite outcome of all-cause mortality, myocardial infarction and stroke; and (2) the composite of any cardiovascular outcome was defined as the first incident occurrence of any of the cardiovascular outcomes investigated in this study.

### Covariates

To adjust for the difference in baseline characteristics between groups, we considered both pre-defined and algorithmically selected high-dimensional covariates assessed within 1 year before *T*_0_. Pre-defined variables were selected based on prior knowledge^1,7,24,25^. The pre-defined covariates included age, race (White, Black and Other), sex, ADI, body mass index, smoking status (current, former and never) and healthcare use parameters, including the use number of outpatient and inpatient encounters and use of long-term care. We additionally specified several comorbidities as pre-defined variables, including cancer, chronic kidney disease, chronic lung disease, dementia, diabetes, dysautonomia, hyperlipidemia and hypertension. Additionally, we adjusted for estimated glomerular filtration rate and systolic and diastolic blood pressure. Missing values were accounted for by conditional mean imputation based on value within the group^26^. Continuous variables were transformed into restricted cubic spline functions to account for potential non-linear relationships.

In addition to pre-defined covariates, we further algorithmically selected additional potential confounders from data domains, including diagnoses, medications and laboratory tests^27^. To accomplish this, we gathered all patient encounter, prescription and laboratory data and classified the information into 540 diagnostic categories, 543 medication classes and 62 laboratory test abnormalities. For the diagnoses, medications and laboratory abnormalities that occurred in at least 100 participants within each group, univariate relative risk between the variable and exposure was calculated, and the top 100 variables with the strongest relative risk were selected^28^. The process of algorithmically selecting the high-dimensional covariates was independently conducted for each outcome-specific cohort in each comparison (for example, the COVID-19 versus contemporary control analyses to examine incident heart failure and the COVID-19 versus historical control analyses to examine incident heart failure).

All pre-defined and algorithmically selected covariates were used in the models.

### Statistical analyses

Baseline characteristics of the COVID-19 and contemporary and historical control groups, along with standardized mean difference between groups, were described.

We then estimated the risks, burdens and excess burdens of incident cardiovascular outcomes for COVID-19 compared to the contemporary control group and, separately, compared to the historical control group, after adjusting for differences in baseline characteristics through inverse probability weighting. To estimate the risk of each incident cardiovascular outcome, we built a subcohort of participants without a history of the outcome being examined (that is, the risk of incident heart failure was estimated within a subcohort of participants without history of heart failure in the year before enrollment). In each subcohort, a propensity score for each individual was estimated as the probability of belonging to the VHA users group in 2019 (target population) based on both pre-defined and algorithmically selected high-dimensional variables. This propensity score was then used to calculate the inverse probability weight as the probability of belonging in the target population divided by 1 − the probability of being in the target population. Covariate balance after application of weights was assessed by standardized mean differences.

HRs of incident cardiovascular outcomes between the COVID-19 and contemporary cohorts and the COVID-19 and historical cohorts were estimated from cause-specific hazard models where death was considered as a competing risk, and the inverse probability weights were applied. Burden per 1,000 participants at 12 months of follow-up and the excess burden based on the differences between COVID-19 and control groups were estimated.

We conducted analyses in subgroups by age, race, sex, obesity, smoking, hypertension, diabetes, chronic kidney disease, hyperlipidemia and cardiovascular disease. And, separately, we undertook analyses in a cohort without history of any cardiovascular outcomes before cohort enrollment.

We then developed causal difference-in-differences analyses to estimate the adjusted incident rate ratios of all cardiovascular outcomes in the pre-COVID-19 and post-COVID-19 exposure period relative to both contemporary and historical controls^29–32^. To enhance the interpretability of difference-in-difference analyses, the pre-exposure period was defined as with same follow-up time as the post-exposure period, and the incident rate ratio for the pre-exposure period was examined within those without history of the outcome within 1 year before the period. Incident rate ratios for all groups in the pre-exposure and post- exposure periods were weighted toward the common target population (VHA users in 2019) based on pre-exposure characteristics. The adjusted incident rate ratios in the pre-exposure and post-exposure periods were then compared. Difference-in-differences analyses were also conducted in mutually exclusive groups according to care setting of the acute phase of the disease. We also evaluated the associations between COVID-19 and risks of post-acute cardiovascular sequelae in mutually exclusive groups according to care setting of the acute phase of the disease (that is, whether people were non-hospitalized, hospitalized or admitted into the intensive care unit during the first 30 d of infection). Inverse probability weights were estimated for each care setting group using the approach outlined in the previous paragraph. Cause-specific hazard models with inverse probability weighting were then applied, and HRs, burdens and excess burdens were reported.

We conducted multiple sensitivity analyses to test the robustness of our study results. (1) To capture additional potential confounders, we expanded our inclusion of high-dimensional variables from the top 100 to the top 300 when constructing the inverse probability weight. (2) We then modified our adjustment strategy by using only pre-defined variables when constructing the inverse probability weight (not including the 100 high-dimensional covariates used in the primary analyses). Finally, (3) we alternatively applied a doubly robust approach, where both covariates and the inverse probability weights were applied to the survival models, to estimate the associations^33^.

COVID-19 is associated with an increased risk of fatigue in the post-acute phase of the disease, which is generally considered as a signature post-acute sequela^34^. To test whether our approach would reproduce known associations, we, therefore, examined the association between COVID-19 and fatigue as a positive outcome control. Reproducing this known association (using our data, cohort design and analytic strategy) would provide some measure of assurance that our approach yields result consistent with a priori expectations.

We also subjected our approach to the application of a battery of negative-outcome controls where no prior knowledge supports the existence of a causal association between the exposure and the risks of negative-outcome controls^35^. The negative-outcome controls included hypertrichosis, melanoma in situ, sickle cell trait, perforation of the tympanic membrane, malignant neoplasm of the tongue, B cell lymphoma and Hodgkin’s lymphoma. We also developed and tested a pair of negative-exposure controls (defined as exposure to influenza vaccine in odd-numbered or even-numbered calendar days between 1 March 2020 and 15 January 2021). Our pre-test expectation was that there would be no differences in risk of any of the pre-specified cardiovascular outcomes examined in this analysis between those who received influenza vaccine in odd-numbered versus even-numbered calendar days. The successful application of negative controls might reduce concern about the presence of spurious biases related to cohort building, study design, covariate selection, analytic approaches, outcome ascertainment, residual confounding and other sources of latent biases.

Estimation of variance when weightings were applied was accomplished by using robust sandwich variance estimators. In all analyses, a 95% confidence interval that excluded unity was considered evidence of statistical significance. This study was approved by the institutional review board of the VA St. Louis Health Care System (protocol number 1606333), which granted a waiver of informed consent. Analyses were conducted using SAS Enterprise Guide version 8.2 (SAS Institute), and results were visualized using R version 4.04.

### Ethical approval

This research project was reviewed and approved by the institutional review board of the VA St. Louis Health Care System (protocol number 1606333).

### Reporting Summary

Further information on research design is available in the Nature Research Reporting Summary linked to this article.

## Online content

Any methods, additional references, Nature Research reporting summaries, source data, extended data, supplementary information, acknowledgements, peer review information; details of author contributions and competing interests; and statements of data and code availability are available at 10.1038/s41591-022-01689-3.

## Supplementary information

Supplementary Information Supplementary Tables 1–26.

Reporting Summary

## Data Availability

The data that support the findings of this study are available from the US Department of Veterans Affairs. VA data are made freely available to researchers behind the VA firewall with an approved VA study protocol. For more information, visit https://www.virec.research.va.gov or contact the VA Information Resource Center at VIReC@va.gov.

## Code availability

SAS codes are available at https://github.com/yxie618/longCVD and 10.5281/zenodo.5799457.
