# Supplementary material for: Long-term cardiovascular outcomes of COVID-19
Source: Nat Med. 2022 Feb 7;28(3):583–90. doi: 10.1038/s41591-022-01689-3 (PMC8938267; doi:10.1038/s41591-022-01689-3)
Supplement: Supplementary file 1 — Supplementary Tables 1–26. [file 41591_2022_1689_MOESM1_ESM.pdf]

nature portfolio

https://doi.org/10.1038/s41591-022-01689-3

Supplementary information

Long-term cardiovascular outcomes of COVID-19

In the format provided by the authors and unedited

Long-term Cardiovascular Outcomes of COVID-19

Table of Contents	Page
Supplementary Table 1. Demographic and health characteristics of COVID-19, contemporary, and historical cohorts before weighting	3-4
Supplementary Table 2. Demographic and health characteristics of COVID-19, contemporary, and historical cohorts after weighting	5-6
Supplementary Table 3. Risks and 12-month burdens of incident post-acute COVID-19 cardiovascular outcomes compared to contemporary control	7-8
Supplementary Table 4. Subgroup analyses of the risks of incident post-acute COVID-19 composite cardiovascular outcomes compared to contemporary control	9-10
Supplementary Table 5. Risks and 12-month burdens of incident post-acute COVID-19 cardiovascular outcomes in participants without any history of cardiovascular outcomes prior to COVID-19 exposure compared to contemporary control	11-12
Supplementary Table 6. Demographic and health characteristics of the COVID-19 and contemporary cohorts by care setting of the acute infection before weighting	13-14
Supplementary Table 7. Demographic and health characteristics of the COVID-19 and contemporary cohorts by care setting of the acute infection after weighting	15-16
Supplementary Table 8. Risks and 12-month burdens of incident post-acute COVID-19 cardiovascular outcomes by care setting of the acute infection compared to contemporary control	17-19
Supplementary Table 9. Risks and 12-month burdens of incident post-acute COVID-19 cardiovascular outcomes compared to historical control	20-21
Supplementary Table 10. Subgroup analyses of the risks of incident post-acute COVID-19 composite cardiovascular outcomes compared to historical control	22-23
Supplementary Table 11. Risks and 12-month burdens of incident post-acute COVID-19 cardiovascular outcomes in participants without any history of cardiovascular outcomes prior to COVID-19 exposure compared to historical control	24-25
Supplementary Table 12. Demographic and health characteristics of the COVID-19 and historical cohorts by care setting of the acute infection before weighting	26-27
Supplementary Table 13. Demographic and health characteristics of the COVID-19 and historical cohorts by care setting of the acute infection after weighting	28-29
Supplementary Table 14. Risks and 12-month burdens of incident post-acute COVID-19 cardiovascular outcomes by care setting of the acute infection compared to historical control	30-32

Supplementary Table 15. Difference-in-differences analyses of incident rate ratios of cardiovascular outcomes before and after exposure to COVID-19 compared to contemporary control	33-34
Supplementary Table 16. Difference-in-differences analyses of incident rate ratios of cardiovascular outcomes before and after exposure to COVID-19 by care setting of the acute infection compared to contemporary control	35-37
Supplementary Table 17. Difference-in-differences analyses of incident rate ratios of cardiovascular outcomes before and after exposure to COVID-19 compared to historical control	38-39
Supplementary Table 18. Difference-in-differences analyses of incident rate ratios of cardiovascular outcomes before and after exposure to COVID-19 by care setting of the acute infection compared to historical control	40-42
Supplementary Table 19. Sensitivity analysis for MACE and any cardiovascular outcome compared to contemporary and historical controls	43
Supplementary Table 20. Sensitivity analyses for MACE and any cardiovascular outcome by care setting of the acute infection compared to contemporary and historical controls	44
Supplementary Table 21. Risks of incident post-acute COVID-19 myocarditis and pericarditis compared to contemporary control in analyses where cohort participants are censored at time of vaccination	45
Supplementary Table 22. Risks of incident post-acute COVID-19 myocarditis and pericarditis compared to contemporary control in analyses adjusting for time varying COVID-19 vaccination	46
Supplementary Table 23. Risks of incident post-acute COVID-19 myocarditis and pericarditis compared to historical control in analyses where cohort participants are censored at time of vaccination	47
Supplementary Table 24. Risks of incident post-acute COVID-19 myocarditis and pericarditis compared to historical control in analyses adjusting for time varying COVID-19 vaccination	48
Supplementary Table 25. Positive and negative outcome controls	49
Supplementary Table 26. Negative exposure control: risks and 12-month burdens of cardiovascular outcomes of those vaccinated for influenza on even-numbered days compared to those vaccinated on odd-numbered days	50-51

Supplementary Table 1. Demographic and health characteristics of COVID-19, contemporary, and historical controls before weighting

Baseline Characteristics	COVID-19 (N=153,760)	Contemporary control (N=5,637,647)	Historical control (N=5,859,411)	Absolute standardized difference – COVID-19 and Contemporary control	Absolute standardized difference – COVID-19 and Historical control
**Age, mean (std), yr**	61.42 (15.64)	63.46 (16.23)	62.90 (16.48)	0.13	0.09
**Race, no. (%)**					
White	108,978 (70.88)	4,328,756 (76.78)	4,532,542 (77.35)	0.13	0.15
Black	37,113 (24.14)	1,042,153 (18.49)	1,051,656 (17.95)	0.14	0.15
Other	7,669 (4.99)	266,738 (4.73)	275,213 (4.70)	0.01	0.01
**Sex, no. (%)**					
Male	136,912 (89.04)	5,091,519 (90.31)	5,308,854 (90.60)	0.04	0.05
Female	16,848 (10.96)	546,128 (9.69)	550,557 (9.40)	0.04	0.05
**BMI category, no. (%), kg/m^2**					
BMI ≤ 25	22,192 (14.43)	1,099,292 (19.50)	1,162,294 (19.84)	0.14	0.14
25 < BMI ≤ 30	48,786 (31.73)	2,158,693 (38.29)	2,296,904 (39.20)	0.14	0.16
BMI > 30	82,782 (53.84)	2,379,662 (42.21)	2,400,213 (40.96)	0.23	0.26
**Smoking status, no. (%)**					
Never	69,127 (44.96)	2,398,958 (42.55)	3,086,138 (52.67)	0.05	0.15
Former	61,084 (39.73)	2,031,298 (36.03)	1,444,066 (24.65)	0.08	0.33
Current	23,549 (15.32)	1,207,391 (21.42)	1,329,207 (22.69)	0.16	0.19
**Area Deprivation Index[*], mean (std)**	55.32 (18.59)	54.65 (19.05)	54.62 (19.13)	0.04	0.04
**Clinical Characteristics**					
**Outpatient encounter[†], no. (%)**					
Zero	4,156 (2.70)	666,096 (11.82)	654,793 (11.18)	0.36	0.34
One	26,483 (17.22)	1,784,211 (31.65)	1,775,335 (30.30)	0.34	0.31
Two or more	123,121 (80.07)	3,187,340 (56.54)	3,429,283 (58.53)	0.52	0.48
**Long-term care, no. (%)**	6,107 (3.97)	33,611 (0.60)	47,384 (0.81)	0.23	0.21

**eGFR, mean (std), ml/min/1.73m^2**	77.74 (22.50)	78.59 (20.15)	79.31 (19.95)	0.04	0.07
**Systolic blood pressure, mean (std), mmHg**	132.65 (11.74)	132.65 (12.29)	132.63 (12.63)	0.00	0.00
**Diastolic blood pressure, mean (std), mmHg**	78.32 (7.42)	77.77 (7.54)	77.53 (7.91)	0.07	0.10
**Cancer, no. (%)**	12,524 (8.15)	344,668 (6.11)	345,608 (5.90)	0.08	0.09
**Chronic kidney disease, no. (%)**	30,009 (19.52)	940,048 (16.67)	902,029 (15.39)	0.07	0.11
**Chronic lung disease, no. (%)**	23,469 (15.26)	609,531 (10.81)	652,107 (11.13)	0.13	0.12
**Dementia, no. (%)**	9,497 (6.18)	172,128 (3.05)	191,866 (3.27)	0.15	0.14
**Diabetes mellitus type 2, no. (%)**	49,407 (32.13)	1,272,500 (22.57)	1,330,448 (22.71)	0.22	0.21
**Dysautonomia, no. (%)**	2,812 (1.83)	67,704 (1.20)	72,975 (1.25)	0.05	0.05
**Hyperlipidemia, no. (%)**	52,227 (33.97)	1,435,909 (25.47)	1,810,965 (30.91)	0.19	0.07
**Hypertension, no. (%)**	41,060 (26.70)	1,484,884 (26.34)	1,540,087 (26.28)	0.01	0.01

*. Area Deprivation Index is a measure of socioeconomic disadvantage, with a range from low to high disadvantage of 0 to 100.
†. Data collected within one year of cohort enrollment
std, standard deviation; BMI, body mass index; eGFR, estimated glomerular filtration rate.

Supplementary Table 2. Demographic and health characteristics of COVID-19, contemporary, and historical controls after weighting

Baseline Characteristics	COVID-19 (N=153,760)	Contemporary control (N=5,637,647)	Historical control (N=5,859,411)	Absolute standardized difference - COVID-19 and Contemporary control	Absolute standardized difference - COVID-19 and Historical control
**Age, mean (std), yr**	63.52 (16.10)	63.41 (16.22)	63.35 (16.30)	0.01	0.01
**Race, no. (%)**					
White	116,971 (76.07)	4,320,467 (76.64)	4,488,485 (76.60)	0.01	0.01
Black	29,473 (19.17)	1,050,125 (18.63)	1,091,960 (18.64)	0.01	0.01
Other	7,316 (4.76)	267,112 (4.74)	278,967 (4.76)	0.00	0.00
**Sex, no. (%)**					
Male	137,617 (89.50)	5,089,442 (90.28)	5,288,411 (90.26)	0.03	0.03
Female	16,143 (10.50)	548,205 (9.72)	571,000 (9.75)	0.03	0.03
**BMI category, no. (%), kg/m^2**					
BMI ≤ 25	30,079 (19.56)	1,092,801 (19.38)	1,138,601 (19.43)	0.00	0.00
25 < BMI ≤ 30	54,788 (35.63)	2,149,127 (38.12)	2,244,506 (38.31)	0.05	0.06
BMI > 30	68,895 (44.81)	2,395,718 (42.50)	2,476,304 (42.26)	0.05	0.05
**Smoking status, no. (%)**					
Never	61,979 (40.31)	2,401,638 (42.60)	2,493,062 (42.55)	0.05	0.05
Former	57,296 (37.26)	2,036,938 (36.13)	2,120,814 (36.20)	0.02	0.02
Current	34,485 (22.43)	1,199,128 (21.27)	1,245,594 (21.26)	0.03	0.03
**Area Deprivation Index[*], mean (std)**	55.11 (18.96)	54.66 (19.04)	54.67 (19.02)	0.02	0.02
**Clinical Characteristics**					
**Outpatient encounter[†], no. (%)**					
Zero	15,530 (10.10)	652,389 (11.57)	669,262 (11.42)	0.05	0.04
One	42,492 (27.64)	1,761,821 (31.25)	1,837,511 (31.36)	0.08	0.08
Two or more	95,739 (62.27)	3,223,437 (57.18)	3,352,579 (57.22)	0.10	0.10
**Long-term care, no. (%)**	1,719 (1.12)	39,125 (0.69)	41,074 (0.70)	0.04	0.04

**eGFR, mean (std), ml/min/1.73m^2**	78.31 (20.78)	78.57 (20.21)	78.63 (20.31)	0.01	0.02
**Systolic blood pressure, mean (std), mmHg**	132.47 (12.31)	132.65 (12.28)	132.55 (12.36)	0.01	0.01
**Diastolic blood pressure, mean (std), mmHg**	77.65 (7.55)	77.79 (7.54)	77.78 (7.58)	0.02	0.02
**Cancer, no. (%)**	10,709 (6.97)	348,463 (6.18)	337,209 (5.76)	0.03	0.05
**Chronic kidney disease, no. (%)**	27,326 (17.77)	944,137 (16.75)	980,045 (16.73)	0.03	0.03
**Chronic lung disease, no. (%)**	20,218 (13.15)	617,097 (10.95)	641,664 (10.95)	0.07	0.07
**Dementia, no. (%)**	6,127 (3.99)	176,853 (3.14)	184,044 (3.14)	0.05	0.05
**Diabetes mellitus type 2, no. (%)**	37,504 (24.39)	1,287,018 (22.83)	1,332,196 (22.74)	0.04	0.04
**Dysautonomia, no. (%)**	2,216 (1.44)	68,723 (1.22)	70,957 (1.21)	0.02	0.02
**Hyperlipidemia, no. (%)**	41,838 (27.21)	1,448,706 (25.70)	1,507,041 (25.72)	0.03	0.03
**Hypertension, no. (%)**	40,522 (26.35)	1,485,520 (26.35)	1,538,388 (26.26)	0.00	0.00

*. Area Deprivation Index is a measure of socioeconomic disadvantage, with a range from low to high disadvantage of 0 to 100.
†. Data collected within one year before cohort enrollment
std, standard deviation; BMI, body mass index; eGFR, estimated glomerular filtration rate.

Supplementary Table 3. Risks and 12-month burdens of incident post-acute COVID-19 cardiovascular outcomes compared to contemporary control

Outcome*	Hazard Ratio (95% CI)† COVID-19 vs Contemporary control	COVID-19 burden per 1000 persons at 12 months (95% CI)†	Contemporary control burden per 1000 persons at 12 months (95% CI)†	Absolute burden difference per 1000 persons at 12 months (95% CI)†
**Cerebrovascular**	1.53 (1.45, 1.61)	15.95 (15.13, 16.83)	10.48 (10.39, 10.56)	5.48 (4.65, 6.35)
Stroke	1.52 (1.43, 1.62)	11.80 (11.08, 12.56)	7.77 (7.69, 7.84)	4.03 (3.32, 4.79)
TIA	1.49 (1.37, 1.62)	5.62 (5.16, 6.12)	3.78 (3.73, 3.83)	1.84 (1.38, 2.34)
**Dysrhythmia**	1.69 (1.64, 1.75)	49.37 (47.82, 50.97)	29.51 (29.37, 29.65)	19.86 (18.31, 21.46)
Atrial fibrillation	1.71 (1.64, 1.79)	25.99 (24.86, 27.16)	15.25 (15.15, 15.35)	10.74 (9.61, 11.91)
Sinus tachycardia	1.84 (1.74, 1.95)	12.68 (11.97, 13.43)	6.90 (6.83, 6.97)	5.78 (5.07, 6.53)
Sinus bradycardia	1.53 (1.45, 1.62)	13.35 (12.63, 14.11)	8.73 (8.65, 8.81)	4.62 (3.90, 5.38)
Ventricular arrhythmia	1.84 (1.72, 1.98)	9.17 (8.55, 9.83)	4.98 (4.93, 5.04)	4.18 (3.56, 4.85)
Atrial flutter	1.80 (1.66, 1.96)	6.98 (6.43, 7.57)	3.88 (3.83, 3.93)	3.10 (2.55, 3.69)
**Inflammatory heart disease**	2.02 (1.77, 2.30)	2.44 (2.14, 2.78)	1.21 (1.18, 1.24)	1.23 (0.93, 1.57)
Pericarditis	1.85 (1.61, 2.13)	2.13 (1.85, 2.45)	1.15 (1.12, 1.18)	0.98 (0.70, 1.30)
Myocarditis	5.38 (3.80, 7.59)	0.38 (0.27, 0.53)	0.07 (0.06, 0.08)	0.31 (0.20, 0.46)
**Ischemic heart disease**	1.66 (1.52, 1.80)	18.47 (16.99, 20.07)	11.19 (11.10, 11.28)	7.28 (5.80, 8.88)
Acute coronary disease	1.72 (1.56, 1.90)	12.82 (11.60, 14.16)	7.46 (7.39, 7.54)	5.35 (4.13, 6.70)
Myocardial infarction	1.63 (1.51, 1.75)	7.59 (7.05, 8.17)	4.67 (4.62, 4.73)	2.91 (2.38, 3.49)
Ischemic cardiomyopathy	1.75 (1.44, 2.13)	5.47 (4.50, 6.64)	3.13 (3.08, 3.18)	2.34 (1.37, 3.51)
Angina	1.52 (1.42, 1.64)	7.28 (6.78, 7.81)	4.78 (4.73, 4.84)	2.50 (2.00, 3.03)
**Other cardiac disorders**	1.72 (1.65, 1.79)	30.67 (29.48, 31.90)	17.94 (17.83, 18.05)	12.72 (11.54, 13.96)
Heart failure	1.72 (1.65, 1.80)	27.92 (26.79, 29.10)	16.31 (16.21, 16.42)	11.61 (10.47, 12.78)
Non-ischemic cardiomyopathy	1.62 (1.52, 1.73)	9.32 (8.72, 9.96)	5.75 (5.69, 5.81)	3.56 (2.97, 4.20)
Cardiac arrest	2.45 (2.08, 2.89)	1.20 (1.02, 1.42)	0.49 (0.47, 0.51)	0.71 (0.53, 0.93)
Cardiogenic shock	2.43 (1.86, 3.16)	0.87 (0.66, 1.13)	0.36 (0.34, 0.37)	0.51 (0.31, 0.77)
**Thrombotic disorders**	2.39 (2.27, 2.51)	17.07 (16.25, 17.94)	7.19 (7.12, 7.26)	9.88 (9.05, 10.74)
Pulmonary embolism	2.93 (2.73, 3.15)	8.31 (7.75, 8.92)	2.84 (2.80, 2.89)	5.47 (4.90, 6.08)
Deep vein thrombosis	2.09 (1.94, 2.24)	8.04 (7.48, 8.65)	3.86 (3.81, 3.91)	4.18 (3.62, 4.79)
Superficial vein thrombosis	1.95 (1.80, 2.12)	5.37 (4.95, 5.82)	2.76 (2.71, 2.80)	2.61 (2.20, 3.07)

**MACE**	1.55 (1.50, 1.60)	67.67 (65.73, 69.67)	44.19 (44.02, 44.36)	23.48 (21.54, 25.48)
**Any cardiovascular outcome**	1.63 (1.59, 1.68)	121.60 (118.52, 124.76)	76.31 (76.08, 76.54)	45.29 (42.22, 48.45)

*. Outcomes were ascertained from day 30 after the initial positive COVID-19 test result until end of follow up
†. Adjustment through inverse probability weighting using predefined and algorithmically selected high-dimensional variables.
CI, confidence interval; TIA, transient ischemic attack; MACE, major adverse cardiac events.

Supplementary Table 4. Subgroup analyses of the risks of incident post-acute COVID-19 composite cardiovascular outcomes compared to contemporary control

Risk factors	Hazard ratio (95% CI)* - COVID-19 vs Contemporary control							
	Cerebro-vascular	Dysrhythmia	Inflammatory heart disease	Ischemic heart disease	Other cardiac disorders	Thrombotic disorders	MACE	Any cardiovascular outcome
**Age**								
≤65	1.44 (1.29, 1.60)	1.68 (1.59, 1.77)	1.86 (1.40, 2.45)	1.66 (1.45, 1.91)	1.55 (1.43, 1.69)	2.18 (1.99, 2.37)	1.32 (1.23, 1.42)	1.58 (1.52, 1.64)
>65	1.53 (1.44, 1.63)	1.68 (1.58, 1.79)	2.07 (1.79, 2.40)	1.60 (1.51, 1.71)	1.77 (1.69, 1.86)	2.50 (2.35, 2.66)	1.60 (1.53, 1.66)	1.68 (1.61, 1.75)
**Race**								
White	1.56 (1.46, 1.67)	1.68 (1.62, 1.75)	2.18 (1.86, 2.55)	1.56 (1.47, 1.65)	1.71 (1.63, 1.79)	2.29 (2.16, 2.44)	1.54 (1.49, 1.60)	1.64 (1.59, 1.69)
Black	1.50 (1.35, 1.66)	1.67 (1.57, 1.79)	1.69 (1.36, 2.12)	2.00 (1.34, 2.99)	1.79 (1.65, 1.93)	2.41 (2.2, 2.63)	1.67 (1.56, 1.79)	1.68 (1.60, 1.76)
**Sex**								
Male	1.51 (1.43, 1.60)	1.71 (1.65, 1.77)	2.03 (1.77, 2.34)	1.63 (1.51, 1.75)	1.75 (1.68, 1.83)	2.41 (2.29, 2.53)	1.50 (1.46, 1.55)	1.66 (1.61, 1.71)
Female	1.15 (0.91, 1.45)	1.48 (1.32, 1.66)	1.87 (1.31, 2.67)	1.38 (1.11, 1.72)	1.34 (1.10, 1.64)	1.63 (1.32, 2.02)	1.39 (1.15, 1.68)	1.46 (1.32, 1.61)
**Obesity**†								
No	1.56 (1.45, 1.68)	1.78 (1.69, 1.87)	2.25 (1.85, 2.74)	1.70 (1.52, 1.90)	1.91 (1.80, 2.03)	2.51 (2.34, 2.70)	1.56 (1.5, 1.62)	1.73 (1.67, 1.80)
Yes	1.55 (1.42, 1.68)	1.54 (1.48, 1.61)	1.76 (1.49, 2.08)	1.50 (1.4, 1.61)	1.57 (1.49, 1.65)	2.21 (2.05, 2.38)	1.44 (1.38, 1.51)	1.54 (1.49, 1.59)
**Smoking**								
No	1.43 (1.36, 1.50)	1.51 (1.47, 1.56)	1.86 (1.65, 2.11)	1.57 (1.50, 1.65)	1.56 (1.51, 1.62)	2.05 (1.96, 2.15)	1.48 (1.43, 1.52)	1.55 (1.52, 1.58)
Yes	1.61 (1.42, 1.83)	1.73 (1.59, 1.89)	1.97 (1.41, 2.74)	1.61 (1.39, 1.86)	1.58 (1.42, 1.75)	2.09 (1.83, 2.38)	1.51 (1.41, 1.63)	1.61 (1.51, 1.70)

**Hypertension**								
No	1.59 (1.49, 1.71)	1.71 (1.64, 1.78)	2.11 (1.78, 2.49)	1.64 (1.39, 1.93)	1.74 (1.65, 1.83)	2.44 (2.30, 2.59)	1.57 (1.51, 1.63)	1.66 (1.61, 1.72)
Yes	1.49 (1.36, 1.62)	1.58 (1.50, 1.67)	1.98 (1.60, 2.45)	1.76 (1.59, 1.95)	1.71 (1.60, 1.83)	2.22 (2.03, 2.43)	1.54 (1.46, 1.62)	1.57 (1.51, 1.64)
**Diabetes**								
No	1.65 (1.53, 1.77)	1.74 (1.67, 1.81)	1.98 (1.65, 2.39)	1.81 (1.52, 2.15)	1.86 (1.76, 1.97)	2.48 (2.32, 2.65)	1.60 (1.53, 1.66)	1.67 (1.62, 1.73)
Yes	1.40 (1.31, 1.51)	1.51 (1.43, 1.60)	1.87 (1.58, 2.21)	1.44 (1.35, 1.53)	1.51 (1.44, 1.59)	2.14 (1.99, 2.30)	1.42 (1.36, 1.47)	1.52 (1.44, 1.60)
**Chronic kidney disease**‡								
No	1.61 (1.50, 1.72)	1.71 (1.64, 1.79)	2.05 (1.73, 2.43)	1.71 (1.47, 2.00)	1.77 (1.68, 1.86)	2.40 (2.26, 2.55)	1.56 (1.50, 1.63)	1.66 (1.60, 1.71)
Yes	1.43 (1.31, 1.57)	1.54 (1.46, 1.64)	1.87 (1.55, 2.24)	1.54 (1.41, 1.68)	1.63 (1.53, 1.74)	2.29 (2.10, 2.51)	1.48 (1.41, 1.56)	1.55 (1.44, 1.67)
**Hyperlipidemia**								
No	1.66 (1.55, 1.77)	1.68 (1.61, 1.75)	2.16 (1.85, 2.53)	1.69 (1.59, 1.79)	1.86 (1.77, 1.95)	2.48 (2.33, 2.64)	1.64 (1.58, 1.70)	1.69 (1.64, 1.74)
Yes	1.41 (1.29, 1.53)	1.59 (1.49, 1.68)	1.56 (1.29, 1.89)	1.54 (1.30, 1.81)	1.41 (1.32, 1.50)	2.19 (2.02, 2.38)	1.34 (1.27, 1.41)	1.49 (1.42, 1.56)
**Cardiovascular disease**								
No	1.49 (1.40, 1.59)	1.71 (1.62, 1.81)	2.02 (1.69, 2.42)	1.57 (1.48, 1.67)	1.72 (1.63, 1.80)	2.42 (2.28, 2.57)	1.53 (1.48, 1.59)	1.63 (1.59, 1.68)
Yes	1.35 (1.23, 1.48)	1.35 (1.27, 1.43)	1.73 (1.47, 2.05)	1.35 (1.26, 1.45)	1.50 (1.41, 1.60)	1.80 (1.65, 1.97)	1.34 (1.28, 1.40)	NA

*. Adjustment through inverse probability weighting using predefined and algorithmically selected high-dimensional variables.
†. Obesity was defined based on baseline BMI>30kg/m^2
‡. Chronic kidney disease was defined based on baseline outpatient estimated Glomerular Filtration Rate< 60 mL/min/1.73 m^2
CI, confidence interval; MACE, major adverse cardiac events, NA, not applicable

Supplementary Table 5. Risks and 12-month burdens of incident post-acute COVID-19 cardiovascular outcomes in participants without any history of cardiovascular outcomes prior to COVID-19 exposure compared to contemporary control

Outcome*	Hazard Ratio (95% CI)†	COVID-19 burden per 1000 persons at 12 months (95% CI)†	Contemporary control burden per 1000 persons at 12 months (95% CI)†	Absolute burden difference per 1000 persons at 12 months (95% CI)†
	COVID-19 vs Contemporary control			
**Cerebrovascular**	1.49 (1.40, 1.59)	13.45 (12.61, 14.34)	9.03 (8.95, 9.11)	4.42 (3.58, 5.31)
Stroke	1.48 (1.37, 1.59)	9.78 (9.08, 10.55)	6.64 (6.57, 6.71)	3.14 (2.43, 3.90)
TIA	1.46 (1.31, 1.63)	4.49 (4.03, 5.00)	3.07 (3.02, 3.12)	1.42 (0.96, 1.93)
**Dysrhythmia**	1.71 (1.62, 1.81)	43.73 (41.42, 46.17)	25.76 (25.63, 25.90)	17.97 (15.66, 20.41)
Atrial fibrillation	1.68 (1.58, 1.77)	20.21 (19.12, 21.37)	12.11 (12.01, 12.20)	8.11 (7.01, 9.26)
Sinus tachycardia	1.84 (1.72, 1.97)	10.83 (10.12, 11.60)	5.90 (5.83, 5.97)	4.93 (4.21, 5.70)
Sinus bradycardia	1.58 (1.47, 1.69)	11.03 (10.29, 11.83)	7.02 (6.94, 7.09)	4.02 (3.28, 4.81)
Ventricular arrhythmia	2.14 (1.61, 2.84)	6.91 (5.21, 9.16)	3.23 (3.18, 3.28)	3.68 (1.98, 5.93)
Atrial flutter	1.93 (1.71, 2.17)	4.46 (3.97, 5.03)	2.32 (2.28, 2.36)	2.15 (1.65, 2.71)
**Inflammatory heart disease**	2.02 (1.69, 2.42)	1.71 (1.43, 2.05)	0.85 (0.82, 0.87)	0.87 (0.58, 1.20)
Pericarditis	1.85 (1.52, 2.25)	1.48 (1.21, 1.79)	0.80 (0.77, 0.82)	0.68 (0.41, 1.00)
Myocarditis	5.74 (3.84, 8.59)	0.30 (0.20, 0.44)	0.05 (0.05, 0.06)	0.25 (0.15, 0.39)
**Ischemic heart disease**	1.57 (1.48, 1.67)	12.92 (12.16, 13.73)	8.26 (8.18, 8.34)	4.66 (3.91, 5.47)
Acute coronary disease	1.62 (1.49, 1.76)	7.92 (7.30, 8.60)	4.90 (4.84, 4.96)	3.02 (2.40, 3.69)
Myocardial infarction	1.51 (1.37, 1.67)	5.12 (4.63, 5.67)	3.39 (3.34, 3.44)	1.73 (1.24, 2.28)
Ischemic cardiomyopathy	1.67 (1.42, 1.95)	2.39 (2.04, 2.79)	1.43 (1.40, 1.46)	0.95 (0.60, 1.36)
Angina	1.51 (1.39, 1.64)	5.82 (5.36, 6.32)	3.86 (3.81, 3.91)	1.96 (1.50, 2.46)
**Other cardiac disorders**	1.72 (1.63, 1.80)	24.45 (23.26, 25.71)	14.33 (14.22, 14.43)	10.12 (8.93, 11.38)
Heart failure	1.74 (1.65, 1.84)	21.41 (20.27, 22.61)	12.35 (12.25, 12.45)	9.06 (7.92, 10.26)
Non-ischemic cardiomyopathy	1.60 (1.44, 1.77)	5.21 (4.71, 5.78)	3.26 (3.21, 3.31)	1.95 (1.44, 2.51)
Cardiac arrest	2.73 (1.86, 4.01)	0.80 (0.54, 1.17)	0.29 (0.28, 0.31)	0.51 (0.25, 0.88)
Cardiogenic shock	2.00 (1.35, 2.96)	0.30 (0.20, 0.44)	0.15 (0.14, 0.16)	0.15 (0.05, 0.29)
**Thrombotic disorders**	2.42 (2.28, 2.57)	14.97 (14.11, 15.87)	6.21 (6.14, 6.27)	8.76 (7.91, 9.66)
Pulmonary embolism	3.11 (2.86, 3.39)	7.24 (6.65, 7.87)	2.33 (2.29, 2.37)	4.91 (4.32, 5.54)
Deep vein thrombosis	2.06 (1.89, 2.25)	6.60 (6.04, 7.21)	3.21 (3.16, 3.26)	3.39 (2.83, 4.01)
Superficial vein thrombosis	2.13 (1.91, 2.37)	3.97 (3.57, 4.42)	1.87 (1.83, 1.91)	2.11 (1.70, 2.55)
**MACE**	1.53 (1.48, 1.59)	57.25 (55.19, 59.39)	37.72 (37.55, 37.88)	19.54 (17.47, 21.68)

**Any cardiovascular outcome**	1.63 (1.59, 1.68)	121.60 (118.52, 124.76)	76.31 (76.08, 76.54)	45.29 (42.22, 48.45)

*. Outcomes were ascertained from day 30 after the initial positive COVID-19 test result until end of follow up
†. Adjustment through inverse probability weighting using predefined and algorithmically selected high-dimensional variables.
CI, confidence interval; TIA, transient ischemic attack; MACE, major adverse cardiac events.

Supplementary Table 6. Demographic and health characteristics of the COVID-19 and contemporary cohorts by care setting of the acute infection before weighting

Baseline Characteristics	Non-hospitalized (N=131,612)	Hospitalized (N=16,760)	Admitted to intensive care (N=5,388)	Contemporary control (N=5,637,647)	Absolute standardized difference - Non-hospitalized and Contemporary control	Absolute standardized difference - Hospitalized and Contemporary control	Absolute standardized difference - Admitted to intensive care and Contemporary control
**Age, mean (std), yr**	60.23 (15.72)	68.38 (13.38)	68.95 (12.03)	63.46 (16.23)	0.20	0.33	0.38
**Race, no. (%)**							
White	95,035 (72.21)	10,585 (63.16)	3,358 (62.32)	4,328,756 (76.78)	0.11	0.30	0.32
Black	30,166 (22.92)	5,228 (31.19)	1,719 (31.90)	1,042,153 (18.49)	0.11	0.30	0.31
Other	6,411 (4.87)	947 (5.65)	311 (5.77)	266,738 (4.73)	0.01	0.04	0.05
**Sex, no. (%)**							
Male	116,032 (88.16)	15,774 (94.12)	5,106 (94.77)	5,091,519 (90.31)	0.07	0.14	0.17
Female	15,580 (11.84)	986 (5.88)	282 (5.23)	546,128 (9.69)	0.07	0.14	0.17
**BMI category, no. (%), kg/m^2**							
BMI ≤ 25	17,581 (13.36)	3,571 (21.31)	1,040 (19.30)	1,099,292 (19.50)	0.17	0.04	0.00
25 < BMI ≤ 30	42,041 (31.94)	5,134 (30.63)	1,611 (29.90)	2,158,693 (38.29)	0.13	0.16	0.18
BMI > 30	71,990 (54.70)	8,055 (48.06)	2,737 (50.80)	2,379,662 (42.21)	0.25	0.12	0.17
**Smoking status, no. (%)**							
Never	59,811 (45.45)	7,140 (42.60)	2,176 (40.39)	2,398,958 (42.55)	0.06	0.00	0.04
Former	51,871 (39.41)	6,844 (40.84)	2,369 (43.97)	2,031,298 (36.03)	0.07	0.10	0.16
Current	19,930 (15.14)	2,776 (16.56)	843 (15.65)	1,207,391 (21.42)	0.16	0.12	0.15
**Area Deprivation Index*, mean (std)**	55.63 (18.55)	53.13 (18.70)	54.62 (18.63)	54.65 (19.05)	0.05	0.08	0.00
**Clinical Characteristics**							
**Outpatient encounter†, no. (%)**							
Zero	3,909 (2.97)	185 (1.10)	62 (1.15)	666,096 (11.82)	0.34	0.45	0.44
One	24,764 (18.82)	1,311 (7.82)	408 (7.57)	1,784,211 (31.65)	0.30	0.63	0.64

Two or more	102,939 (78.21)	15,264 (91.07)	4,918 (91.28)	3,187,340 (56.54)	0.48	0.85	0.86
**Long-term care, no. (%)**	3,884 (2.95)	1,726 (10.30)	497 (9.22)	33611 (0.60)	0.18	0.44	0.41
**eGFR, mean (std), ml/min/1.73m^2**	79.47 (21.31)	68.26 (26.13)	65.07 (26.79)	78.59 (20.15)	0.04	0.44	0.57
**Systolic blood pressure, mean (std), mmHg**	132.35 (11.69)	134.38 (11.83)	134.57 (12.14)	132.65 (12.29)	0.02	0.14	0.16
**Diastolic blood pressure, mean (std), mmHg**	78.54 (7.37)	77.06 (7.46)	76.99 (7.73)	77.77 (7.54)	0.10	0.10	0.10
**Cancer, no. (%)**	9,315 (7.08)	2,411 (14.39)	798 (14.81)	344,668 (6.11)	0.04	0.28	0.29
**Chronic kidney disease, no. (%)**	21,928 (16.66)	5,946 (35.48)	2,135 (39.63)	940,048 (16.67)	0.00	0.44	0.53
**Chronic lung disease, no. (%)**	17,740 (13.48)	4,208 (25.11)	1,521 (28.23)	609,531 (10.81)	0.08	0.38	0.45
**Dementia, no. (%)**	6,344 (4.82)	2,472 (14.75)	681 (12.64)	172,128 (3.05)	0.09	0.42	0.36
**Diabetes mellitus type 2, no. (%)**	39,029 (29.66)	7,689 (45.88)	2,689 (49.91)	1,272,500 (22.57)	0.16	0.51	0.59
**Dysautonomia, no. (%)**	2,120 (1.61)	525 (3.13)	167 (3.10)	67,704 (1.20)	0.03	0.13	0.13
**Hyperlipidemia, no. (%)**	44,815 (34.05)	5,535 (33.03)	1,877 (34.84)	1,435,909 (25.47)	0.19	0.17	0.21
**Hypertension, no. (%)**	34,060 (25.88)	5,290 (31.56)	1,710 (31.74)	1,484,884 (26.34)	0.01	0.12	0.12

*. Area Deprivation Index is a measure of socioeconomic disadvantage, with a range from low to high disadvantage of 0 to 100.
†. Data collected within one year before cohort enrollment
std, standard deviation; BMI, body mass index; eGFR, estimated glomerular filtration rate.

Supplementary Table 7. Demographic and health characteristics of the COVID-19 and contemporary cohorts by care setting of the acute infection after weighting

Baseline Characteristics	Non-hospitalized (N=131,612)	Hospitalized (N=16,760)	Admitted to intensive care (N=5,388)	Contemporary control (N=5,637,647)	Absolute standardized difference - Non-hospitalized and Contemporary control	Absolute standardized difference - Hospitalized and Contemporary control	Absolute standardized difference - Admitted to intensive care and Contemporary control
**Age, mean (std), yr**	63.06 (16.29)	63.21 (15.88)	63.46 (16.07)	63.41 (16.22)	0.02	0.01	0.00
**Race, no. (%)**							
White	99,846 (75.86)	12,406 (74.02)	3,951 (73.33)	4,320,129 (76.63)	0.02	0.06	0.08
Black	25,339 (19.25)	3,512 (20.96)	1,073 (19.92)	1,050,406 (18.63)	0.02	0.06	0.03
Other	6,427 (4.88)	842 (5.02)	364 (6.75)	267,112 (4.74)	0.01	0.01	0.09
**Sex, no. (%)**							
Male	118,151 (89.77)	15,285 (91.20)	4,982 (92.46)	5,089,555 (90.28)	0.02	0.03	0.08
Female	13,461 (10.23)	1475 (8.80)	406 (7.54)	548,092 (9.72)	0.02	0.03	0.08
**BMI category, no. (%), kg/m^2**							
BMI ≤ 25	25,091 (19.06)	3,299 (19.68)	965 (17.91)	1,092,407 (19.38)	0.01	0.01	0.04
25 < BMI ≤ 30	47,358 (35.98)	5,713 (34.09)	1,917 (35.57)	2,149,297 (38.12)	0.04	0.08	0.05
BMI > 30	59,164 (44.95)	7,748 (46.23)	2,506 (46.52)	2,395,944 (42.50)	0.05	0.08	0.08
**Smoking status, no. (%)**							
Never	54,307 (41.26)	6,714 (40.06)	2,236 (41.50)	2,402,258 (42.61)	0.03	0.05	0.02
Former	48,812 (37.09)	5,972 (35.63)	1,963 (36.43)	2,036,825 (36.13)	0.02	0.01	0.01
Current	28,493 (21.65)	4,073 (24.30)	1,189 (22.07)	1,198,507 (21.26)	0.01	0.07	0.02
**Area Deprivation Index*, mean (std)**	54.95 (18.93)	55.04 (19.30)	55.96 (19.24)	54.66 (19.04)	0.01	0.02	0.07
**Clinical Characteristics**							
**Outpatient encounter†, no. (%)**							
Zero	15,124 (11.49)	1,664 (9.93)	464 (8.62)	652,558 (11.58)	0.00	0.05	0.10
One	40,762 (30.97)	5,617 (33.51)	1,910 (35.45)	1,761,990 (31.25)	0.01	0.05	0.09

Two or more	75,728 (57.54)	9,479 (56.56)	3,014 (55.93)	3,223,099 (57.17)	0.01	0.01	0.03
**Long-term care, no. (%)**	1,191 (0.91)	240 (1.43)	87 (1.61)	38,674 (0.69)	0.02	0.07	0.09
**eGFR, mean (std), ml/min/1.73m^2**	78.55 (20.59)	77.93 (21.54)	77.72 (21.68)	78.57 (20.21)	0.00	0.03	0.04
**Systolic blood pressure, mean (std), mmHg**	132.58 (12.42)	133.18 (12.41)	134.03 (12.22)	132.65 (12.28)	0.01	0.04	0.11
**Diastolic blood pressure, mean (std), mmHg**	77.82 (7.62)	78.22 (7.61)	78.30 (7.71)	77.79 (7.53)	0.00	0.06	0.07
**Cancer, no. (%)**	8,092 (6.15)	1,193 (7.12)	370 (6.87)	348,350 (6.18)	0.00	0.04	0.03
**Chronic kidney disease, no. (%)**	22,753 (17.29)	3,175 (18.94)	1,037 (19.24)	944,249 (16.75)	0.01	0.06	0.06
**Chronic lung disease, no. (%)**	14,908 (11.33)	2,071 (12.36)	639 (11.87)	616,364 (10.93)	0.01	0.04	0.03
**Dementia, no. (%)**	4,445 (3.38)	765 (4.56)	254 (4.72)	176,797 (3.14)	0.01	0.07	0.08
**Diabetes mellitus type 2, no. (%)**	30,642 (23.28)	4,359 (26.01)	1,474 (27.35)	1,286,737 (22.82)	0.01	0.07	0.10
**Dysautonomia, no. (%)**	1,683 (1.28)	226 (1.35)	81 (1.50)	68,667 (1.22)	0.01	0.01	0.02
**Hyperlipidemia, no. (%)**	34,823 (26.46)	4,454 (26.58)	1,447 (26.86)	1,448,593 (25.70)	0.02	0.02	0.03
**Hypertension, no. (%)**	35,235 (26.77)	4,885 (29.15)	1,663 (30.87)	1,485,407 (26.35)	0.01	0.06	0.10

*. Area Deprivation Index is a measure of socioeconomic disadvantage, with a range from low to high disadvantage of 0 to 100.
†. Data collected within one year before cohort enrollment
std, standard deviation; BMI, body mass index; eGFR, estimated glomerular filtration rate.

Supplementary Table 8. Risks and 12-month burdens of incident post-acute COVID-19 cardiovascular outcomes by care setting of the acute infection compared to contemporary control

Outcome*	Care setting†	Hazard ratio (95% CI) ‡	COVID-19 burden per 1000 persons at 12 months (95% CI) ‡	Contemporary control burden per 1000 persons at 12 months (95% CI) ‡	Burden difference per 1000 persons at 12 months (95% CI) ‡
**Cerebrovascular**	Non-hospitalized	1.30 (1.22, 1.37)	13.79 (13.03, 14.60)	10.67 (10.58, 10.75)	3.13 (2.36, 3.93)
	Hospitalized	2.92 (2.53, 3.37)	30.85 (26.77, 35.53)		20.18 (16.11, 24.86)
	Admitted to intensive care	4.00 (3.19, 5.02)	42.01 (33.60, 52.46)		31.34 (22.94, 41.79)
Stroke	Non-hospitalized	1.23 (1.15, 1.32)	9.79 (9.15, 10.48)	7.94 (7.87, 8.01)	1.85 (1.21, 2.54)
	Hospitalized	3.06 (2.60, 3.59)	24.08 (20.54, 28.22)		16.14 (12.60, 20.28)
	Admitted to intensive care	4.35 (3.38, 5.59)	34.06 (26.58, 43.60)		26.12 (18.64, 35.66)
TIA	Non-hospitalized	1.35 (1.23, 1.47)	5.09 (4.65, 5.57)	3.79 (3.74, 3.84)	1.30 (0.87, 1.79)
	Hospitalized	2.47 (1.95, 3.14)	9.34 (7.36, 11.84)		5.55 (3.58, 8.05)
	Admitted to intensive care	2.83 (1.89, 4.24)	10.69 (7.16, 15.96)		6.91 (3.37, 12.17)
**Dysrhythmia**	Non-hospitalized	1.33 (1.29, 1.38)	39.36 (38.06, 40.70)	29.64 (29.50, 29.78)	9.72 (8.42, 11.06)
	Hospitalized	3.89 (3.55, 4.27)	110.50 (101.23, 120.55)		80.86 (71.59, 90.91)
	Admitted to intensive care	7.93 (7.00, 8.98)	212.25 (189.94, 236.77)		182.61 (160.30, 207.13)
Atrial fibrillation	Non-hospitalized	1.32 (1.26, 1.39)	20.33 (19.35, 21.35)	15.45 (15.34, 15.55)	4.88 (3.90, 5.91)
	Hospitalized	3.94 (3.45, 4.51)	59.50 (52.23, 67.74)		44.05 (36.78, 52.30)
	Admitted to intensive care	7.69 (6.54, 9.03)	112.79 (96.84, 131.16)		97.34 (81.39, 115.72)
Sinus tachycardia	Non-hospitalized	1.37 (1.28, 1.46)	9.48 (8.90, 10.10)	6.95 (6.88, 7.02)	2.53 (1.95, 3.15)
	Hospitalized	5.03 (4.33, 5.83)	34.45 (29.76, 39.86)		27.50 (22.82, 32.91)
	Admitted to intensive care	10.36 (8.35, 12.85)	69.71 (56.59, 85.72)		62.76 (49.64, 78.77)
Sinus bradycardia	Non-hospitalized	1.29 (1.22, 1.37)	10.97 (10.35, 11.62)	8.52 (8.45, 8.60)	2.44 (1.82, 3.09)
	Hospitalized	2.82 (2.41, 3.28)	23.82 (20.46, 27.72)		15.30 (11.94, 19.20)
	Admitted to intensive care	4.81 (3.84, 6.01)	40.31 (32.35, 50.17)		31.79 (23.83, 41.65)
Ventricular arrhythmia	Non-hospitalized	1.35 (1.25, 1.46)	6.89 (6.38, 7.44)	5.09 (5.03, 5.15)	1.80 (1.29, 2.35)
	Hospitalized	3.90 (3.16, 4.81)	19.71 (16.01, 24.24)		14.62 (10.92, 19.15)
	Admitted to intensive care	8.14 (6.32, 10.47)	40.67 (31.75, 52.01)		35.57 (26.66, 46.92)
Atrial flutter	Non-hospitalized	1.33 (1.20, 1.46)	5.31 (4.81, 5.85)		1.31 (0.82, 1.85)

	Hospitalized	3.53 (2.77, 4.49)	14.04 (11.05, 17.83)	4.00 (3.95, 4.05)	10.04 (7.06, 13.83)
	Admitted to intensive care	8.34 (6.45, 10.78)	32.86 (25.52, 42.27)		28.86 (21.52, 38.28)
**Inflammatory heart disease**	Non-hospitalized	1.50 (1.28, 1.76)	1.86 (1.59, 2.19)	1.25 (1.22, 1.28)	0.62 (0.34, 0.94)
	Hospitalized	5.72 (4.02, 8.15)	7.11 (5.00, 10.11)		5.86 (3.75, 8.86)
	Admitted to intensive care	9.87 (5.64, 17.25)	12.22 (7.01, 21.27)		10.98 (5.77, 20.03)
Pericarditis	Non-hospitalized	1.38 (1.17, 1.64)	1.64 (1.39, 1.94)	1.19 (1.16, 1.22)	0.46 (0.20, 0.76)
	Hospitalized	5.34 (3.60, 7.91)	6.32 (4.27, 9.35)		5.13 (3.08, 8.16)
	Admitted to intensive care	9.49 (5.17, 17.41)	11.20 (6.12, 20.46)		10.02 (4.94, 19.27)
Myocarditis	Non-hospitalized	3.47 (2.25, 5.35)	0.25 (0.16, 0.39)	0.07 (0.07, 0.08)	0.18 (0.09, 0.31)
	Hospitalized	12.13 (7.22, 20.36)	0.88 (0.52, 1.47)		0.80 (0.45, 1.40)
	Admitted to intensive care	35.57 (13.56, 93.26)	2.57 (0.98, 6.72)		2.49 (0.91, 6.64)
**Ischemic heart disease**	Non-hospitalized	1.24 (1.18, 1.31)	14.14 (13.42, 14.90)	11.38 (11.30, 11.47)	2.76 (2.03, 3.52)
	Hospitalized	3.76 (3.24, 4.36)	42.13 (36.47, 48.65)		30.75 (25.08, 37.27)
	Admitted to intensive care	5.65 (4.75, 6.72)	62.65 (52.97, 74.03)		51.27 (41.59, 62.64)
Acute coronary disease	Non-hospitalized	1.16 (1.08, 1.24)	8.78 (8.21, 9.40)	7.58 (7.51, 7.66)	1.20 (0.62, 1.81)
	Hospitalized	3.94 (3.28, 4.72)	29.53 (24.68, 35.32)		21.95 (17.09, 27.74)
	Admitted to intensive care	7.05 (5.88, 8.46)	52.27 (43.78, 62.35)		44.68 (36.19, 54.77)
Myocardial infarction	Non-hospitalized	1.08 (0.99, 1.18)	5.12 (4.68, 5.59)	4.73 (4.67, 4.78)	0.39 (-0.04, 0.86)
	Hospitalized	4.35 (3.41, 5.56)	20.42 (16.04, 25.99)		15.69 (11.31, 21.26)
	Admitted to intensive care	8.02 (6.48, 9.91)	37.27 (30.24, 45.90)		32.54 (25.51, 41.17)
Ischemic cardiomyopathy	Non-hospitalized	1.17 (1.06, 1.30)	3.83 (3.45, 4.24)	3.27 (3.22, 3.31)	0.56 (0.18, 0.98)
	Hospitalized	3.34 (2.34, 4.77)	10.88 (7.63, 15.49)		7.61 (4.37, 12.22)
	Admitted to intensive care	4.22 (3.07, 5.79)	13.71 (10.00, 18.77)		10.44 (6.73, 15.50)
Angina	Non-hospitalized	1.40 (1.29, 1.50)	6.82 (6.32, 7.35)	4.89 (4.83, 4.95)	1.93 (1.43, 2.46)
	Hospitalized	3.34 (2.72, 4.10)	16.24 (13.24, 19.91)		11.35 (8.35, 15.02)
	Admitted to intensive care	2.88 (2.00, 4.15)	14.03 (9.77, 20.14)		9.14 (4.88, 15.25)
**Other cardiac disorders**	Non-hospitalized	1.37 (1.31, 1.43)	24.59 (23.53, 25.69)	18.03 (17.92, 18.14)	6.56 (5.50, 7.67)
	Hospitalized	3.94 (3.52, 4.42)	69.19 (61.95, 77.24)		51.16 (43.92, 59.21)
	Admitted to intensive care	6.90 (5.94, 8.00)	117.93 (102.50, 135.51)		99.90 (84.47, 117.48)
Heart failure	Non-hospitalized	1.37 (1.31, 1.44)	22.52 (21.50, 23.59)	16.48 (16.37, 16.58)	6.05 (5.03, 7.11)
	Hospitalized	3.93 (3.50, 4.43)	63.27 (56.43, 70.89)		46.79 (39.96, 54.42)
	Admitted to intensive care	6.05 (5.18, 7.07)	95.67 (82.44, 110.89)		79.19 (65.97, 94.41)
Non-ischemic cardiomyopathy	Non-hospitalized	1.31 (1.22, 1.41)	7.56 (7.03, 8.13)		1.77 (1.24, 2.34)
	Hospitalized	3.20 (2.65, 3.86)	18.40 (15.27, 22.16)		12.61 (9.49, 16.37)

	Admitted to intensive care	6.17 (4.72, 8.07)	35.15 (26.99, 45.72)	5.79 (5.72, 5.85)	29.37 (21.21, 39.93)
Cardiac arrest	Non-hospitalized	0.91 (0.71, 1.17)	0.46 (0.36, 0.59)	0.51 (0.49, 0.53)	-0.04 (-0.15, 0.09)
	Hospitalized	5.62 (3.73, 8.44)	2.85 (1.90, 4.28)		2.34 (1.39, 3.77)
	Admitted to intensive care	29.72 (20.14, 43.86)	14.99 (10.18, 22.05)		14.49 (9.68, 21.54)
Cardiogenic shock	Non-hospitalized	1.15 (0.78, 1.71)	0.41 (0.28, 0.61)	0.36 (0.34, 0.37)	0.05 (-0.08, 0.25)
	Hospitalized	5.28 (3.02, 9.25)	1.87 (1.07, 3.28)		1.52 (0.72, 2.92)
	Admitted to intensive care	17.58 (11.90, 25.98)	6.22 (4.22, 9.18)		5.87 (3.86, 8.83)
**Thrombotic disorders**	Non-hospitalized	1.74 (1.64, 1.84)	12.56 (11.86, 13.31)	7.25 (7.18, 7.32)	5.31 (4.61, 6.06)
	Hospitalized	6.53 (5.75, 7.42)	46.44 (40.98, 52.61)		39.19 (33.73, 45.36)
	Admitted to intensive care	14.51 (12.30, 17.13)	100.24 (85.61, 117.20)		92.99 (78.36, 109.95)
Pulmonary embolism	Non-hospitalized	2.01 (1.84, 2.19)	5.78 (5.31, 6.29)	2.88 (2.84, 2.93)	2.89 (2.42, 3.41)
	Hospitalized	9.42 (8.09, 10.97)	26.83 (23.08, 31.18)		23.95 (20.20, 28.30)
	Admitted to intensive care	21.71 (17.38, 27.12)	60.76 (48.95, 75.30)		57.87 (46.06, 72.42)
Deep vein thrombosis	Non-hospitalized	1.62 (1.49, 1.76)	6.31 (5.82, 6.85)	3.90 (3.85, 3.95)	2.41 (1.92, 2.95)
	Hospitalized	4.44 (3.52, 5.59)	17.17 (13.65, 21.58)		13.27 (9.76, 17.68)
	Admitted to intensive care	9.43 (7.31, 12.16)	36.14 (28.13, 46.39)		32.25 (24.23, 42.49)
Superficial vein thrombosis	Non-hospitalized	1.38 (1.25, 1.52)	3.82 (3.46, 4.21)	2.78 (2.73, 2.82)	1.04 (0.69, 1.43)
	Hospitalized	4.48 (3.66, 5.48)	12.37 (10.12, 15.12)		9.59 (7.34, 12.34)
	Admitted to intensive care	10.32 (7.99, 13.32)	28.27 (21.97, 36.36)		25.50 (19.19, 33.58)
**MACE**	Non-hospitalized	1.26 (1.22, 1.30)	56.12 (54.27, 58.04)	44.83 (44.66, 45.00)	11.29 (9.44, 13.20)
	Hospitalized	2.41 (2.21, 2.63)	104.81 (96.50, 113.79)		59.98 (51.67, 68.96)
	Admitted to intensive care	4.36 (3.87, 4.91)	181.32 (162.79, 201.70)		136.49 (117.96, 156.87)
**Any cardiovascular outcome**	Non-hospitalized	1.39 (1.36, 1.43)	105.38 (102.93, 107.88)	76.88 (76.66, 77.11)	28.50 (26.05, 31.00)
	Hospitalized	3.43 (3.21, 3.66)	239.78 (226.34, 253.89)		162.90 (149.45, 177.01)
	Admitted to intensive care	6.19 (5.61, 6.84)	390.73 (361.71, 421.23)		313.85 (284.82, 344.35)

*. Outcomes were ascertained from day 30 after the initial positive COVID-19 test result until end of follow up.
†. Based on care received within the first 30 days after a positive COVID-19 test result.
‡. Adjustment through inverse probability weighting using predefined variables and algorithmically selected high-dimensional variables.
CI, confidence interval; TIA, transient ischemic attack; MACE, major adverse cardiac events.

Supplementary Table 9. Risks and 12-month burdens of incident post-acute COVID-19 cardiovascular outcomes compared to historical control

Outcome*	Hazard Ratio (95% CI)† COVID-19 vs Historical control	COVID-19 burden per 1000 persons at 12 months (95% CI)†	Historical control burden per 1000 persons at 12 months (95% CI)†	Burden difference per 1000 persons at 12 months (95% CI)†
**Cerebrovascular**	1.49 (1.41, 1.57)	15.95 (15.13, 16.83)	10.77 (10.68, 10.85)	5.19 (4.36, 6.06)
Stroke	1.49 (1.40, 1.59)	11.80 (11.08, 12.56)	7.93 (7.86, 8.00)	3.87 (3.15, 4.62)
TIA	1.41 (1.30, 1.54)	5.62 (5.16, 6.12)	3.98 (3.93, 4.03)	1.64 (1.18, 2.14)
**Dysrhythmia**	1.57 (1.51, 1.62)	49.37 (47.82, 50.97)	31.83 (31.68, 31.98)	17.54 (15.99, 19.14)
Atrial fibrillation	1.62 (1.55, 1.70)	25.99 (24.86, 27.16)	16.11 (16.00, 16.22)	9.88 (8.75, 11.05)
Sinus tachycardia	1.72 (1.63, 1.82)	12.68 (11.97, 13.43)	7.38 (7.31, 7.45)	5.30 (4.59, 6.05)
Sinus bradycardia	1.35 (1.27, 1.42)	13.35 (12.63, 14.11)	9.93 (9.85, 10.02)	3.42 (2.69, 4.18)
Ventricular arrhythmia	1.93 (1.80, 2.07)	9.17 (8.55, 9.83)	4.76 (4.70, 4.82)	4.41 (3.79, 5.07)
Atrial flutter	1.81 (1.67, 1.96)	6.98 (6.43, 7.57)	3.86 (3.81, 3.91)	3.12 (2.57, 3.71)
**Inflammatory heart disease**	2.10 (1.84, 2.39)	2.44 (2.14, 2.78)	1.16 (1.14, 1.19)	1.28 (0.97, 1.62)
Pericarditis	1.88 (1.64, 2.17)	2.13 (1.85, 2.45)	1.13 (1.10, 1.16)	1.00 (0.72, 1.32)
Myocarditis	8.77 (6.15, 12.51)	0.38 (0.27, 0.53)	0.04 (0.04, 0.05)	0.34 (0.22, 0.49)
**Ischemic heart disease**	1.52 (1.40, 1.65)	18.47 (16.99, 20.07)	12.19 (12.10, 12.28)	6.28 (4.80, 7.88)
Acute coronary disease	1.72 (1.55, 1.90)	12.82 (11.60, 14.16)	7.49 (7.41, 7.56)	5.33 (4.11, 6.68)
Myocardial infarction	1.71 (1.59, 1.84)	7.59 (7.05, 8.17)	4.45 (4.39, 4.50)	3.14 (2.60, 3.72)
Ischemic cardiomyopathy	1.62 (1.33, 1.97)	5.47 (4.50, 6.64)	3.37 (3.33, 3.42)	2.09 (1.12, 3.27)
Angina	1.24 (1.15, 1.33)	7.28 (6.78, 7.81)	5.90 (5.83, 5.96)	1.38 (0.88, 1.92)
**Other cardiac disorders**	1.59 (1.53, 1.65)	30.67 (29.48, 31.90)	19.41 (19.29, 19.53)	11.26 (10.07, 12.49)
Heart failure	1.58 (1.51, 1.65)	27.92 (26.79, 29.10)	17.77 (17.66, 17.88)	10.15 (9.02, 11.33)
Non-ischemic cardiomyopathy	1.51 (1.41, 1.61)	9.32 (8.72, 9.96)	6.18 (6.12, 6.25)	3.13 (2.53, 3.77)
Cardiac arrest	2.51 (2.13, 2.97)	1.20 (1.02, 1.42)	0.48 (0.46, 0.50)	0.72 (0.54, 0.94)
Cardiogenic shock	2.52 (1.93, 3.29)	0.87 (0.66, 1.13)	0.34 (0.33, 0.36)	0.52 (0.32, 0.78)
**Thrombotic disorders**	2.66 (2.53, 2.80)	17.07 (16.25, 17.94)	6.45 (6.39, 6.52)	10.62 (9.79, 11.48)
Pulmonary embolism	3.72 (3.46, 4.00)	8.31 (7.75, 8.92)	2.24 (2.20, 2.28)	6.07 (5.51, 6.68)
Deep vein thrombosis	2.17 (2.01, 2.33)	8.04 (7.48, 8.65)	3.72 (3.67, 3.77)	4.32 (3.76, 4.93)
Superficial vein thrombosis	2.09 (1.92, 2.27)	5.37 (4.95, 5.82)	2.58 (2.53, 2.62)	2.80 (2.38, 3.25)

**MACE**	1.60 (1.55, 1.65)	67.67 (65.73, 69.67)	42.81 (42.64, 42.98)	24.86 (22.92, 26.86)
**Any cardiovascular outcome**	1.58 (1.54, 1.62)	121.60 (118.52, 124.76)	78.75 (78.52, 78.98)	42.85 (39.77, 46.00)

*. Outcomes were ascertained from day 30 after the initial positive COVID-19 test result until end of follow up

†. Adjustment through inverse probability weighting using predefined and algorithmically selected high-dimensional variables.

CI, confidence interval; TIA, transient ischemic attack; MACE, major adverse cardiac events.

Supplementary Table 10. Subgroup analyses of the risks of incident post-acute COVID-19 composite cardiovascular outcomes compared to historical control

Risk factors	Hazard ratio (95% CI)* - COVID-19 vs Historical control							
	Cerebro-vascular	Dysrhythmia	Inflammatory heart disease	Ischemic heart disease	Other cardiac disorders	Thrombotic disorders	MACE	Any cardiovascular outcome
**Age**								
≤65	1.35 (1.21, 1.50)	1.50 (1.42, 1.58)	1.97 (1.49, 2.61)	1.44 (1.25, 1.66)	1.39 (1.28, 1.51)	2.38 (2.18, 2.59)	1.40 (1.30, 1.50)	1.49 (1.43, 1.56)
>65	1.49 (1.40, 1.59)	1.52 (1.42, 1.62)	2.13 (1.84, 2.47)	1.52 (1.43, 1.62)	1.67 (1.59, 1.75)	2.79 (2.62, 2.97)	1.65 (1.59, 1.72)	1.64 (1.57, 1.70)
**Race**								
White	1.52 (1.42, 1.62)	1.56 (1.49, 1.62)	2.24 (1.91, 2.62)	1.43 (1.35, 1.51)	1.58 (1.50, 1.66)	2.53 (2.38, 2.69)	1.59 (1.53, 1.65)	1.60 (1.55, 1.65)
Black	1.45 (1.31, 1.60)	1.53 (1.44, 1.64)	1.82 (1.46, 2.28)	1.84 (1.23, 2.76)	1.67 (1.54, 1.80)	2.71 (2.48, 2.97)	1.76 (1.64, 1.88)	1.65 (1.57, 1.73)
**Sex**								
Male	1.47 (1.39, 1.55)	1.58 (1.53, 1.64)	2.10 (1.82, 2.41)	1.49 (1.39, 1.61)	1.63 (1.57, 1.70)	2.69 (2.55, 2.83)	1.56 (1.51, 1.61)	1.61 (1.57, 1.66)
Female	1.13 (0.89, 1.43)	1.42 (1.26, 1.59)	2.10 (1.46, 3.01)	1.26 (1.02, 1.57)	1.25 (1.02, 1.53)	1.81 (1.46, 2.24)	1.38 (1.14, 1.67)	1.40 (1.27, 1.55)
**Obesity**†								
No	1.50 (1.40, 1.62)	1.60 (1.52, 1.69)	2.20 (1.81, 2.69)	1.56 (1.40, 1.75)	1.77 (1.66, 1.88)	2.77 (2.58, 2.98)	1.58 (1.51, 1.64)	1.67 (1.61, 1.73)
Yes	1.50 (1.39, 1.63)	1.45 (1.39, 1.52)	1.97 (1.67, 2.33)	1.37 (1.28, 1.48)	1.46 (1.39, 1.54)	2.48 (2.30, 2.67)	1.56 (1.49, 1.64)	1.53 (1.48, 1.58)
**Smoking**								
No	1.41 (1.34, 1.48)	1.38 (1.35, 1.42)	1.94 (1.71, 2.19)	1.45 (1.38, 1.52)	1.51 (1.46, 1.56)	2.27 (2.16, 2.37)	1.52 (1.48, 1.57)	1.51 (1.48, 1.54)
Yes	1.57 (1.38, 1.78)	1.58 (1.46, 1.72)	2.04 (1.47, 2.84)	1.49 (1.29, 1.72)	1.50 (1.35, 1.67)	2.28 (2.00, 2.60)	1.57 (1.46, 1.68)	1.57 (1.48, 1.67)

**Hypertension**								
No	1.52 (1.42, 1.63)	1.57 (1.51, 1.64)	2.10 (1.78, 2.49)	1.48 (1.25, 1.74)	1.59 (1.51, 1.67)	2.70 (2.54, 2.87)	1.60 (1.54, 1.66)	1.59 (1.54, 1.64)
Yes	1.48 (1.35, 1.61)	1.47 (1.39, 1.55)	2.24 (1.81, 2.77)	1.65 (1.50, 1.83)	1.65 (1.55, 1.76)	2.51 (2.29, 2.74)	1.66 (1.57, 1.75)	1.58 (1.51, 1.64)
**Diabetes**								
No	1.56 (1.45, 1.69)	1.57 (1.51, 1.64)	2.04 (1.69, 2.45)	1.64 (1.38, 1.96)	1.70 (1.60, 1.8)	2.76 (2.58, 2.95)	1.63 (1.57, 1.70)	1.61 (1.56, 1.66)
Yes	1.39 (1.29, 1.49)	1.42 (1.34, 1.50)	1.95 (1.65, 2.31)	1.33 (1.25, 1.42)	1.42 (1.35, 1.49)	2.38 (2.22, 2.56)	1.50 (1.44, 1.56)	1.50 (1.43, 1.58)
**Chronic kidney disease**‡								
No	1.54 (1.44, 1.65)	1.56 (1.49, 1.63)	2.07 (1.74, 2.46)	1.53 (1.31, 1.79)	1.63 (1.55, 1.72)	2.70 (2.54, 2.87)	1.61 (1.55, 1.68)	1.59 (1.54, 1.64)
Yes	1.43 (1.30, 1.56)	1.44 (1.36, 1.52)	2.05 (1.71, 2.47)	1.47 (1.34, 1.60)	1.54 (1.44, 1.64)	2.50 (2.29, 2.74)	1.56 (1.48, 1.64)	1.54 (1.43, 1.65)
**Hyperlipidemia**								
No	1.62 (1.52, 1.74)	1.50 (1.44, 1.57)	2.22 (1.90, 2.61)	1.57 (1.48, 1.67)	1.72 (1.64, 1.80)	2.75 (2.59, 2.93)	1.70 (1.64, 1.76)	1.64 (1.59, 1.68)
Yes	1.34 (1.23, 1.46)	1.49 (1.41, 1.58)	1.67 (1.38, 2.03)	1.38 (1.16, 1.63)	1.32 (1.23, 1.41)	2.43 (2.24, 2.64)	1.41 (1.34, 1.48)	1.45 (1.39, 1.52)
**Cardiovascular disease**								
No	1.43 (1.34, 1.53)	1.54 (1.46, 1.63)	2.03 (1.70, 2.43)	1.42 (1.33, 1.51)	1.58 (1.51, 1.67)	2.64 (2.49, 2.81)	1.60 (1.54, 1.66)	1.58 (1.54, 1.63)
Yes	1.33 (1.21, 1.45)	1.32 (1.24, 1.40)	1.87 (1.59, 2.21)	1.24 (1.16, 1.34)	1.41 (1.32, 1.50)	1.97 (1.80, 2.15)	1.35 (1.29, 1.41)	NA

*. Adjustment through inverse probability weighting using predefined and algorithmically selected high-dimensional variables.
†. Obesity was defined based on baseline BMI>30kg/m^2
‡. Chronic kidney disease was defined based on baseline outpatient estimated Glomerular Filtration Rate< 60 mL/min/1.73 m^2
CI, confidence interval; MACE, major adverse cardiac events, NA, not applicable

Supplementary Table 11. Risks and 12-month burdens of incident post-acute COVID-19 cardiovascular outcomes in participants without any history of cardiovascular outcomes prior to COVID-19 exposure compared to historical control

Outcome*	Hazard Ratio (95% CI)† COVID-19 vs Historical control	COVID-19 burden per 1000 persons at 12 months (95% CI)†	Historical control burden per 1000 persons at 12 months (95% CI)†	Burden difference per 1000 persons at 12 months (95% CI)†
**Cerebrovascular**	1.43 (1.34, 1.53)	13.45 (12.61, 14.34)	9.40 (9.31, 9.48)	4.05 (3.22, 4.94)
Stroke	1.42 (1.32, 1.53)	9.78 (9.08, 10.55)	6.90 (6.83, 6.97)	2.88 (2.18, 3.65)
TIA	1.39 (1.25, 1.55)	4.49 (4.03, 5.00)	3.22 (3.17, 3.27)	1.27 (0.81, 1.78)
**Dysrhythmia**	1.54 (1.46, 1.63)	43.73 (41.42, 46.17)	28.61 (28.46, 28.75)	15.13 (12.81, 17.56)
Atrial fibrillation	1.49 (1.41, 1.58)	20.21 (19.12, 21.37)	13.60 (13.50, 13.71)	6.61 (5.52, 7.76)
Sinus tachycardia	1.72 (1.61, 1.84)	10.83 (10.12, 11.60)	6.31 (6.24, 6.38)	4.52 (3.80, 5.29)
Sinus bradycardia	1.34 (1.25, 1.43)	11.03 (10.29, 11.83)	8.27 (8.19, 8.35)	2.77 (2.02, 3.56)
Ventricular arrhythmia	2.29 (1.72, 3.04)	6.91 (5.21, 9.16)	3.03 (2.98, 3.08)	3.88 (2.18, 6.13)
Atrial flutter	1.88 (1.67, 2.12)	4.46 (3.97, 5.03)	2.37 (2.33, 2.41)	2.09 (1.59, 2.66)
**Inflammatory heart disease**	2.04 (1.71, 2.44)	1.71 (1.43, 2.05)	0.84 (0.81, 0.86)	0.87 (0.59, 1.21)
Pericarditis	1.82 (1.49, 2.21)	1.48 (1.21, 1.79)	0.81 (0.79, 0.84)	0.66 (0.40, 0.98)
Myocarditis	9.36 (6.17, 14.21)	0.30 (0.20, 0.44)	0.03 (0.03, 0.04)	0.27 (0.17, 0.41)
**Ischemic heart disease**	1.42 (1.33, 1.51)	12.92 (12.16, 13.73)	9.13 (9.05, 9.21)	3.79 (3.04, 4.60)
Acute coronary disease	1.62 (1.49, 1.76)	7.92 (7.30, 8.60)	4.90 (4.84, 4.96)	3.02 (2.40, 3.69)
Myocardial infarction	1.58 (1.43, 1.75)	5.12 (4.63, 5.67)	3.24 (3.19, 3.29)	1.89 (1.39, 2.43)
Ischemic cardiomyopathy	1.52 (1.30, 1.78)	2.39 (2.04, 2.79)	1.57 (1.54, 1.61)	0.81 (0.46, 1.22)
Angina	1.21 (1.11, 1.31)	5.82 (5.36, 6.32)	4.81 (4.75, 4.87)	1.01 (0.55, 1.51)
**Other cardiac disorders**	1.58 (1.50, 1.66)	24.45 (23.26, 25.71)	15.53 (15.42, 15.64)	8.92 (7.72, 10.17)
Heart failure	1.60 (1.51, 1.69)	21.41 (20.27, 22.61)	13.45 (13.35, 13.55)	7.96 (6.82, 9.16)
Non-ischemic cardiomyopathy	1.48 (1.34, 1.64)	5.21 (4.71, 5.78)	3.52 (3.47, 3.57)	1.70 (1.19, 2.26)
Cardiac arrest	3.03 (2.06, 4.46)	0.80 (0.54, 1.17)	0.26 (0.25, 0.28)	0.54 (0.28, 0.91)
Cardiogenic shock	2.05 (1.39, 3.04)	0.30 (0.20, 0.44)	0.14 (0.13, 0.15)	0.15 (0.06, 0.29)
**Thrombotic disorders**	2.65 (2.49, 2.81)	14.97 (14.11, 15.87)	5.68 (5.61, 5.75)	9.29 (8.43, 10.19)
Pulmonary embolism	3.95 (3.63, 4.31)	7.24 (6.65, 7.87)	1.84 (1.80, 1.87)	5.40 (4.81, 6.04)
Deep vein thrombosis	2.09 (1.91, 2.29)	6.60 (6.04, 7.21)	3.16 (3.11, 3.21)	3.44 (2.87, 4.05)
Superficial vein thrombosis	2.19 (1.97, 2.44)	3.97 (3.57, 4.42)	1.81 (1.78, 1.85)	2.16 (1.76, 2.61)
**MACE**	1.59 (1.53, 1.65)	57.25 (55.19, 59.39)	36.37 (36.21, 36.53)	20.89 (18.82, 23.02)

**Any cardiovascular outcome**	1.58 (1.54, 1.62)	121.60 (118.52, 124.76)	78.75 (78.52, 78.98)	42.85 (39.77, 46.00)

*. Outcomes were ascertained from day 30 after the initial positive COVID-19 test result until end of follow up

†. Adjustment through inverse probability weighting using predefined and algorithmically selected high-dimensional variables.

CI, confidence interval; TIA, transient ischemic attack; MACE, major adverse cardiac events.

Supplementary Table 12. Demographic and health characteristics of the COVID-19 and historical cohorts by care setting of the acute infection before weighting

Baseline Characteristics	Non-hospitalized (N=131,612)	Hospitalized (N=16,760)	Admitted to intensive care (N=5,388)	Historical control (N=5,859,411)	Absolute standardized difference - Non-hospitalized and Historical control	Absolute standardized difference - Hospitalized and Historical control	Absolute standardized difference - Admitted to intensive care and Historical control
**Age, mean (std), yr**	60.23 (15.72)	68.38 (13.38)	68.95 (12.03)	62.90 (16.48)	0.17	0.37	0.42
**Race, no. (%)**							
White	95,035 (72.21)	10,585 (63.16)	3,358 (62.32)	4,532,542 (77.36)	0.12	0.31	0.33
Black	30,166 (22.92)	5,228 (31.19)	1,719 (31.90)	1,051,656 (17.95)	0.12	0.31	0.33
Other	6,411 (4.87)	947 (5.65)	311 (5.77)	275,213 (4.70)	0.01	0.04	0.05
**Sex, no. (%)**							
Male	116,032 (88.16)	15,774 (94.12)	5,106 (94.77)	5,308,854 (90.60)	0.08	0.13	0.16
Female	15,580 (11.84)	986 (5.88)	282 (5.23)	550,557 (9.40)	0.08	0.13	0.16
**BMI category, no. (%), kg/m^2**							
BMI ≤ 25	17,581 (13.36)	3,571 (21.31)	1,040 (19.30)	1,162,294 (19.84)	0.17	0.04	0.01
25 < BMI ≤ 30	42,041 (31.94)	5,134 (30.63)	1,611 (29.90)	2,296,904 (39.20)	0.15	0.18	0.20
BMI > 30	71,990 (54.70)	8,055 (48.06)	2,737 (50.80)	2,400,213 (40.96)	0.28	0.14	0.20
**Smoking status, no. (%)**							
Never	59,811 (45.45)	7,140 (42.60)	2,176 (40.39)	3,086,138 (52.67)	0.14	0.20	0.25
Former	51,871 (39.41)	6,844 (40.84)	2,369 (43.97)	1,444,066 (24.65)	0.32	0.35	0.42
Current	19,930 (15.14)	2,776 (16.56)	843 (15.65)	1,329,207 (22.69)	0.19	0.15	0.18
**Area Deprivation Index*, mean (std)**	55.63 (18.55)	53.13 (18.70)	54.62 (18.63)	54.62 (19.13)	0.05	0.08	0.00
**Clinical Characteristics**							
**Outpatient encounter†, no. (%)**							
Zero	3,909 (2.97)	185 (1.10)	62 (1.15)	654,793 (11.18)	0.32	0.43	0.43
One	24,764 (18.82)	1,311 (7.82)	408 (7.57)	1,775,335 (30.30)	0.27	0.60	0.61
Two or more	102,939 (78.21)	15,264 (91.07)	4,918 (91.28)	3,429,283 (58.53)	0.43	0.81	0.82

**Long-term care, no. (%)**	3,884 (2.95)	1,726 (10.30)	497 (9.22)	47,384 (0.81)	0.16	0.42	0.39
**eGFR, mean (std), ml/min/1.73m^2**	79.47 (21.31)	68.26 (26.13)	65.07 (26.79)	79.31 (19.95)	0.01	0.48	0.60
**Systolic blood pressure, mean (std), mmHg**	132.35 (11.69)	134.38 (11.83)	134.57 (12.14)	132.63 (12.63)	0.02	0.14	0.16
**Diastolic blood pressure, mean (std), mmHg**	78.54 (7.37)	77.06 (7.46)	76.99 (7.73)	77.53 (7.91)	0.13	0.06	0.07
**Cancer, no. (%)**	9,315 (7.08)	2,411 (14.39)	798 (14.81)	345,608 (5.90)	0.05	0.28	0.30
**Chronic kidney disease, no. (%)**	21,928 (16.66)	5,946 (35.48)	2,135 (39.63)	902,029 (15.40)	0.03	0.47	0.56
**Chronic lung disease, no. (%)**	17,740 (13.48)	4,208 (25.11)	1,521 (28.23)	652,107 (11.13)	0.07	0.37	0.44
**Dementia, no. (%)**	6,344 (4.82)	2,472 (14.75)	681 (12.64)	191,866 (3.27)	0.08	0.41	0.35
**Diabetes mellitus type 2, no. (%)**	39,029 (29.66)	7,689 (45.88)	2,689 (49.91)	1,330,448 (22.71)	0.16	0.50	0.59
**Dysautonomia, no. (%)**	2,120 (1.61)	525 (3.13)	167 (3.10)	72,975 (1.25)	0.03	0.13	0.13
**Hyperlipidemia, no. (%)**	44,815 (34.05)	5,535 (33.03)	1,877 (34.84)	1,810,965 (30.91)	0.07	0.05	0.08
**Hypertension, no. (%)**	34,060 (25.88)	5,290 (31.56)	1,710 (31.74)	1,540,087 (26.28)	0.01	0.12	0.12

*. Area Deprivation Index is a measure of socioeconomic disadvantage, with a range from low to high disadvantage of 0 to 100.
†. Data collected within one year before cohort enrollment
std, standard deviation; BMI, body mass index; eGFR, estimated glomerular filtration rate.

Supplementary Table 13. Demographic and health characteristics of the COVID-19 and historical cohorts by care setting of the acute infection after weighting

Baseline Characteristics	Non-hospitalized (N=131,612)	Hospitalized (N=16,760)	Admitted to intensive care (N=5,388)	Historical control (N=5,859,411)	Absolute standardized difference - Non-hospitalized and Historical control	Absolute standardized difference - Hospitalized and Historical control	Absolute standardized difference - Admitted to intensive care and Historical control
**Age, mean (std), yr**	63.06 (16.29)	63.21 (15.88)	63.46 (16.07)	63.32 (16.30)	0.02	0.01	0.01
**Race, no. (%)**							
White	99,846 (75.86)	12,406 (74.02)	3,951 (73.33)	4,485,028 (76.54)	0.02	0.06	0.07
Black	25,339 (19.25)	3,512 (20.96)	1,073 (19.92)	1,094,538 (18.68)	0.01	0.06	0.03
Other	6,427 (4.88)	842 (5.02)	364 (6.75)	279,904 (4.78)	0.00	0.01	0.08
**Sex, no. (%)**							
Male	118,151 (89.77)	15,285 (91.20)	4,982 (92.46)	5,285,657 (90.21)	0.01	0.03	0.08
Female	13,461 (10.23)	1,475 (8.80)	406 (7.54)	573,754 (9.79)	0.01	0.03	0.08
**BMI category, no. (%), kg/m^2**							
BMI ≤ 25	25,091 (19.06)	3,299 (19.68)	965 (17.91)	1,138,015 (19.42)	0.01	0.01	0.04
25 < BMI ≤ 30	47,358 (35.98)	5,713 (34.09)	1,917 (35.57)	2,247,787 (38.36)	0.05	0.09	0.06
BMI > 30	59,164 (44.95)	7,748 (46.23)	2,506 (46.52)	2,473,609 (42.22)	0.06	0.08	0.09
**Smoking status, no. (%)**							
Never	54,307 (41.26)	6,714 (40.06)	2,236 (41.50)	2,501,968 (42.70)	0.03	0.05	0.02
Former	48,812 (37.09)	5,972 (35.63)	1,963 (36.43)	2,111,732 (36.04)	0.02	0.01	0.01
Current	28,493 (21.65)	4,073 (24.30)	1,189 (22.07)	1,245,711 (21.26)	0.01	0.07	0.02
**Area Deprivation Index*, mean (std)**	54.95 (18.93)	55.04 (19.30)	55.96 (19.24)	54.65 (19.03)	0.02	0.02	0.07
**Clinical Characteristics**							
**Outpatient encounter†, no. (%)**							
Zero	15,124 (11.49)	1,664 (9.93)	464 (8.62)	671,078 (11.45)	0.00	0.05	0.09
One	40,762 (30.97)	5,617 (33.51)	1,910 (35.45)	1,834,582 (31.31)	0.01	0.05	0.09
Two or more	75,728 (57.54)	9,479 (56.56)	3,014 (55.93)	3,353,751 (57.24)	0.01	0.01	0.03
**Long-term care, no. (%)**	1,191 (0.91)	240 (1.43)	87 (1.61)	41,485 (0.71)	0.02	0.07	0.08

**eGFR, mean (std), ml/min/1.73m^2**	78.55 (20.59)	77.93 (21.54)	77.72 (21.68)	78.63 (20.31)	0.00	0.03	0.04
**Systolic blood pressure, mean (std), mmHg**	132.58 (12.42)	133.18 (12.41)	134.03 (12.22)	132.61 (12.33)	0.01	0.05	0.12
**Diastolic blood pressure, mean (std), mmHg**	77.82 (7.62)	78.22 (7.61)	78.30 (7.71)	77.80 (7.56)	0.00	0.06	0.07
**Cancer, no. (%)**	8,092 (6.15)	1,193 (7.12)	370 (6.87)	337,912 (5.77)	0.02	0.06	0.05
**Chronic kidney disease, no. (%)**	22,753 (17.29)	3,175 (18.94)	1,037 (19.24)	978,404 (16.70)	0.02	0.06	0.07
**Chronic lung disease, no. (%)**	14,908 (11.33)	2,071 (12.36)	639 (11.87)	641,898 (10.96)	0.01	0.04	0.03
**Dementia, no. (%)**	4,445 (3.38)	765 (4.56)	254 (4.72)	185,919 (3.17)	0.01	0.07	0.08
**Diabetes mellitus type 2, no. (%)**	30,642 (23.28)	4,359 (26.01)	1,474 (27.35)	1,337,411 (22.83)	0.01	0.07	0.10
**Dysautonomia, no. (%)**	1,683 (1.28)	226 (1.35)	81 (1.50)	71,778 (1.23)	0.00	0.01	0.02
**Hyperlipidemia, no. (%)**	34,823 (26.46)	4,454 (26.58)	1,447 (26.86)	1,512,841 (25.82)	0.01	0.02	0.02
**Hypertension, no. (%)**	35,235 (26.77)	4,885 (29.15)	1,663 (30.87)	1,541,377 (26.31)	0.01	0.06	0.10

*. Area Deprivation Index is a measure of socioeconomic disadvantage, with a range from low to high disadvantage of 0 to 100.
†. Data collected within one year before cohort enrollment
std, standard deviation; BMI, body mass index; eGFR, estimated glomerular filtration rate.

Supplementary Table 14. Risks and 12-month burdens of incident post-acute COVID-19 cardiovascular outcomes by care setting of the acute infection compared to historical control

Outcome*	Care setting†	Hazard ratio (95% CI) ‡	COVID-19 burden per 1000 persons at 12 months (95% CI) ‡	Historical control burden per 1000 persons at 12 months (95% CI) ‡	Burden difference per 1000 persons at 12 months (95% CI) ‡
**Cerebrovascular**	Non-hospitalized	1.26 (1.19, 1.34)	13.79 (13.03, 14.60)	10.95 (10.86, 11.04)	2.84 (2.08, 3.65)
	Hospitalized	2.85 (2.46, 3.29)	30.85 (26.77, 35.53)		19.90 (15.82, 24.58)
	Admitted to intensive care	3.90 (3.10, 4.89)	42.01 (33.60, 52.46)		31.06 (22.65, 41.51)
Stroke	Non-hospitalized	1.21 (1.13, 1.30)	9.79 (9.15, 10.48)	8.10 (8.02, 8.17)	1.69 (1.05, 2.38)
	Hospitalized	3.00 (2.55, 3.52)	24.08 (20.54, 28.22)		15.98 (12.44, 20.12)
	Admitted to intensive care	4.26 (3.31, 5.48)	34.06 (26.58, 43.60)		25.96 (18.48, 35.50)
TIA	Non-hospitalized	1.28 (1.17, 1.40)	5.09 (4.65, 5.57)	3.99 (3.94, 4.04)	1.11 (0.67, 1.59)
	Hospitalized	2.35 (1.85, 2.98)	9.34 (7.36, 11.84)		5.35 (3.38, 7.85)
	Admitted to intensive care	2.69 (1.80, 4.03)	10.69 (7.16, 15.96)		6.71 (3.17, 11.97)
**Dysrhythmia**	Non-hospitalized	1.24 (1.20, 1.28)	39.36 (38.06, 40.70)	31.92 (31.77, 32.07)	7.44 (6.14, 8.79)
	Hospitalized	3.61 (3.29, 3.96)	110.50 (101.23, 120.55)		78.58 (69.32, 88.63)
	Admitted to intensive care	7.35 (6.49, 8.33)	212.25 (189.94, 236.78)		180.33 (158.03, 204.85)
Atrial fibrillation	Non-hospitalized	1.25 (1.19, 1.31)	20.33 (19.35, 21.35)	16.29 (16.19, 16.40)	4.03 (3.06, 5.06)
	Hospitalized	3.73 (3.27, 4.27)	59.50 (52.23, 67.74)		43.20 (35.94, 51.45)
	Admitted to intensive care	7.29 (6.20, 8.56)	112.79 (96.83, 131.17)		96.49 (80.55, 114.87)
Sinus tachycardia	Non-hospitalized	1.28 (1.20, 1.36)	9.48 (8.90, 10.10)	7.42 (7.35, 7.49)	2.06 (1.48, 2.68)
	Hospitalized	4.71 (4.06, 5.46)	34.45 (29.76, 39.86)		27.03 (22.34, 32.44)
	Admitted to intensive care	9.70 (7.82, 12.03)	69.71 (56.59, 85.72)		62.28 (49.17, 78.30)
Sinus bradycardia	Non-hospitalized	1.13 (1.07, 1.20)	10.97 (10.35, 11.62)	9.69 (9.61, 9.77)	1.27 (0.66, 1.93)
	Hospitalized	2.48 (2.12, 2.89)	23.82 (20.46, 27.72)		14.13 (10.77, 18.03)
	Admitted to intensive care	4.22 (3.38, 5.29)	40.31 (32.35, 50.17)		30.62 (22.66, 40.48)
Ventricular arrhythmia	Non-hospitalized	1.42 (1.32, 1.54)	6.89 (6.38, 7.44)	4.85 (4.80, 4.91)	2.04 (1.53, 2.59)
	Hospitalized	4.09 (3.32, 5.05)	19.71 (16.01, 24.25)		14.85 (11.16, 19.39)
	Admitted to intensive care	8.53 (6.63, 10.98)	40.67 (31.74, 52.03)		35.81 (26.90, 47.16)
Atrial flutter	Non-hospitalized	1.34 (1.21, 1.47)	5.31 (4.81, 5.85)		1.33 (0.84, 1.87)

	Hospitalized	3.55 (2.79, 4.51)	14.04 (11.05, 17.83)	3.98 (3.93, 4.03)	10.06 (7.08, 13.85)
	Admitted to intensive care	8.38 (6.49, 10.84)	32.86 (25.51, 42.28)		28.88 (21.54, 38.30)
**Inflammatory heart disease**	Non-hospitalized	1.56 (1.33, 1.83)	1.86 (1.59, 2.19)	1.20 (1.17, 1.22)	0.67 (0.39, 0.99)
	Hospitalized	5.96 (4.19, 8.49)	7.11 (5.00, 10.11)		5.91 (3.80, 8.91)
	Admitted to intensive care	10.28 (5.87, 17.98)	12.22 (7.01, 21.29)		11.03 (5.82, 20.08)
Pericarditis	Non-hospitalized	1.41 (1.19, 1.67)	1.64 (1.39, 1.94)	1.16 (1.14, 1.19)	0.48 (0.22, 0.78)
	Hospitalized	5.44 (3.68, 8.06)	6.32 (4.27, 9.34)		5.15 (3.11, 8.18)
	Admitted to intensive care	9.67 (5.27, 17.76)	11.2 (6.12, 20.47)		10.04 (4.96, 19.30)
Myocarditis	Non-hospitalized	5.67 (3.65, 8.81)	0.25 (0.16, 0.39)	0.04 (0.04, 0.05)	0.21 (0.12, 0.34)
	Hospitalized	19.82 (11.73, 33.48)	0.88 (0.52, 1.48)		0.83 (0.48, 1.43)
	Admitted to intensive care	58.11 (22.08, 152.93)	2.57 (0.98, 6.74)		2.52 (0.94, 6.67)
**Ischemic heart disease**	Non-hospitalized	1.14 (1.08, 1.20)	14.14 (13.42, 14.90)	12.38 (12.29, 12.47)	1.76 (1.04, 2.52)
	Hospitalized	3.46 (2.98, 4.00)	42.13 (36.47, 48.66)		29.75 (24.09, 36.27)
	Admitted to intensive care	5.19 (4.37, 6.17)	62.65 (52.97, 74.03)		50.27 (40.59, 61.65)
Acute coronary disease	Non-hospitalized	1.16 (1.08, 1.24)	8.78 (8.21, 9.40)	7.60 (7.53, 7.67)	1.19 (0.61, 1.80)
	Hospitalized	3.93 (3.28, 4.72)	29.53 (24.67, 35.33)		21.93 (17.08, 27.73)
	Admitted to intensive care	7.04 (5.87, 8.44)	52.27 (43.77, 62.36)		44.67 (36.18, 54.75)
Myocardial infarction	Non-hospitalized	1.14 (1.04, 1.25)	5.12 (4.68, 5.59)	4.49 (4.44, 4.55)	0.62 (0.19, 1.10)
	Hospitalized	4.58 (3.59, 5.85)	20.42 (16.03, 25.99)		15.93 (11.54, 21.49)
	Admitted to intensive care	8.44 (6.82, 10.44)	37.27 (30.23, 45.90)		32.78 (25.75, 41.40)
Ischemic cardiomyopathy	Non-hospitalized	1.09 (0.98, 1.21)	3.83 (3.45, 4.24)	3.52 (3.47, 3.57)	0.31 (-0.07, 0.73)
	Hospitalized	3.10 (2.17, 4.43)	10.88 (7.63, 15.49)		7.36 (4.12, 11.97)
	Admitted to intensive care	3.92 (2.85, 5.38)	13.71 (10.00, 18.77)		10.19 (6.48, 15.25)
Angina	Non-hospitalized	1.13 (1.05, 1.22)	6.82 (6.33, 7.34)	6.02 (5.96, 6.09)	0.79 (0.30, 1.32)
	Hospitalized	2.71 (2.21, 3.33)	16.24 (13.24, 19.91)		10.22 (7.22, 13.88)
	Admitted to intensive care	2.34 (1.62, 3.37)	14.03 (9.77, 20.14)		8.01 (3.74, 14.11)
**Other cardiac disorders**	Non-hospitalized	1.27 (1.21, 1.32)	24.59 (23.53, 25.7)	19.47 (19.36, 19.58)	5.12 (4.06, 6.22)
	Hospitalized	3.65 (3.25, 4.09)	69.19 (61.95, 77.24)		49.72 (42.48, 57.77)
	Admitted to intensive care	6.38 (5.50, 7.41)	117.93 (102.49, 135.52)		98.46 (83.03, 116.04)
Heart failure	Non-hospitalized	1.26 (1.20, 1.32)	22.52 (21.50, 23.59)	17.92 (17.81, 18.03)	4.60 (3.58, 5.67)
	Hospitalized	3.61 (3.21, 4.07)	63.27 (56.43, 70.90)		45.34 (38.51, 52.97)
	Admitted to intensive care	5.56 (4.76, 6.50)	95.67 (82.44, 110.90)		77.75 (64.52, 92.97)
Non-ischemic cardiomyopathy	Non-hospitalized	1.22 (1.13, 1.31)	7.56 (7.03, 8.13)		1.35 (0.82, 1.91)
	Hospitalized	2.98 (2.47, 3.60)	18.4 (15.27, 22.16)		12.19 (9.06, 15.95)

	Admitted to intensive care	5.74 (4.39, 7.51)	35.15 (26.99, 45.73)	6.21 (6.15, 6.28)	28.94 (20.78, 39.51)
Cardiac arrest	Non-hospitalized	0.94 (0.73, 1.20)	0.46 (0.36, 0.60)	0.49 (0.48, 0.51)	-0.03 (-0.13, 0.1)
	Hospitalized	5.77 (3.84, 8.68)	2.85 (1.90, 4.28)		2.36 (1.40, 3.79)
	Admitted to intensive care	30.53 (20.68, 45.07)	14.99 (10.18, 22.06)		14.5 (9.69, 21.55)
Cardiogenic shock	Non-hospitalized	1.20 (0.81, 1.77)	0.41 (0.28, 0.61)	0.34 (0.33, 0.36)	0.07 (-0.07, 0.26)
	Hospitalized	5.50 (3.14, 9.62)	1.87 (1.07, 3.28)		1.53 (0.73, 2.94)
	Admitted to intensive care	18.28 (12.37, 27.03)	6.22 (4.21, 9.19)		5.88 (3.87, 8.84)
**Thrombotic disorders**	Non-hospitalized	1.94 (1.83, 2.05)	12.56 (11.86, 13.31)	6.50 (6.43, 6.57)	6.06 (5.36, 6.81)
	Hospitalized	7.29 (6.42, 8.29)	46.44 (40.98, 52.61)		39.94 (34.48, 46.11)
	Admitted to intensive care	16.20 (13.72, 19.12)	100.24 (85.60, 117.21)		93.74 (79.11, 110.70)
Pulmonary embolism	Non-hospitalized	2.55 (2.34, 2.78)	5.78 (5.30, 6.29)	2.27 (2.23, 2.31)	3.51 (3.04, 4.02)
	Hospitalized	11.98 (10.28, 13.96)	26.83 (23.07, 31.19)		24.56 (20.82, 28.91)
	Admitted to intensive care	27.61 (22.10, 34.50)	60.76 (48.93, 75.32)		58.49 (46.68, 73.04)
Deep vein thrombosis	Non-hospitalized	1.69 (1.55, 1.83)	6.31 (5.81, 6.85)	3.75 (3.70, 3.80)	2.56 (2.07, 3.10)
	Hospitalized	4.61 (3.66, 5.81)	17.17 (13.65, 21.58)		13.42 (9.90, 17.83)
	Admitted to intensive care	9.80 (7.59, 12.64)	36.14 (28.12, 46.40)		32.39 (24.38, 42.64)
Superficial vein thrombosis	Non-hospitalized	1.47 (1.34, 1.63)	3.82 (3.46, 4.21)	2.59 (2.55, 2.63)	1.22 (0.87, 1.62)
	Hospitalized	4.80 (3.92, 5.87)	12.37 (10.12, 15.12)		9.78 (7.53, 12.53)
	Admitted to intensive care	11.05 (8.56, 14.27)	28.27 (21.96, 36.36)		25.68 (19.38, 33.76)
**MACE**	Non-hospitalized	1.30 (1.26, 1.35)	56.12 (54.27, 58.04)	43.39 (43.22, 43.56)	12.73 (10.88, 14.65)
	Hospitalized	2.50 (2.29, 2.72)	104.81 (96.50, 113.80)		61.42 (53.11, 70.40)
	Admitted to intensive care	4.51 (4.01, 5.08)	181.32 (162.78, 201.71)		137.93 (119.40, 158.31)
**Any cardiovascular outcome**	Non-hospitalized	1.35 (1.32, 1.38)	105.38 (102.93, 107.88)	79.21 (78.98, 79.45)	26.16 (23.72, 28.66)
	Hospitalized	3.32 (3.11, 3.55)	239.78 (226.33, 253.90)		160.57 (147.12, 174.68)
	Admitted to intensive care	6.00 (5.44, 6.63)	390.73 (361.70, 421.24)		311.52 (282.49, 342.02)

*. Outcomes were ascertained from day 30 after the initial positive COVID-19 test result until end of follow up.
†. Based on care received within the first 30 days after a positive COVID-19 test result.
‡. Adjustment through inverse probability weighting using predefined variables and algorithmically selected high-dimensional variables.
CI, confidence interval; TIA, transient ischemic attack; MACE, major adverse cardiac events.

Supplementary Table 15. Difference-in-differences analyses of incident rate ratios of cardiovascular outcomes before and after exposure to COVID-19 compared to contemporary control

Outcome*	Incident rate ratio of COVID-19 compared to contemporary control (95% CI) †		Ratio of incident rate ratios of post/pre COVID-19 exposure periods (95% CI) ‡
	Pre-COVID-19 exposure period	Post-COVID-19 exposure period	
**Cerebrovascular**	1.00 (0.95, 1.05)	1.50 (1.42, 1.58)	1.49 (1.39, 1.62)
Stroke	1.00 (0.94, 1.06)	1.48 (1.40, 1.59)	1.48 (1.36, 1.63)
TIA	1.01 (0.93, 1.07)	1.43 (1.32, 1.57)	1.43 (1.29, 1.60)
**Dysrhythmia**	1.00 (0.97, 1.03)	1.68 (1.61, 1.77)	1.68 (1.59, 1.78)
Atrial fibrillation	1.00 (0.95, 1.05)	1.65 (1.58, 1.73)	1.65 (1.55, 1.77)
Sinus tachycardia	1.00 (0.94, 1.06)	1.83 (1.73, 1.95)	1.83 (1.69, 1.99)
Sinus bradycardia	1.00 (0.96, 1.05)	1.52 (1.44, 1.61)	1.51 (1.41, 1.63)
Ventricular arrhythmia	1.00 (0.93, 1.07)	1.95 (1.64, 2.37)	1.95 (1.62, 2.40)
Atrial flutter	1.00 (0.93, 1.07)	1.75 (1.62, 1.91)	1.75 (1.58, 1.95)
**Inflammatory heart disease**	1.00 (0.88, 1.12)	1.99 (1.75, 2.29)	1.99 (1.69, 2.40)
Pericarditis	1.00 (0.88, 1.12)	1.83 (1.60, 2.13)	1.83 (1.54, 2.22)
Myocarditis	1.03 (0.58, 1.69)	5.32 (3.78, 7.66)	5.21 (2.83, 10.05)
**Ischemic heart disease**	1.00 (0.95, 1.05)	1.61 (1.49, 1.77)	1.61 (1.47, 1.79)
Acute coronary disease	1.00 (0.94, 1.06)	1.68 (1.52, 1.87)	1.68 (1.50, 1.90)
Myocardial infarction	1.01 (0.93, 1.08)	1.58 (1.47, 1.72)	1.58 (1.43, 1.76)
Ischemic cardiomyopathy	1.00 (0.92, 1.08)	1.70 (1.41, 2.10)	1.70 (1.39, 2.12)
Angina	1.00 (0.94, 1.06)	1.48 (1.38, 1.60)	1.48 (1.36, 1.64)
**Other cardiac disorders**	1.00 (0.96, 1.04)	1.71 (1.64, 1.78)	1.71 (1.61, 1.81)
Heart failure	1.00 (0.96, 1.04)	1.71 (1.64, 1.79)	1.71 (1.61, 1.82)
Non-ischemic cardiomyopathy	1.00 (0.94, 1.06)	1.60 (1.50, 1.72)	1.60 (1.47, 1.75)
Cardiac arrest	1.03 (0.67, 1.50)	2.55 (2.04, 3.28)	2.49 (1.60, 4.01)
Cardiogenic shock	1.01 (0.70, 1.42)	2.36 (1.83, 3.15)	2.34 (1.54, 3.69)
**Thrombotic disorders**	1.00 (0.95, 1.05)	2.34 (2.22, 2.47)	2.34 (2.18, 2.52)
Pulmonary embolism	1.02 (0.92, 1.12)	2.88 (2.68, 3.10)	2.82 (2.51, 3.20)

Deep vein thrombosis	1.02 (0.93, 1.10)	2.01 (1.87, 2.18)	1.99 (1.79, 2.22)
Superficial vein thrombosis	1.01 (0.91, 1.10)	1.88 (1.74, 2.05)	1.87 (1.66, 2.12)
**MACE**	1.00 (0.95, 1.05)	1.54 (1.50, 1.59)	1.54 (1.46, 1.64)
**Any cardiovascular outcome**	1.00 (0.97, 1.03)	1.64 (1.60, 1.69)	1.64 (1.58, 1.70)

*. Outcomes for pre-exposure period were ascertained until day 30 before the initial positive COVID-19 test result. Outcomes for post-exposure period were ascertained from day 30 after the initial positive COVID-19 test result until end of follow up.
†. Adjustment through weighting using predefined and algorithmically selected high-dimensional variables during pre-exposure period.
‡. Incidence rate ratio in pre-exposure period served as the referent.
CI, confidence interval; TIA, transient ischemic attack; MACE, major adverse cardiac events.

Supplementary Table 16. Difference-in-differences analyses of incident rate ratios of cardiovascular outcomes before and after exposure to COVID-19 by care setting of the acute infection compared to contemporary control

Outcome*	Care setting†	Incident rate ratio of COVID-19 compared to contemporary control (95% CI) ‡		Ratio of incident rate ratios of post/pre COVID-19 exposure periods (95% CI) §
		Pre-COVID-19 exposure period	Post-COVID-19 exposure period	
**Cerebrovascular**	Non-hospitalized	1.00 (0.95, 1.05)	1.31 (1.24, 1.39)	1.30 (1.21, 1.41)
	Hospitalized	1.00 (0.84, 1.17)	2.91 (2.53, 3.38)	2.90 (2.37, 3.60)
	Admitted to intensive care	1.01 (0.75, 1.31)	3.93 (3.14, 4.98)	3.91 (2.78, 5.65)
Stroke	Non-hospitalized	1.00 (0.94, 1.06)	1.24 (1.17, 1.34)	1.24 (1.14, 1.36)
	Hospitalized	1.01 (0.82, 1.20)	3.04 (2.61, 3.60)	3.04 (2.39, 3.90)
	Admitted to intensive care	1.01 (0.71, 1.38)	4.28 (3.34, 5.56)	4.25 (2.87, 6.51)
TIA	Non-hospitalized	1.01 (0.92, 1.09)	1.35 (1.24, 1.48)	1.34 (1.20, 1.53)
	Hospitalized	1.01 (0.80, 1.22)	2.45 (1.94, 3.13)	2.44 (1.83, 3.36)
	Admitted to intensive care	1.02 (0.68, 1.45)	2.76 (1.86, 4.17)	2.72 (1.64, 4.70)
**Dysrhythmia**	Non-hospitalized	1.00 (0.96, 1.04)	1.35 (1.31, 1.40)	1.35 (1.28, 1.43)
	Hospitalized	1.00 (0.88, 1.12)	3.92 (3.57, 4.32)	3.91 (3.39, 4.56)
	Admitted to intensive care	1.01 (0.80, 1.23)	7.96 (7.02, 9.11)	7.89 (6.28, 10.34)
Atrial fibrillation	Non-hospitalized	1.00 (0.94, 1.06)	1.34 (1.28, 1.41)	1.34 (1.24, 1.45)
	Hospitalized	1.01 (0.85, 1.16)	3.93 (3.45, 4.52)	3.92 (3.22, 4.82)
	Admitted to intensive care	1.01 (0.77, 1.27)	7.58 (6.45, 8.99)	7.51 (5.70, 10.31)
Sinus tachycardia	Non-hospitalized	1.00 (0.94, 1.06)	1.38 (1.30, 1.47)	1.38 (1.27, 1.50)
	Hospitalized	1.01 (0.81, 1.21)	5.01 (4.35, 5.86)	5.00 (3.96, 6.44)
	Admitted to intensive care	1.01 (0.71, 1.37)	10.24 (8.31, 12.81)	10.12 (7.06, 15.34)
Sinus bradycardia	Non-hospitalized	1.00 (0.95, 1.05)	1.29 (1.22, 1.37)	1.29 (1.20, 1.40)
	Hospitalized	1.01 (0.80, 1.23)	2.81 (2.42, 3.29)	2.79 (2.19, 3.66)
	Admitted to intensive care	1.01 (0.74, 1.32)	4.77 (3.83, 6.02)	4.74 (3.36, 6.89)
Ventricular arrhythmia	Non-hospitalized	1.01 (0.93, 1.08)	1.37 (1.27, 1.49)	1.36 (1.23, 1.52)
	Hospitalized	1.01 (0.81, 1.21)	3.87 (3.16, 4.81)	3.85 (2.96, 5.14)
	Admitted to intensive care	1.02 (0.49, 1.93)	7.95 (6.22, 10.34)	7.74 (3.98, 16.29)
Atrial flutter	Non-hospitalized	1.00 (0.92, 1.09)	1.34 (1.23, 1.49)	1.34 (1.19, 1.53)
	Hospitalized	1.01 (0.74, 1.32)	3.51 (2.78, 4.50)	3.50 (2.44, 5.08)

	Admitted to intensive care	1.02 (0.69, 1.42)	8.21 (6.39, 10.76)	8.10 (5.36, 12.79)
**Inflammatory heart disease**	Non-hospitalized	1.01 (0.86, 1.15)	1.51(1.30, 1.78)	1.50 (1.23, 1.88)
	Hospitalized	1.01 (0.69, 1.40)	5.64 (4.02, 8.18)	5.62 (3.54, 9.18)
	Admitted to intensive care	1.03 (0.53, 1.86)	9.53 (5.59, 16.86)	9.34 (4.26, 21.38)
Pericarditis	Non-hospitalized	1.01 (0.86, 1.15)	1.39 (1.19, 1.66)	1.39 (1.13, 1.75)
	Hospitalized	1.01 (0.68, 1.41)	5.25 (3.60, 7.93)	5.22 (3.20, 8.93)
	Admitted to intensive care	1.02 (0.50, 1.90)	9.15 (5.12, 16.96)	9.04 (3.92, 21.93)
Myocarditis	Non-hospitalized	1.03 (0.48, 2.00)	3.50 (2.31, 5.47)	3.44 (1.55, 8.13)
	Hospitalized	1.03 (0.34, 2.72)	11.84 (7.21, 20.42)	11.46 (3.85, 37.82)
	Admitted to intensive care	1.07 (0.32, 3.03)	33.61 (13.71, 92.20)	31.94 (7.93, 138.18)
**Ischemic heart disease**	Non-hospitalized	1.00 (0.94, 1.05)	1.26 (1.20, 1.33)	1.26 (1.17, 1.36)
	Hospitalized	1.00 (0.88, 1.12)	3.74 (3.24, 4.37)	3.73 (3.14, 4.55)
	Admitted to intensive care	1.01 (0.79, 1.24)	5.56 (4.69, 6.67)	5.54 (4.24, 7.40)
Acute coronary disease	Non-hospitalized	1.00 (0.93, 1.07)	1.17 (1.10, 1.26)	1.17 (1.07, 1.29)
	Hospitalized	1.01 (0.85, 1.16)	3.91 (3.28, 4.73)	3.90 (3.13, 4.97)
	Admitted to intensive care	1.01 (0.74, 1.32)	6.93 (5.80, 8.39)	6.85 (5.01, 9.88)
Myocardial infarction	Non-hospitalized	1.00 (0.93, 1.07)	1.09 (1.01, 1.20)	1.09 (0.98, 1.23)
	Hospitalized	1.01 (0.82, 1.20)	4.32 (3.41, 5.57)	4.29 (3.24, 5.92)
	Admitted to intensive care	1.01 (0.70, 1.40)	7.90 (6.42, 9.86)	7.79 (5.36, 12.07)
Ischemic cardiomyopathy	Non-hospitalized	1.01 (0.91, 1.11)	1.18 (1.07, 1.32)	1.17 (1.03, 1.36)
	Hospitalized	1.01 (0.80, 1.23)	3.29 (2.33, 4.75)	3.27 (2.20, 5.03)
	Admitted to intensive care	1.01 (0.69, 1.42)	4.08 (3.00, 5.68)	4.05 (2.58, 6.52)
Angina	Non-hospitalized	1.00 (0.94, 1.07)	1.40 (1.30, 1.51)	1.39 (1.27, 1.55)
	Hospitalized	1.00 (0.85, 1.17)	3.31 (2.72, 4.10)	3.29 (2.60, 4.32)
	Admitted to intensive care	1.01 (0.72, 1.35)	2.82 (1.98, 4.11)	2.81 (1.80, 4.54)
**Other cardiac disorders**	Non-hospitalized	1.00 (0.95, 1.05)	1.39 (1.33, 1.45)	1.39 (1.30, 1.49)
	Hospitalized	1.00 (0.87, 1.14)	3.94 (3.52, 4.44)	3.93 (3.32, 4.70)
	Admitted to intensive care	1.01 (0.82, 1.20)	6.82 (5.89, 7.99)	6.80 (5.41, 8.71)
Heart failure	Non-hospitalized	1.00 (0.96, 1.04)	1.39 (1.33, 1.46)	1.38 (1.30, 1.48)
	Hospitalized	1.00 (0.86, 1.14)	3.93 (3.50, 4.44)	3.92 (3.30, 4.70)
	Admitted to intensive care	1.01 (0.82, 1.20)	5.97 (5.12, 7.04)	5.96 (4.71, 7.62)
Non-ischemic cardiomyopathy	Non-hospitalized	1.00 (0.93, 1.07)	1.32 (1.23, 1.42)	1.32 (1.20, 1.46)
	Hospitalized	1.01 (0.83, 1.18)	3.17 (2.64, 3.85)	3.16 (2.49, 4.09)
	Admitted to intensive care	1.01 (0.74, 1.32)	6.00 (4.63, 7.91)	5.97 (4.15, 8.82)

Cardiac arrest	Non-hospitalized	1.02 (0.61, 1.60)	0.92 (0.73, 1.20)	0.90 (0.54, 1.58)
	Hospitalized	1.03 (0.45, 2.09)	5.54 (3.78, 8.50)	5.38 (2.42, 13.36)
	Admitted to intensive care	1.05 (0.23, 3.83)	29.05 (20.12, 43.80)	27.79 (7.51, 123.38)
Cardiogenic shock	Non-hospitalized	1.02 (0.59, 1.65)	1.16 (0.80, 1.75)	1.15 (0.63, 2.21)
	Hospitalized	1.03 (0.62, 1.60)	5.20 (3.06, 9.32)	5.05 (2.59, 10.77)
	Admitted to intensive care	1.05 (0.28, 3.23)	17.22 (11.92, 26.13)	16.26 (5.09, 60.44)
**Thrombotic disorders**	Non-hospitalized	1.00 (0.94, 1.07)	1.77 (1.67, 1.88)	1.76 (1.63, 1.92)
	Hospitalized	1.00 (0.84, 1.17)	6.55 (5.79, 7.51)	6.55 (5.36, 8.12)
	Admitted to intensive care	1.01 (0.75, 1.31)	14.57 (12.36, 17.40)	14.42 (10.70, 20.45)
Pulmonary embolism	Non-hospitalized	1.00 (0.90, 1.11)	2.04 (1.88, 2.23)	2.04 (1.79, 2.34)
	Hospitalized	1.01 (0.78, 1.25)	9.41 (8.15, 11.06)	9.34 (7.21, 12.51)
	Admitted to intensive care	1.02 (0.62, 1.57)	21.59 (17.39, 27.35)	21.21 (13.17, 36.68)
Deep vein thrombosis	Non-hospitalized	1.00 (0.90, 1.10)	1.64 (1.52, 1.79)	1.64 (1.46, 1.87)
	Hospitalized	1.01 (0.77, 1.28)	4.40 (3.52, 5.60)	4.38 (3.18, 6.17)
	Admitted to intensive care	1.01 (0.68, 1.43)	9.28 (7.24, 12.12)	9.16 (6.04, 14.68)
Superficial vein thrombosis	Non-hospitalized	1.00 (0.91, 1.10)	1.39 (1.27, 1.54)	1.39 (1.22, 1.60)
	Hospitalized	1.01 (0.76, 1.29)	4.44 (3.66, 5.49)	4.43 (3.24, 6.18)
	Admitted to intensive care	1.02 (0.69, 1.43)	10.09 (7.88, 13.20)	9.93 (6.58, 15.67)
**MACE**	Non-hospitalized	1.00 (0.94, 1.06)	1.27 (1.23, 1.32)	1.27 (1.19, 1.36)
	Hospitalized	1.00 (0.85, 1.15)	2.41 (2.21, 2.64)	2.40 (2.05, 2.89)
	Admitted to intensive care	1.01 (0.78, 1.26)	4.30 (3.82, 4.88)	4.27 (3.33, 5.69)
**Any cardiovascular outcome**	Non-hospitalized	1.00 (0.97, 1.03)	1.41 (1.38, 1.45)	1.41 (1.36, 1.47)
	Hospitalized	1.00 (0.90, 1.10)	3.49 (3.26, 3.75)	3.48 (3.12, 3.94)
	Admitted to intensive care	1.00 (0.84, 1.17)	6.32 (5.69, 7.05)	6.28 (5.25, 7.73)

*. Outcomes for pre-exposure period were ascertained until day 30 before the initial positive COVID-19 test result. Outcomes for post-exposure period were ascertained from day 30 after the initial positive COVID-19 test result until end of follow up.
†. Based on care received within the first 30 days after a positive COVID-19 test result.
‡. Adjustment through weighting using predefined and algorithmically selected high-dimensional variables during pre-exposure period.
§. Incidence rate ratio in pre-exposure period served as the referent.
CI, confidence interval; TIA, transient ischemic attack; MACE, major adverse cardiac events.

Supplementary Table 17. Difference-in-differences analyses of incident rate ratios of cardiovascular outcomes before and after exposure to COVID-19 by care setting of the acute infection compared to historical control

Outcome*	Incident rate ratio of COVID-19 compared to historical control (95% CI) †		Ratio of incident rate ratios of post/pre COVID-19 exposure periods (95% CI) ‡
	Pre-COVID-19 exposure period	Post-COVID-19 exposure period	
**Cerebrovascular**	1.00 (0.95, 1.05)	1.45 (1.37, 1.53)	1.45 (1.35, 1.56)
Stroke	1.00 (0.94, 1.06)	1.44 (1.36, 1.54)	1.44 (1.33, 1.58)
TIA	1.01 (0.93, 1.07)	1.36 (1.25, 1.49)	1.35 (1.22, 1.52)
**Dysrhythmia**	1.00 (0.97, 1.03)	1.53 (1.47, 1.61)	1.53 (1.45, 1.63)
Atrial fibrillation	1.00 (0.95, 1.05)	1.52 (1.45, 1.59)	1.52 (1.42, 1.62)
Sinus tachycardia	1.00 (0.94, 1.06)	1.72 (1.63, 1.83)	1.72 (1.59, 1.87)
Sinus bradycardia	1.00 (0.96, 1.05)	1.34 (1.26, 1.42)	1.33 (1.24, 1.43)
Ventricular arrhythmia	1.00 (0.93, 1.07)	2.04 (1.71, 2.48)	2.04 (1.69, 2.51)
Atrial flutter	1.00 (0.93, 1.07)	1.73 (1.60, 1.89)	1.73 (1.57, 1.94)
**Inflammatory heart disease**	1.00 (0.88, 1.12)	2.07 (1.82, 2.38)	2.07 (1.75, 2.49)
Pericarditis	1.00 (0.88, 1.13)	1.86 (1.62, 2.16)	1.86 (1.56, 2.26)
Myocarditis	1.03 (0.58, 1.70)	8.66 (6.1, 12.68)	8.51 (4.62, 16.45)
**Ischemic heart disease**	1.00 (0.95, 1.05)	1.48 (1.37, 1.62)	1.48 (1.35, 1.64)
Acute coronary disease	1.00 (0.94, 1.06)	1.67 (1.52, 1.86)	1.66 (1.49, 1.89)
Myocardial infarction	1.00 (0.93, 1.08)	1.67 (1.55, 1.81)	1.67 (1.51, 1.86)
Ischemic cardiomyopathy	1.00 (0.93, 1.08)	1.57 (1.30, 1.93)	1.57 (1.28, 1.96)
Angina	1.00 (0.94, 1.06)	1.20 (1.12, 1.30)	1.20 (1.10, 1.33)
**Other cardiac disorders**	1.00 (0.96, 1.04)	1.56 (1.50, 1.63)	1.56 (1.48, 1.66)
Heart failure	1.00 (0.96, 1.04)	1.55 (1.49, 1.63)	1.55 (1.47, 1.65)
Non-ischemic cardiomyopathy	1.00 (0.94, 1.06)	1.48 (1.38, 1.59)	1.48 (1.36, 1.62)
Cardiac arrest	1.03 (0.67, 1.49)	2.63 (2.10, 3.37)	2.57 (1.65, 4.12)
Cardiogenic shock	1.01 (0.69, 1.42)	2.45 (1.89, 3.27)	2.43 (1.60, 3.84)
**Thrombotic disorders**	1.00 (0.95, 1.05)	2.55 (2.43, 2.69)	2.55 (2.38, 2.75)
Pulmonary embolism	1.01 (0.91, 1.11)	3.61 (3.37, 3.90)	3.57 (3.16, 4.03)

Deep vein thrombosis	1.01 (0.93, 1.09)	2.05 (1.90, 2.22)	2.03 (1.82, 2.27)
Superficial vein thrombosis	1.01 (0.91, 1.10)	1.99 (1.83, 2.17)	1.97 (1.75, 2.24)
**MACE**	1.00 (0.95, 1.05)	1.58 (1.54, 1.64)	1.58 (1.50, 1.68)
**Any cardiovascular outcome**	1.00 (0.97, 1.03)	1.58 (1.54, 1.63)	1.58 (1.53, 1.65)

*. Outcomes for pre-exposure period were ascertained until day 30 before the initial positive COVID-19 test result. Outcomes for post-exposure period were ascertained from day 30 after the initial positive COVID-19 test result until end of follow up.
†. Adjustment through weighting using predefined and algorithmically selected high-dimensional variables during pre-exposure period.
‡. Incidence rate ratio in pre-exposure period served as the referent.
CI, confidence interval; TIA, transient ischemic attack; MACE, major adverse cardiac events.

Supplementary Table 18. Difference-in-differences analyses of incident rate ratios of cardiovascular outcomes before and after exposure to COVID-19 by care setting of the acute infection compared to historical control

Outcome*	Care setting†	Incident rate ratio of COVID-19 compared to historical control (95% CI) ‡		Ratio of incident rate ratios of post/pre COVID-19 exposure periods (95% CI) §
		Pre-COVID-19 exposure period	Post-COVID-19 exposure period	
**Cerebrovascular**	Non-hospitalized	1.00 (0.95, 1.05)	1.27 (1.20, 1.35)	1.27 (1.18, 1.37)
	Hospitalized	1.00 (0.84, 1.17)	2.83 (2.46, 3.28)	2.82 (2.30, 3.50)
	Admitted to intensive care	1.01 (0.75, 1.31)	3.82 (3.06, 4.84)	3.81 (2.70, 5.49)
Stroke	Non-hospitalized	1.00 (0.94, 1.06)	1.22 (1.14, 1.31)	1.21 (1.12, 1.34)
	Hospitalized	1.01 (0.82, 1.20)	2.98 (2.55, 3.52)	2.97 (2.34, 3.82)
	Admitted to intensive care	1.01 (0.71, 1.38)	4.19 (3.27, 5.44)	4.16 (2.82, 6.38)
TIA	Non-hospitalized	1.01 (0.92, 1.09)	1.28 (1.18, 1.41)	1.28 (1.14, 1.45)
	Hospitalized	1.01 (0.80, 1.22)	2.32 (1.85, 2.97)	2.31 (1.73, 3.18)
	Admitted to intensive care	1.01 (0.68, 1.44)	2.62 (1.77, 3.96)	2.59 (1.56, 4.48)
**Dysrhythmia**	Non-hospitalized	1.00 (0.96, 1.04)	1.25 (1.21, 1.30)	1.25 (1.19, 1.32)
	Hospitalized	1.00 (0.88, 1.12)	3.63 (3.31, 4.00)	3.62 (3.13, 4.22)
	Admitted to intensive care	1.01 (0.80, 1.23)	7.37 (6.50, 8.43)	7.31 (5.82, 9.57)
Atrial fibrillation	Non-hospitalized	1.00 (0.94, 1.06)	1.27 (1.21, 1.33)	1.26 (1.17, 1.37)
	Hospitalized	1.01 (0.85, 1.16)	3.72 (3.26, 4.28)	3.71 (3.05, 4.56)
	Admitted to intensive care	1.01 (0.77, 1.27)	7.16 (6.10, 8.50)	7.10 (5.39, 9.74)
Sinus tachycardia	Non-hospitalized	1.00 (0.94, 1.06)	1.29 (1.21, 1.38)	1.29 (1.19, 1.41)
	Hospitalized	1.01 (0.81, 1.21)	4.68 (4.06, 5.48)	4.67 (3.70, 6.02)
	Admitted to intensive care	1.01 (0.71, 1.37)	9.57 (7.77, 11.98)	9.46 (6.60, 14.35)
Sinus bradycardia	Non-hospitalized	1.00 (0.95, 1.05)	1.14 (1.08, 1.21)	1.13 (1.05, 1.23)
	Hospitalized	1.01 (0.80, 1.23)	2.47 (2.13, 2.89)	2.45 (1.92, 3.22)
	Admitted to intensive care	1.01 (0.74, 1.32)	4.19 (3.36, 5.29)	4.16 (2.95, 6.05)
Ventricular arrhythmia	Non-hospitalized	1.01 (0.93, 1.08)	1.43 (1.33, 1.56)	1.43 (1.29, 1.59)
	Hospitalized	1.01 (0.81, 1.21)	4.05 (3.31, 5.04)	4.03 (3.09, 5.38)
	Admitted to intensive care	1.02 (0.49, 1.93)	8.33 (6.52, 10.83)	8.10 (4.15, 17.09)
Atrial flutter	Non-hospitalized	1.00 (0.92, 1.09)	1.35 (1.23, 1.50)	1.35 (1.19, 1.54)
	Hospitalized	1.01 (0.74, 1.32)	3.52 (2.79, 4.52)	3.51 (2.45, 5.10)

	Admitted to intensive care	1.02 (0.69, 1.42)	8.25 (6.41, 10.80)	8.13 (5.38, 12.83)
**Inflammatory heart disease**	Non-hospitalized	1.00 (0.86, 1.15)	1.57 (1.35, 1.86)	1.56 (1.28, 1.96)
	Hospitalized	1.01 (0.69, 1.40)	5.87 (4.19, 8.51)	5.84 (3.68, 9.56)
	Admitted to intensive care	1.02 (0.52, 1.83)	9.92 (5.82, 17.55)	9.83 (4.48, 22.50)
Pericarditis	Non-hospitalized	1.00 (0.86, 1.15)	1.42 (1.21, 1.70)	1.41 (1.15, 1.78)
	Hospitalized	1.01 (0.68, 1.41)	5.35 (3.67, 8.08)	5.31 (3.26, 9.09)
	Admitted to intensive care	1.02 (0.50, 1.90)	9.31 (5.22, 17.28)	9.20 (3.99, 22.35)
Myocarditis	Non-hospitalized	1.03 (0.48, 2.01)	5.7 (3.72, 9.01)	5.59 (2.56, 13.25)
	Hospitalized	1.04 (0.34, 2.73)	19.31 (11.67, 33.26)	18.63 (6.30, 62.69)
	Admitted to intensive care	1.08 (0.32, 3.04)	54.65 (22.43, 150.89)	52.01 (12.83, 224.22)
**Ischemic heart disease**	Non-hospitalized	1.00 (0.94, 1.05)	1.15 (1.10, 1.22)	1.15 (1.07, 1.24)
	Hospitalized	1.00 (0.88, 1.12)	3.43 (2.98, 4.00)	3.42 (2.88, 4.17)
	Admitted to intensive care	1.01 (0.79, 1.24)	5.10 (4.31, 6.12)	5.08 (3.89, 6.79)
Acute coronary disease	Non-hospitalized	1.00 (0.93, 1.07)	1.17 (1.10, 1.26)	1.17 (1.06, 1.29)
	Hospitalized	1.00 (0.85, 1.16)	3.90 (3.27, 4.71)	3.88 (3.12, 4.96)
	Admitted to intensive care	1.01 (0.74, 1.33)	6.91 (5.78, 8.36)	6.83 (4.99, 9.85)
Myocardial infarction	Non-hospitalized	1.00 (0.93, 1.08)	1.15 (1.06, 1.26)	1.15 (1.03, 1.29)
	Hospitalized	1.01 (0.82, 1.20)	4.54 (3.59, 5.85)	4.51 (3.41, 6.22)
	Admitted to intensive care	1.01 (0.70, 1.40)	8.30 (6.75, 10.37)	8.19 (5.63, 12.70)
Ischemic cardiomyopathy	Non-hospitalized	1.01 (0.91, 1.11)	1.09 (0.99, 1.22)	1.09 (0.95, 1.26)
	Hospitalized	1.01 (0.80, 1.23)	3.05 (2.16, 4.40)	3.03 (2.04, 4.67)
	Admitted to intensive care	1.01 (0.69, 1.42)	3.78 (2.78, 5.26)	3.75 (2.39, 6.04)
Angina	Non-hospitalized	1.00 (0.94, 1.06)	1.13 (1.06, 1.23)	1.13 (1.03, 1.25)
	Hospitalized	1.01 (0.85, 1.17)	2.69 (2.20, 3.32)	2.67 (2.11, 3.50)
	Admitted to intensive care	1.01 (0.72, 1.35)	2.29 (1.60, 3.33)	2.28 (1.46, 3.68)
**Other cardiac disorders**	Non-hospitalized	1.00 (0.95, 1.05)	1.28 (1.23, 1.34)	1.28 (1.20, 1.37)
	Hospitalized	1.00 (0.87, 1.14)	3.64 (3.25, 4.10)	3.63 (3.06, 4.34)
	Admitted to intensive care	1.01 (0.82, 1.20)	6.30 (5.44, 7.38)	6.28 (5.00, 8.04)
Heart failure	Non-hospitalized	1.00 (0.96, 1.04)	1.27 (1.22, 1.34)	1.27 (1.20, 1.35)
	Hospitalized	1.00 (0.87, 1.14)	3.60 (3.21, 4.07)	3.59 (3.02, 4.31)
	Admitted to intensive care	1.01 (0.82, 1.20)	5.48 (4.70, 6.46)	5.47 (4.32, 6.99)
Non-ischemic cardiomyopathy	Non-hospitalized	1.00 (0.93, 1.07)	1.23 (1.14, 1.32)	1.22 (1.11, 1.36)
	Hospitalized	1.01 (0.83, 1.18)	2.94 (2.46, 3.58)	2.93 (2.32, 3.80)
	Admitted to intensive care	1.01 (0.74, 1.32)	5.58 (4.30, 7.36)	5.55 (3.85, 8.20)

Cardiac arrest	Non-hospitalized	1.02 (0.61, 1.60)	0.95 (0.75, 1.23)	0.93 (0.56, 1.62)
	Hospitalized	1.03 (0.45, 2.09)	5.68 (3.88, 8.72)	5.52 (2.48, 13.70)
	Admitted to intensive care	1.04 (0.23, 3.84)	29.77 (20.65, 44.91)	28.48 (7.71, 126.58)
Cardiogenic shock	Non-hospitalized	1.02 (0.59, 1.65)	1.20 (0.84, 1.81)	1.18 (0.65, 2.30)
	Hospitalized	1.03 (0.62, 1.61)	5.39 (3.19, 9.68)	5.23 (2.67, 11.19)
	Admitted to intensive care	1.05 (0.28, 3.24)	17.86 (12.4, 27.11)	16.85 (5.28, 62.66)
**Thrombotic disorders**	Non-hospitalized	1.00 (0.94, 1.07)	1.97 (1.86, 2.09)	1.96 (1.81, 2.14)
	Hospitalized	1.00 (0.84, 1.17)	7.30 (6.46, 8.36)	7.29 (5.97, 9.04)
	Admitted to intensive care	1.01 (0.75, 1.31)	16.23 (13.77, 19.40)	16.07 (11.91, 22.80)
Pulmonary embolism	Non-hospitalized	1.00 (0.90, 1.11)	2.59 (2.39, 2.83)	2.59 (2.27, 2.97)
	Hospitalized	1.01 (0.78, 1.25)	11.93 (10.35, 14.03)	11.86 (9.16, 15.85)
	Admitted to intensive care	1.02 (0.62, 1.57)	27.40 (22.11, 34.75)	26.94 (16.67, 46.54)
Deep vein thrombosis	Non-hospitalized	1.00 (0.91, 1.10)	1.71 (1.58, 1.86)	1.71 (1.51, 1.94)
	Hospitalized	1.01 (0.77, 1.28)	4.57 (3.65, 5.81)	4.55 (3.30, 6.40)
	Admitted to intensive care	1.01 (0.68, 1.43)	9.63 (7.51, 12.58)	9.50 (6.27, 15.23)
Superficial vein thrombosis	Non-hospitalized	1.00 (0.91, 1.10)	1.49 (1.35, 1.65)	1.48 (1.31, 1.71)
	Hospitalized	1.01 (0.76, 1.29)	4.75 (3.92, 5.87)	4.73 (3.46, 6.61)
	Admitted to intensive care	1.01 (0.69, 1.42)	10.79 (8.43, 14.12)	10.69 (7.08, 16.89)
**MACE**	Non-hospitalized	1.00 (0.94, 1.06)	1.31 (1.27, 1.36)	1.31 (1.23, 1.41)
	Hospitalized	1.00 (0.85, 1.15)	2.49 (2.28, 2.73)	2.48 (2.12, 2.98)
	Admitted to intensive care	1.01 (0.78, 1.26)	4.45 (3.95, 5.04)	4.41 (3.44, 5.87)
**Any cardiovascular outcome**	Non-hospitalized	1.00 (0.97, 1.03)	1.37 (1.34, 1.40)	1.37 (1.32, 1.42)
	Hospitalized	1.00 (0.90, 1.10)	3.37 (3.16, 3.63)	3.37 (3.01, 3.81)
	Admitted to intensive care	1.00 (0.84, 1.17)	6.11 (5.50, 6.82)	6.07 (5.07, 7.48)

*. Outcomes for pre-exposure period were ascertained until day 30 before the initial positive COVID-19 test result. Outcomes for post-exposure period were ascertained from day 30 after the initial positive COVID-19 test result until end of follow up.
†. Based on care received within the first 30 days after a positive COVID-19 test result.
‡. Adjustment through weighting using predefined and algorithmically selected high-dimensional variables during pre-exposure period.
§. Incidence rate ratio in pre-exposure period served as the referent.
CI, confidence interval; TIA, transient ischemic attack; MACE, major adverse cardiac events.

Supplementary Table 19. Sensitivity analysis for MACE and any cardiovascular outcome compared to contemporary and historical controls

Analysis	MACE Hazard ratio (95% CI)*		Any cardiovascular outcome Hazard ratio (95% CI)*	
	**Contemporary**	**Historical**	**Contemporary**	**Historical**
Used 300 algorithmically selected high dimensional variables	1.56 (1.51, 1.61)	1.61 (1.56, 1.66)	1.64 (1.59, 1.69)	1.59 (1.55, 1.64)
Did not use any high dimensional variables	1.46 (1.42, 1.51)	1.55 (1.50, 1.59)	1.66 (1.62, 1.69)	1.65 (1.61, 1.68)
Doubly robust	1.42 (1.38, 1.47)	1.47 (1.43, 1.52)	1.56 (1.53, 1.60)	1.52 (1.48, 1.55)

*. Outcomes were ascertained from day 30 after the initial positive COVID-19 test result until end of follow up.
CI, confidence interval; MACE, major adverse cardiac events.

Supplementary Table 20. Sensitivity analyses for MACE and any cardiovascular outcome by care setting of the acute infection compared to contemporary and historical controls

Analysis	Care Setting†	MACE Hazard ratio (95% CI)*		Any cardiovascular outcome Hazard ratio (95% CI)*	
		Contemporary	Historical	Contemporary	Historical
Used 300 algorithmically selected high dimensional variables	Non-hospitalized	1.26 (1.22, 1.30)	1.30 (1.26, 1.35)	1.39 (1.36, 1.43)	1.35 (1.32, 1.39)
	Hospitalized	2.42 (2.21, 2.64)	2.50 (2.29, 2.73)	3.43 (3.21, 3.66)	3.33 (3.12, 3.56)
	Admitted to intensive care	4.36 (3.88, 4.91)	4.51 (4.01, 5.08)	6.20 (5.61, 6.84)	6.02 (5.46, 6.65)
Did not use any high dimensional variables	Non-hospitalized	1.26 (1.22, 1.31)	1.34 (1.29, 1.38)	1.40 (1.36, 1.43)	1.39 (1.36, 1.43)
	Hospitalized	2.42 (2.22, 2.64)	2.56 (2.35, 2.79)	3.44 (3.22, 3.67)	3.42 (3.20, 3.66)
	Admitted to intensive care	4.37 (3.88, 4.92)	4.62 (4.11, 5.21)	6.21 (5.63, 6.86)	6.18 (5.60, 6.82)
Doubly robust	Non-hospitalized	1.25 (1.21, 1.29)	1.30 (1.25, 1.34)	1.40 (1.37, 1.44)	1.36 (1.32, 1.39)
	Hospitalized	2.36 (2.16, 2.57)	2.44 (2.24, 2.66)	3.44 (3.22, 3.67)	3.31 (3.12, 3.56)
	Admitted to intensive care	4.38 (3.91, 4.91)	4.55 (4.06, 5.10)	6.41 (5.83, 7.05)	6.21 (5.65, 6.82)

*. Outcomes were ascertained from day 30 after the initial positive COVID-19 test result until end of follow up.
†. Based on care received within the first 30 days after a positive COVID-19 test result.
CI, confidence interval; MACE, major adverse cardiac events.

Supplementary Table 21. Risks of incident post-acute COVID-19 myocarditis and pericarditis compared to contemporary control in analyses where cohort participants are censored at time of vaccination

Outcome*	Hazard Ratio (95% CI)†	COVID-19 burden per 1000 persons at 12 months (95% CI) †	Contemporary control burden per 1000 persons at 12 months (95% CI) †	Burden difference per 1000 persons at 12 months (95% CI) †
	COVID-19 vs Contemporary control			
Myocarditis	5.31 (3.75, 7.53)	0.37 (0.26, 0.53)	0.07 (0.06, 0.08)	0.30 (0.19, 0.46)
Pericarditis	1.84 (1.59, 2.12)	2.11 (1.83, 2.43)	1.15 (1.12, 1.18)	0.96 (0.68, 1.28)

*. Outcomes were ascertained from day 30 after the initial positive COVID-19 test result until the first occurrence of COVID-19 vaccination or end of follow up.

†. Adjustment through inverse probability weighting using predefined and algorithmically selected high-dimensional variables.

Before cohort enrollment, 347 (0.23%) of the COVID-19 participants and 9,170 (0.16%) of the contemporary control participants received COVID-19 vaccine. In total, 95,223 (61.93%) participants in COVID-19 group received COVID-19 vaccine (42,065 (44.18%), 45,450 (47.73%) and 7708 (8.09%) received BNT162b2, mRNA-1273 and Ad26.COV2.S, respectively) and 3,173,169 (56.29%) participants in contemporary group received COVID-19 vaccine (1,349,844 (42.54%), 1,609,399 (50.72%) and 213,926 (6.74%) received BNT162b2, mRNA-1273 and Ad26.COV2.S, respectively) before end of follow up.

CI, confidence interval

Supplementary Table 22. Risks of incident post-acute COVID-19 myocarditis and pericarditis compared to contemporary control in analyses adjusting for time varying COVID-19 vaccination

Outcome*	Hazard Ratio (95% CI)†	COVID-19 burden per 1000 persons at 12 months (95% CI) †	Contemporary control burden per 1000 persons at 12 months (95% CI) †	Burden difference per 1000 persons at 12 months (95% CI) †
	COVID-19 vs Contemporary control			
Myocarditis	5.34 (3.76, 7.60)	0.50 (0.35, 0.71)	0.09 (0.08, 1.11)	0.41 (0.26, 0.62)
Pericarditis	1.83 (1.59, 2.11)	2.24 (1.94, 2.58)	1.22 (1.18, 1.27)	1.01 (0.71, 1.36)

*. Outcomes were ascertained from day 30 after the initial positive COVID-19 test result until end of follow up.

†. Adjustment through inverse probability weighting using predefined and algorithmically selected high-dimensional variables.

Before cohort enrollment, 347 (0.23%) of the COVID-19 participants and 9,170 (0.16%) of the contemporary control participants received COVID-19 vaccine. In total, 95,223 (61.93%) participants in COVID-19 group received COVID-19 vaccine (42,065 (44.18%), 45,450 (47.73%) and 7708 (8.09%) received BNT162b2, mRNA-1273 and Ad26.COV2.S, respectively) and 3,173,169 (56.29%) participants in contemporary group received COVID-19 vaccine (1,349,844 (42.54%), 1,609,399 (50.72%) and 213,926 (6.74%) received BNT162b2, mRNA-1273 and Ad26.COV2.S, respectively) before end of follow up.

CI, confidence interval

Supplementary Table 23. Risks of incident post-acute COVID-19 myocarditis and pericarditis compared to historical control in analyses where cohort participants are censored at time of vaccination

Outcome*	Hazard Ratio (95% CI)† COVID-19 vs Historical control	COVID-19 burden per 1000 persons at 12 months (95% CI) †	Historical control burden per 1000 persons at 12 months (95% CI) †	Burden difference per 1000 persons at 12 months (95% CI) †
Myocarditis	8.67 (6.05, 12.42)	0.37 (0.26, 0.53)	0.04 (0.04, 0.05)	0.33 (0.22, 0.49)
Pericarditis	1.87 (1.62, 2.16)	2.11 (1.83, 2.43)	1.13 (1.10, 1.16)	0.98 (0.70, 1.30)

*. Outcomes were ascertained from day 30 after the initial positive COVID-19 test result until the first occurrence of COVID-19 vaccination or end of follow up.

†. Adjustment through inverse probability weighting using predefined and algorithmically selected high-dimensional variables.

Before cohort enrollment, 347 (0.23%) of the COVID-19 participants received COVID-19 vaccine. 95,223 (61.93%) participants in COVID-19 group received COVID-19 vaccine (42,065 (44.18%), 45,450 (47.73%) and 7708 (8.09%) received BNT162b2, mRNA-1273 and Ad26.COV2.S, respectively) before end of follow up.

CI, confidence interval

Supplementary Table 24. Risks of incident post-acute COVID-19 myocarditis and pericarditis compared to historical control in analyses adjusting for time varying COVID-19 vaccination

Outcome*	Hazard Ratio (95% CI)†	COVID-19 burden per 1000 persons at 12 months (95% CI) †	Historical control burden per 1000 persons at 12 months (95% CI) †	Burden difference per 1000 persons at 12 months (95% CI) †
	COVID-19 vs Historical control			
Myocarditis	9.85 (6.35, 15.28)	0.50 (0.35, 0.71)	0.04 (0.04, 0.05)	0.46 (0.31, 0.67)
Pericarditis	1.67 (1.42, 1.97)	2.24 (1.94, 2.58)	1.11 (1.08, 1.15)	1.12 (0.82, 1.46)

*. Outcomes were ascertained from day 30 after the initial positive COVID-19 test result until end of follow up.
†. Adjustment through inverse probability weighting using predefined and algorithmically selected high-dimensional variables.
Before cohort enrollment, 347 (0.23%) of the COVID-19 participants received COVID-19 vaccine. 95,223 (61.93%) participants in COVID-19 group received COVID-19 vaccine (42,065 (44.18%), 45,450 (47.73%) and 7708 (8.09%) received BNT162b2, mRNA-1273 and Ad26.COV2.S, respectively) before end of follow up.
CI, confidence interval

Supplementary Table 25. Positive and negative outcome controls

Outcome*	Hazard Ratio (95% CI)†	
	COVID-19 vs contemporary control (as the reference)	COVID-19 vs historical control (as the reference)
**Positive outcome control**		
Fatigue	1.81 (1.75, 1.88)	1.87 (1.80, 1.94)
**Negative outcome controls**		
Hypertrichosis	0.92 (0.69, 1.23)	0.89 (0.67, 1.19)
Melanoma in situ	1.02 (0.98, 1.06)	1.03 (0.99, 1.07)
Sickle cell trait	1.04 (0.99, 1.10)	1.09 (0.84, 1.42)
Perforation of the tympanic membrane	0.98 (0.80, 1.20)	0.98 (0.94, 1.02)
Malignant neoplasm of the tongue	0.98 (0.92, 1.04)	0.93 (0.88, 1.00)
B-cell Lymphoma	1.04 (0.98, 1.10)	1.10 (0.83, 1.46)
Hodgkin's Lymphoma	1.08 (0.85, 1.39)	1.08 (0.84, 1.38)

*. Outcomes were ascertained from day 30 after the initial positive COVID-19 test result until end of follow up.
†. Adjustment through inverse probability weighting using predefined and algorithmically selected high-dimensional variables.
CI, confidence interval

Supplementary Table 26. Negative exposure control; risks and 12-month burdens of cardiovascular outcomes of those vaccinated for influenza on even-numbered days compared to those vaccinated on odd-numbered days

Outcome*	Hazard Ratio (95% CI)* Influenza vaccination on even days vs odd days	Influenza vaccination on even days burden per 1000 persons at 12 months (95% CI) †	Influenza vaccination on odd days burden per 1000 persons at 12 months (95% CI) †	Absolute burden difference per 1000 persons at 12 months (95% CI) †
**Cerebrovascular**	1.01 (0.96, 1.06)	16.14 (15.36, 16.96)	15.98 (15.69, 16.28)	0.15 (-0.63, 0.97)
Stroke	1.04 (0.98, 1.10)	11.94 (11.27, 12.65)	11.51 (11.27, 11.76)	0.43 (-0.24, 1.13)
TIA	0.98 (0.90, 1.06)	6.22 (5.74, 6.73)	6.35 (6.16, 6.53)	-0.13 (-0.61, 0.38)
**Dysrhythmia**	0.98 (0.95, 1.01)	47.16 (45.80, 48.56)	48.16 (47.64, 48.68)	-1.00 (-2.36, 0.41)
Atrial fibrillation	0.97 (0.93, 1.01)	23.48 (22.53, 24.47)	24.25 (23.89, 24.62)	-0.77 (-1.72, 0.22)
Sinus tachycardia	1.01 (0.95, 1.07)	11.54 (10.89, 12.23)	11.46 (11.21, 11.70)	0.09 (-0.56, 0.78)
Sinus bradycardia	0.98 (0.93, 1.03)	15.33 (14.58, 16.13)	15.67 (15.38, 15.96)	-0.33 (-1.09, 0.46)
Ventricular arrhythmia	0.99 (0.93, 1.06)	9.08 (8.50, 9.69)	9.17 (8.95, 9.39)	-0.10 (-0.67, 0.52)
Atrial flutter	1.01 (0.93, 1.09)	6.90 (6.40, 7.44)	6.85 (6.66, 7.04)	0.05 (-0.45, 0.59)
**Inflammatory heart disease**	0.99 (0.87, 1.13)	2.17 (1.90, 2.48)	2.19 (2.08, 2.29)	-0.02 (-0.29, 0.29)
Pericarditis	0.99 (0.86, 1.14)	2.06 (1.79, 2.36)	2.08 (1.97, 2.18)	-0.02 (-0.28, 0.28)
Myocarditis	0.96 (0.54, 1.70)	0.12 (0.07, 0.21)	0.12 (0.10, 0.15)	-0.01 (-0.06, 0.09)
**Ischemic heart disease**	1.01 (0.97, 1.05)	20.59 (19.78, 21.43)	20.42 (20.09, 20.74)	0.17 (-0.64, 1.01)
Acute coronary disease	1.00 (0.95, 1.05)	13.62 (12.96, 14.31)	13.60 (13.34, 13.86)	0.02 (-0.64, 0.71)
Myocardial infarction	0.99 (0.93, 1.06)	8.37 (7.86, 8.91)	8.42 (8.21, 8.63)	-0.05 (-0.56, 0.49)
Ischemic cardiomyopathy	0.94 (0.86, 1.02)	5.44 (5.00, 5.92)	5.81 (5.63, 5.99)	-0.37 (-0.81, 0.11)
Angina	0.97 (0.91, 1.04)	8.50 (7.94, 9.09)	8.77 (8.55, 8.99)	-0.27 (-0.83, 0.32)
**Other cardiac disorders**	1.00 (0.96, 1.04)	29.57 (28.50, 30.68)	29.61 (29.21, 30.02)	-0.04 (-1.12, 1.07)
Heart failure	0.99 (0.95, 1.03)	26.91 (25.89, 27.97)	27.21 (26.82, 27.60)	-0.31 (-1.33, 0.75)
Non-ischemic cardiomyopathy	1.02 (0.96, 1.09)	10.15 (9.53, 10.80)	9.92 (9.68, 10.15)	0.23 (-0.38, 0.89)
Cardiac arrest	0.98 (0.81, 1.17)	1.02 (0.85, 1.23)	1.05 (0.97, 1.12)	-0.02 (-0.19, 0.18)
Cardiogenic shock	0.95 (0.75, 1.21)	0.68 (0.54, 0.87)	0.72 (0.65, 0.78)	-0.04 (-0.18, 0.15)
**Thrombotic disorders**	1.00 (0.95, 1.06)	11.30 (10.65, 11.99)	11.26 (11.02, 11.51)	0.04 (-0.61, 0.72)
Pulmonary embolism	1.05 (0.96, 1.15)	4.85 (4.43, 5.30)	4.63 (4.47, 4.79)	0.22 (-0.20, 0.67)
Deep vein thrombosis	0.99 (0.91, 1.08)	5.85 (5.39, 6.35)	5.91 (5.73, 6.08)	-0.06 (-0.52, 0.44)
Superficial vein thrombosis	0.98 (0.90, 1.08)	4.54 (4.14, 4.98)	4.62 (4.47, 4.78)	-0.08 (-0.48, 0.36)

**MACE**	1.01 (0.98, 1.04)	49.88 (48.53, 51.27)	49.27 (48.77, 49.78)	0.61 (-0.74, 2.00)
**Any cardiovascular outcome**	1.00 (0.98, 1.02)	102.73 (100.68, 104.83)	102.75 (101.99, 103.52)	-0.02 (-2.07, 2.07)

*. Outcomes were ascertained from day 30 after the Influenza vaccination until end of follow up
†. Adjustment through inverse probability weighting using predefined and algorithmically selected high-dimensional variables.
CI, confidence interval; TIA, transient ischemic attack; MACE, major adverse cardiac events.
